# Enantioselective Organophotocatalytic Telescoped Synthesis of a Chiral Privileged Active Pharmaceutical Ingredient

**DOI:** 10.1002/chem.202200164

**Published:** 2022-05-04

**Authors:** Fabian Herbrik, Miguel Sanz, Alessandra Puglisi, Sergio Rossi, Maurizio Benaglia

**Affiliations:** ^1^ Dipartimento di Chimica Università degli Studi di Milano Via Camillo Golgi 19 20133 Milano Italy; ^2^ Taros Chemicals GmbH & Co. KG Emil-Figge-Strasse 76 A 44227 Dortmund Germany

**Keywords:** chiral API, enantioselective catalysis, flow chemistry, organophotoredox catalysis, telescoped process

## Abstract

The continuous flow, enantioselective, organophotoredox catalytic asymmetric alkylation of aldehydes was studied, by using a homemade, custom‐designed photoreactor for reactions under cryogenic conditions. Going from microfluidic conditions up to a 10 mL mesofluidic reactor, an increase of productivity by almost 18000 % compared to the batch reaction was demonstrated. Finally, for the first time, a stereoselective photoredox organocatalytic continuous flow reaction in a fully telescoped process for an active pharmaceutical ingredient (API)synthesis was successfully achieved. The final process consists of four units of operation: visible light‐driven asymmetric catalytic benzylation under continuous flow, inline continuous work‐up, neutralisation and a final oxidative amidation step afforded the pharmaceutically active molecule in 95 % e.e.

## Graphical abstract

**An efficient in‐flow** catalytic enantioselective organophotoredox alkylation of aldehydes has been successfully developed (up to 98 % e.e.) and applied to the fully telescoped synthesis of a chiral active pharmaceutical ingredient (API), consisting of four units of operation: in‐flow asymmetric benzylation, inline continuous work‐up, neutralisation and oxidative amidation. By using flow technologies, the productivity was increased by almost 200 times compared to the batch reaction.

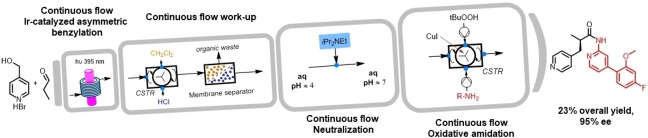

Continuous flow technologies have emerged as powerful tool for the preparation of highly functionalized molecules.^[1]^ Really efficient telescoped processes^[2]^ aimed to the synthesis and even the production of active pharmaceutical ingredients,^[3]^ and some remarkable examples on automated organic synthesis have been reported.^[4]^

However, in the case of chiral molecules, most of the works relate to the in‐flow synthesis of the racemic product, while reports of in continuo enantioselective catalytic synthesis are very rare, calling for new contributions in the field.^[5]^

If enantioselective organophotoredox catalysis is exploited in the synthesis of the target molecule, the development of efficient in flow asymmetric catalytic processes is a necessity.^[6]^ Generally, the bulk of the volume of a photochemical reaction under batch conditions does not receive efficient irradiation; continuous flow reactors often exhibit a two orders of magnitude higher surface‐to‐volume ratio.^[7]^ Seeberger in his famed review “A hitchhikers guide to flow chemistry” defines a 0.1 % transmission cut‐off as a guiding rule at which a photochemical reaction mixture which exhibits a certain transmittance can still operate efficiently.^[1a]^

Combining photochemistry and organocatalysis represents an easy entry to molecules which would otherwise be difficult to attain. Short after the pioneering contribution by MacMillan et al.^[8]^ Zeitler et al. translated the new methodology to be run *in continuo* using custom build coiled polymer tubing wrapped around a compact fluorescence lamp increasing the productivity by roughly ×100.^[9]^ However, examples of asymmetric organocatalyzed photochemical reactions translated into continuous flow are scarce, and, as far as we know, only two other studies have been conducted so far – The *E*/*Z*‐isomerisation, cyclisation and asymmetric reduction cascade with a chiral Brønsted‐acid by Rueping et al. and the asymmetric photo‐α‐oxidation of carbonyl compounds in the presence of a chiral phase transfer catalyst by Meng et al.[^10^, ^11^]

We wish to report here our studies on the in‐flow catalytic asymmetric alkylation of aldehydes, in the attempt to give some insight into the process of scaling‐up an asymmetric catalytic photochemical reaction by 100×: going from microfluidic conditions up to a 10 mL mesofluidic reactor an increase of productivity by almost 18000 % was indeed demonstrated. Finally, for the first time, a stereoselective photoredox organocatalytic continuous flow reaction in a fully telescoped process for an API was successfully achieved with 95 % enantioselectivity.

MacMillan's spin‐center‐shift benzylation^[12]^ was chosen as the ideal reaction for the translation into continuous flow conditions and scale‐up (Scheme 1). After reacting the aldehydes with 4‐pyridyl methyl alcohols the products were reduced to the corresponding alcohols for the HPLC assessment of the enantioselectivity to avoid issues related to lability of the stereogenic center in the α‐position to a carbonyl group.

**Scheme 1:**
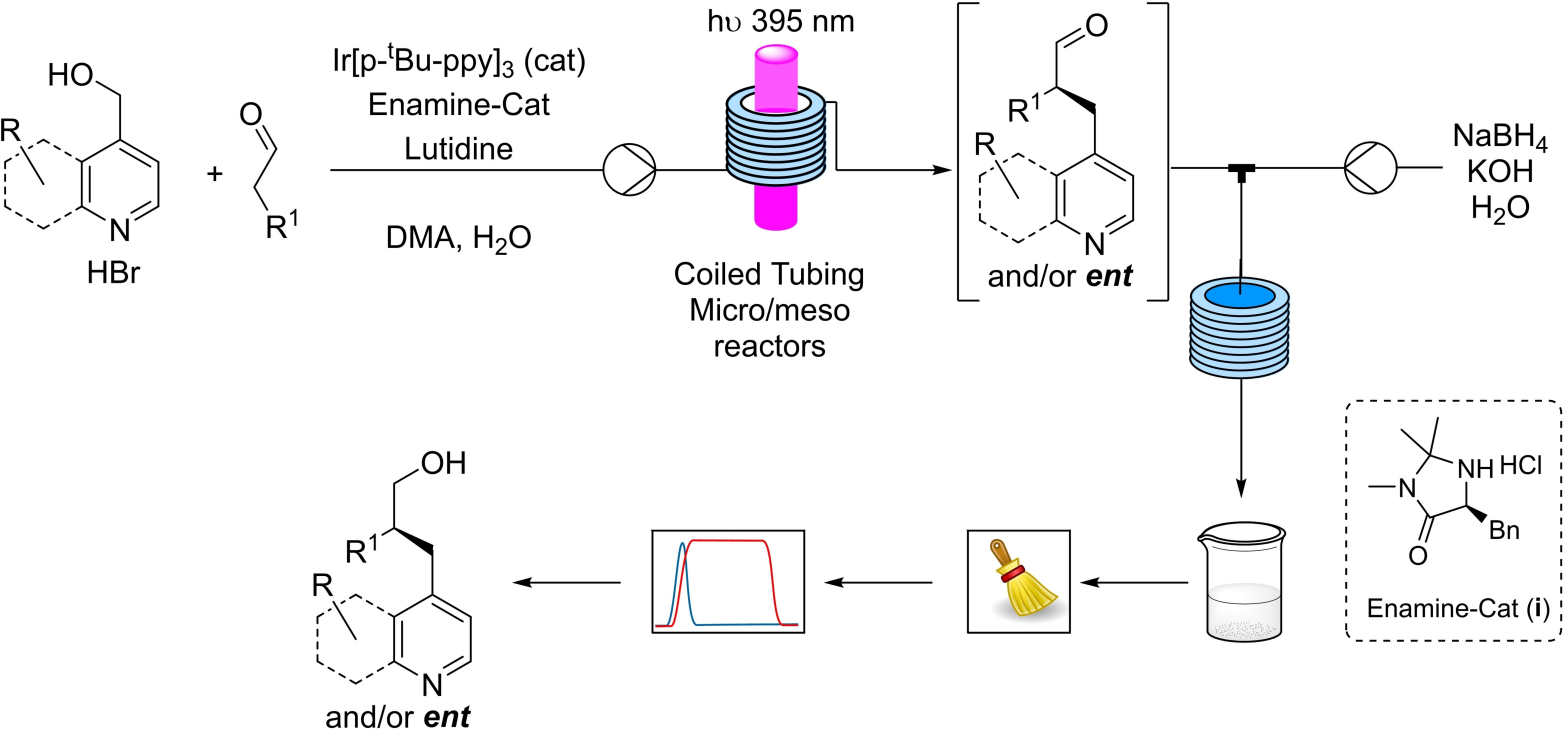
Continuous two‐step enantioselective α‐benzylation of aldehydes and NaBH_4_ reduction, offline collection, chromatographic purification and HPLC‐assessment of the enantiomeric excesses.

After figuring out the best performing LEDs,^[13]^ a homemade, custom designed photoreactor was benchmarked under batch conditions to be later compared to the continuous flow processes (for the description and picture of the photoreactor see the Supporting Information). The reaction of the alcohols **1 a**–**c** and the aldehydes **2 a**–**c** led to products **3 aa**, **3 ba**, **3 ca**, **3 ab**, **3 ac** with moderate yields, after 24 h, due to incomplete conversion, but excellent enantioselectivities, as illustrated in Table 1.

**Table 1:** In batch experiments to generate values for benchmarking the reactor in terms of productivity.

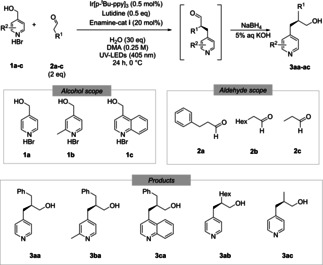			
Entry	Product^[a]^	Yield [%]^[b]^	*ee* [%]^[c]^
1	**3 aa**	43	96
2	**3 ba**	32	97
3	**3 ca**	32	96
4	**3 ab**	31	94
5	**3 ac**	44	96

[a] Conditions: 0 °C, 24 h, c=0.25 M – 250 μmol – 1.0 equiv. **1 a**–**c**, 2.0 equiv. **2 a**–**c**, 0.5 equiv. lutidine, 20 mol% enamine cat., 0.5 mol% Ir[p‐^t^Bu‐ppy]_3_. 5 % KOH, 10 equiv. NaBH_4_. [b] Isolated yield after chromatography. [c] Determined by HPLC on chiral stationary phase.

After a quick aqueous work up with phase separation the samples were purified, evaporated, transferred and gravimetrically evaluated in a semi‐automatized, mostly parallelized workflow that was initially designed to purify and evaluate hundreds of molecules per day in library synthesis.^[14]^ This workflow is illustrated in Figure 1.

**Figure 1:**
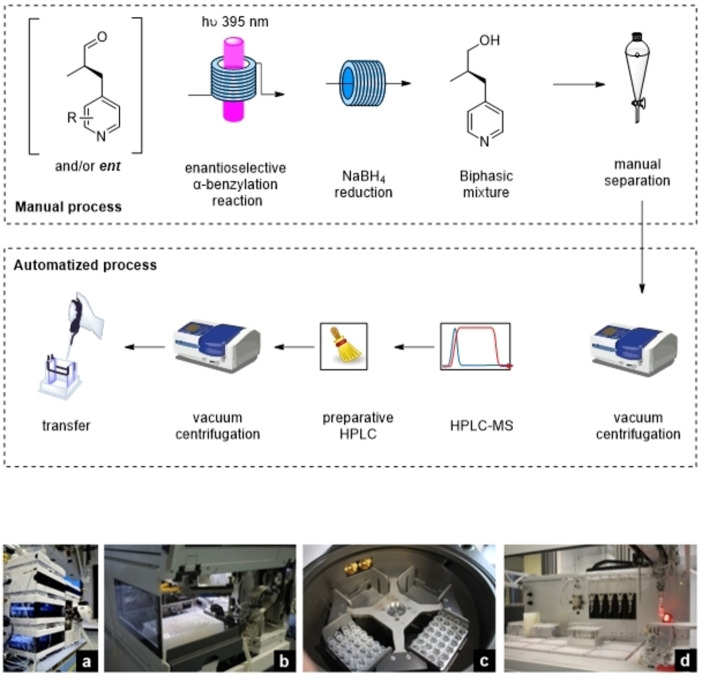
Semi automatized evaporation, purification, evaporation, transferring, weighing, after manual phase separation. (Photos left‐to‐right: a) HPLC‐MS, b) Pipetting and weighing robot, c) Vacuum centrifuge, d) analytical preparation sample.

In translating the reaction into continuous flow, preliminary considerations suggested that using HPLC tubing, with different diameters (0,25–1 mm), the irradiation efficiency should not be compromised by the complete absorption of photons. Based on the few examples of pilot‐plant kilogram‐scale continuous flow (tubular) photoreactors in industry,[^15^, ^16^] perfluoroalkoxyalkane (PFA) HPLC‐tubing were chosen for the water‐cooled photoreactor designed for cryogenic conditions. Screening under microfluidic and mesofluidic conditions have been performed to realize a up to 100x fold upscaling of the enantioselective reaction (Figure 2). Under microfluidic conditions, the reactions proceed around 20 times faster (∼60 min for high conversion) vs. batch conditions (24 h). Only the electron rich quinoline alcohol exhibits a considerably slower reaction kinetic, as previously described.^[12]^

**Figure 2:**
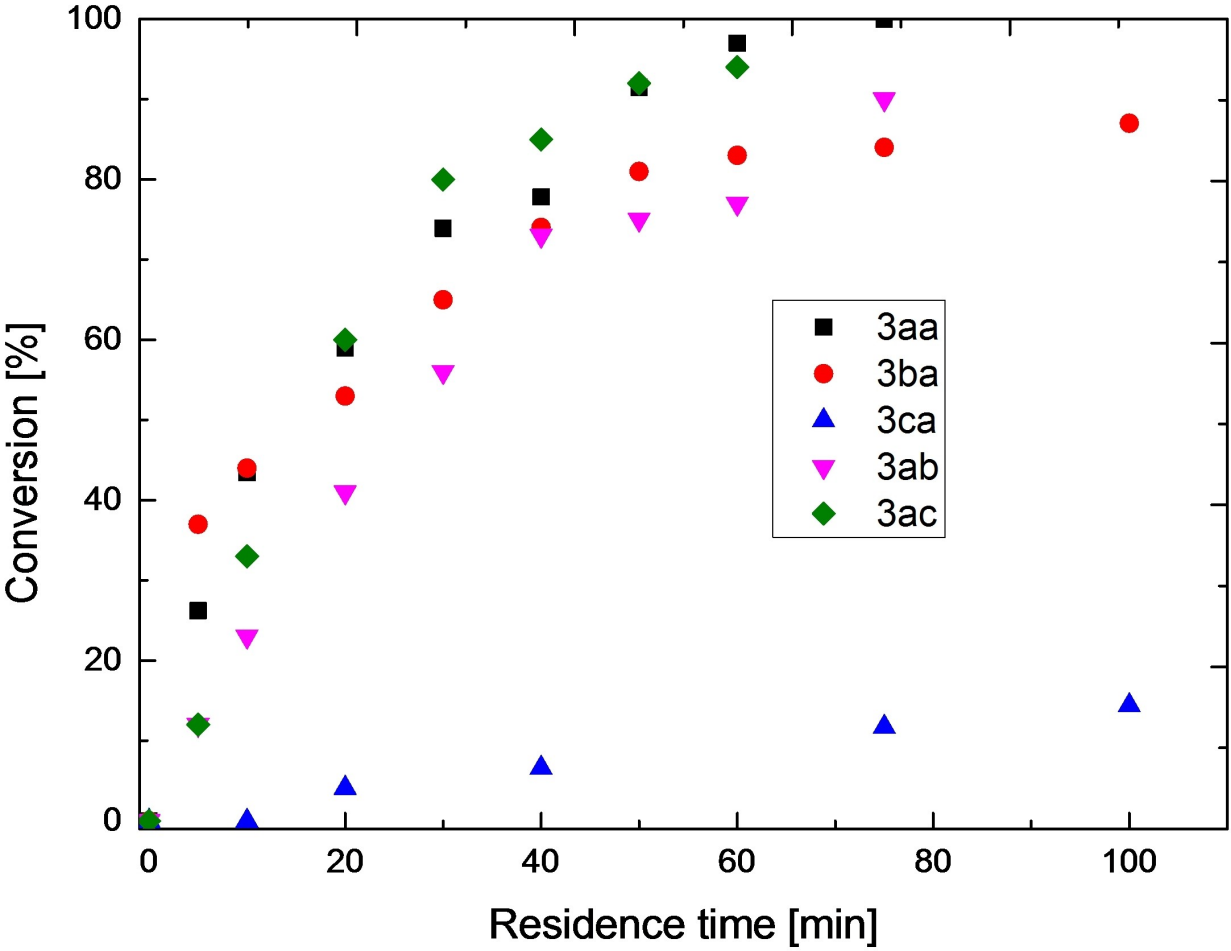
Conversion vs. residence time under microfluidic conditions.

Increasing the reaction time at the flatter end of the curve only leads to minimal increases in (theoretical) isolated yield. The goal of this work is to reach high productivities with still acceptable levels of conversion. This compromise is best estimated at the point where the slope of the conversion curve changes from steep to flat. In Figure 3 is illustrated the productivity curve of one selected example (**3 aa**).

**Figure 3:**
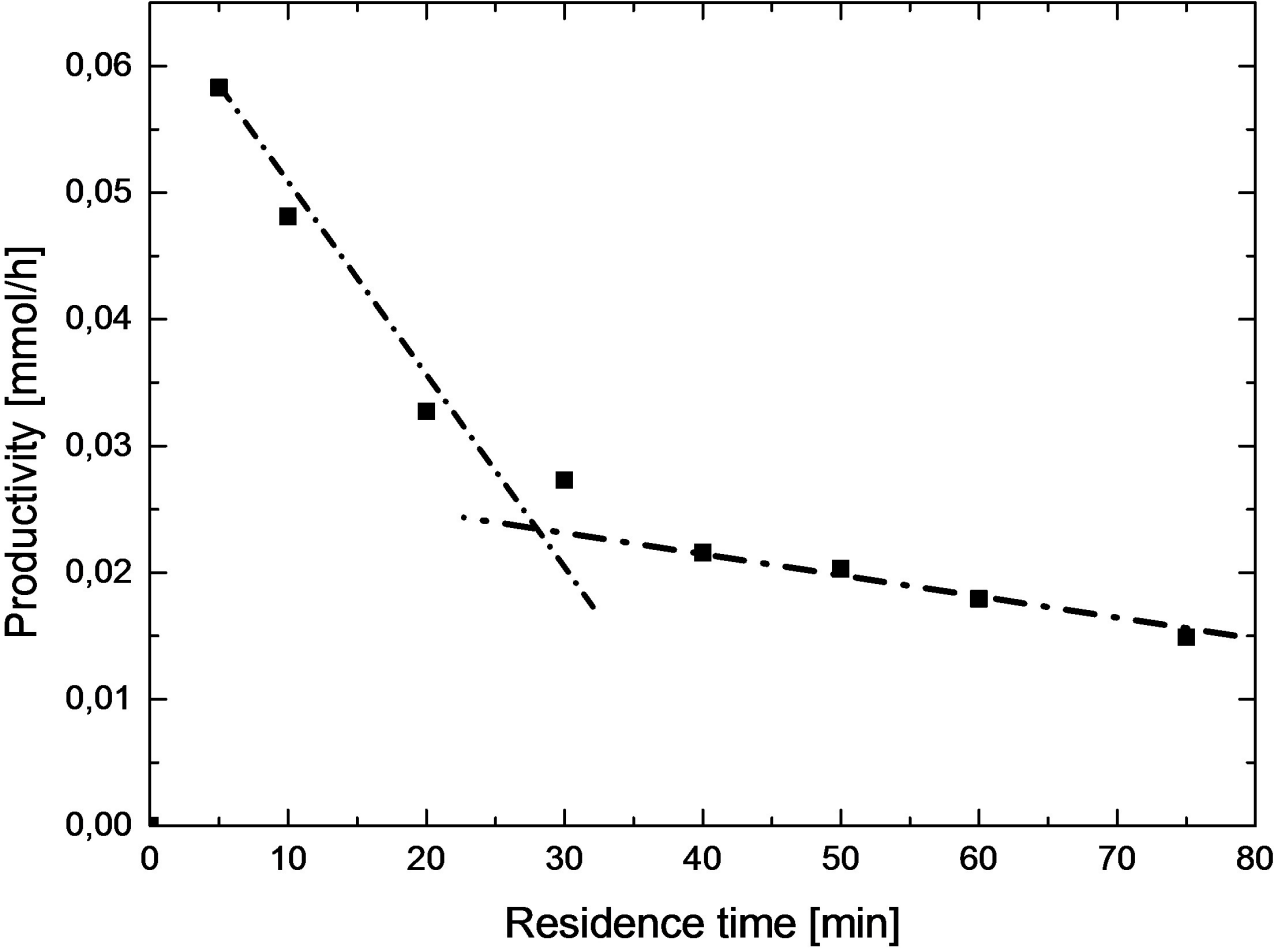
Illustration of the double regression strategy to determine the sweet spot for conversion of molecule **3 aa**.

This curve shows the initial steep slope followed by a steady decline until the point of flattening is reached. A double regression strategy was employed; the intersection represents the point where steep slope converts to flatter slope. For this specific example the optimal compromise was ∼30 min.

With the optimum compromise conditions at hand each molecule was synthesized by means of collecting the output of the reactor for the time required to get the same moles of product (assuming quantitative yield) as under batch conditions for optimal comparability. The outcome of those experiments is summarized in Table 2. Yields and enantioselectivities are comparable to batch conditions. For the quinoline scaffold **1 c** a stop‐flow experiment was undertaken, filling a 1 mL reactor with the reaction mixture and irradiating it for 24 h, as under batch conditions; by the increased surface, the isolated yield almost doubles (Table 1 entry 3, Table 2 entry 3).

**Table 2:** Results from the microfluidic experiments to generate values for benchmarking the reactor in terms of productivity.

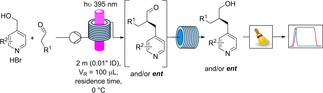				
Entry	Molecule	Residence Time [min]	ee [%]	Yield [%]^[b]^
1	**3 aa**	30	98	43
2	**3 ba**	35	99	55
3^[a]^	**3 ca**	1440	91	62
4	**3 ab**	40	94	57
5	**3 ac**	25	96	83

[a] Stop flow experiment, reaction time 24 h in a 1 mL Reactor. [b] isolated yield after chromatography corrected by conversion. Reaction mixture was collected until 250 μmol of pyridyl alcohol (limiting reagent) were fed into the reactor.

Scaling up by a factor of ten was undertaken next. By doubling the internal diameter and roughly doubling the reactor length, a reactor with an internal volume of 1 mL was built and tested under the conditions of the microfluidic screening (Table 3). The overall efficiency drops slightly when comparing to microfluidic conditions; conducting the reaction at room temperature instead of 0 °C (entries 3 and 7, Table 3) the yield of **3 aa** increased, while it decreased for compound **3 ac**.

**Table 3:** Results from mesofluidic (1 mL) experiments to generate values for benchmarking the reactor in terms of productivity.

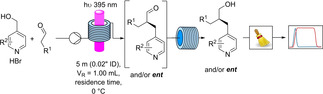				
Entry^[a]^	Molecule	Residence Time [min]	ee [%]	Yield [%]^[b]^
1	**3 aa**	30	98	28
2	**3 aa**	60	98	47
3^[a]^	**3 aa**	60	96	62
4	**3 ac**	25	96	31
5	**3ac^b^**	50	95	64
6	**3 ac**	75	95	77
7^a^	**3 ac**	50	93	31

[a] Experiment conducted at room temperature. [b] isolated yield after chromatography (not corrected). Reaction mixture was collected until 250 μmol of pyridyl alcohol (limiting reagent) were fed into the reactor.

With longer residence times, higher isolated yields were observed, with an improved productivity for compound **3 ac** (entries 4 and 5, Table 3). Further increase of the residence time by another 50 %, did not lead to significant improvements.

Again, for the next factor 10 upscaling step, internal diameter and tubing length were roughly doubled to give a reactor with a volume of 10 mL. As the photoredox catalyst is quite expensive (∼$120/50 mg), the 10 mL reactor was first tested under segmented flow conditions: an immiscible solvent (*n*‐heptane) is pumped after the reaction solution. This procedure drastically reduces overall cost and the amount of “waste” that is generated, while only collecting the same moles as under batch conditions. As it is illustrated in Table 4, with the 10 mL reactor a little reduction of efficiency was detected (35 % yield vs. 47 % with the 1 mL reactor). When the residence time was doubled the isolated yield increased with it although subpar (entry 3, Table 4). Using propionaldehyde, the segmented flow split into a Taylor‐flow regime, meaning droplets of reaction mixture were followed by larger droplets of heptane. Also, fluorescence of the heptane droplets started to occur, indicating photoredox catalyst was present. This observation can explain the less efficient overall process for compound **3 ac** (entries 4–6, Table 4).

**Table 4:** Results from segmented flow (10 mL) experiments.

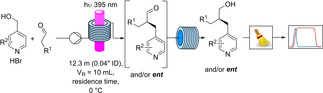				
Entry	Molecule	Residence Time [min]	ee [%]	Yield [%]^[b]^
1^[a]^	**3 aa**	30	96	21
2^[a]^	**3 aa**	60	97	35
3^[a]^	**3 aa**	120	97	48
4^[a]^	**3 ac**	25	96	8
5^[a]^	**3ac^a^**	50	97	25
6^[a]^	**3 ac**	75	95	38

[a] segmented flow conditions used. [b] isolated yield after chromatography. Reaction mixture was collected until 250 μmol of pyridyl alcohol (limiting reagent) were fed into the reactor.

The findings from the segmented flow experiments were directly translated into a standard continuous flow experiment, where comparable yields were obtained (38 % vs. 35 % in segmented flow), with excellent enantioselectivity (92 % e.e.). Generally, when no flow splitting is occurring, the segmented flow method can thus be used to save reagents and still get insight into the reactor efficiency and productivity.

The isolated yields of most of the continuous flow experiments are lower than yields achieved by MacMillan et al. in the original publication that inspired this work.^[12]^ Several contributing factors may be responsible for lowering the yield considerably: a) photon flux considerations show that the organocatalytic cycle becomes rate limiting. Enamine formation was determined to be slow, iminium‐ion hydrolysis was estimated to be even slower,^[12]^ and probably too many benzylic radicals get generated. Under batch conditions this effect gets counter‐acted by a predominantly “dark” reactor; b) side reactions previously described by MacMillan et al. became more predominant (reduction of benzylic radical or recombination),^[12]^ leading to poor overall selectivity.

One way to overcome this limitation is by the construction of a “darker”, more optimized reactor with a better balance of radical generation and organo‐catalytic cycle.

As previously discussed, a tubular continuous flow reactor often exhibits an upwards of two orders of magnitude increased surface‐to‐volume ratio making them perfectly suited for photoredox chemistry. The final comparison and judgement of productivities of the different types of reactors are summarized in Table 5, using a similar method to determine productivity as already established by Zeitler et al.^[9]^ Under microfluidic conditions (low reactor volumes) the productivity is almost tripled. Every factor 10 upscaling later was seamlessly translated into a factor 10 increase of productivity. The 10 mL mesofluidic reactor can provide an increase of productivity by almost 18000 %, with no loss of stereochemical efficiency, affording the product always in >95 % e.e.

**Table 5:** Numerical comparison of productivities of different reactor types and of normalized relative factors.

Entry	Method	Productivity^[a]^ [mmol/h]	Rel. Factor	STY^[b]^ [mmol/mL*h]
**1**	Batch	4.0×10^−3^	1	3.7×10^−3^
**2**	Microfluidic	1.2×10^−2^	2.8	1.2×10^−1^
**3**	1 mL Meso	9.4×10^−2^	22	9.6×10^−2^
**4**	10 mL Meso	7.6×10^−1^	177	7.8×10^−2^

[a] Productivity: moles of product (calculated from isolated yield) divided by the collection time required to collect the product obtained by the reaction of 250 μmol of alcohol; [b] Space‐time‐yield: moles of product in reactor, divided by residence time and reactor volume (for details on calculations please see the Supporting Information).

This seamless up‐scaling was further evidenced when considering the space‐time‐yield (STY), an excellent metric to compare reactors at different volumes. From batch to microfluidic, as indicated in Table 5, a sharp increase in STY was observed (x33). Every factor 10 upscaling only resulted in a decrease of STY by roughly 20 %, indicating up‐scaling by increasing length and diameter of the tubing worked effortlessly. Based on photon‐flux considerations and transmission behaviour, even at the 10 mL meso‐fluidic reactor with 1 mm of diameter there are still plenty of photons available, leading to a “bright” reactor. Obviously, due to the negative exponential attenuation of photons, at some point it is not sensical anymore to increase the diameter of the tubing. Therefore, industrially sized continuous‐flow (tubular) reactor often deal with tubing in length upwards of hundreds of meters.[^15^, ^16^]

Having established an efficient, highly enantioselective in‐flow protocol for the α‐alkylation of aldehydes, we decided to apply it in a fully telescoped continuous process. In Scheme 2 is shown a chiral patented API^[17]^ exhibiting the 4‐methylpyridine scaffold, a privileged motif in several APIs.[^12^, ^18^] The compound inhibits cycline dependent kinase 9 at nanomolar concentration making it a potent API for a potential cancer drug.^[19]^ The published synthesis consists of 6 linear steps (6 % overall yield), with the last step being a low yielding Buchwald‐Hartwig type aromatic amidation (Scheme 2). To the best of our knowledge, no previous article was published reporting a stereoselective photoredox organocatalytic continuous flow reaction in a telescoped process for an API.

**Scheme 2:**
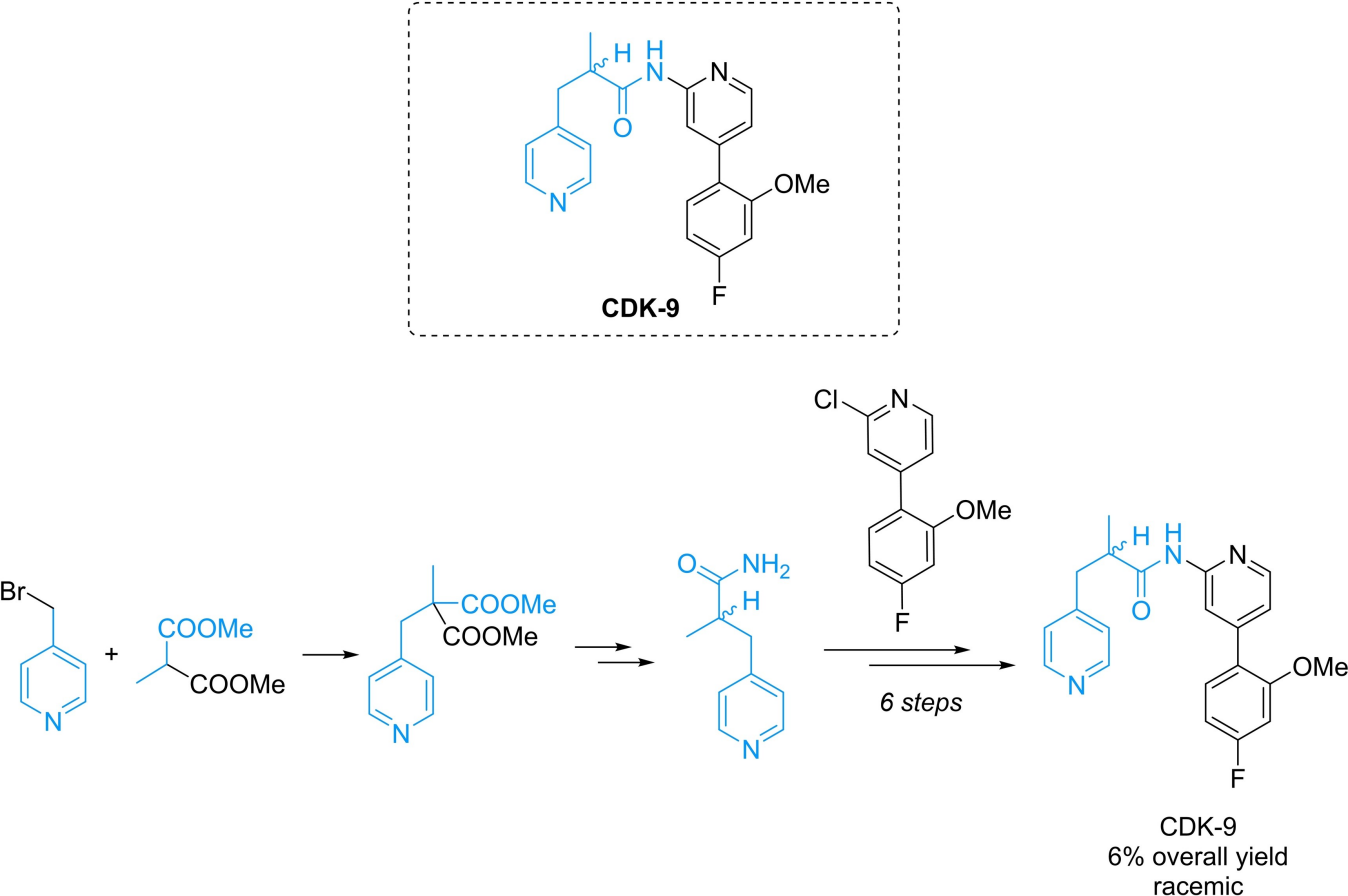
Synthesis of a patented API for a potential cancer drug.

A first approach involved the oxidation of the aldehyde to the carboxylic acid, to prevent the critical epimerization of the aldehyde. The carboxylic acid was to be reacted in an amide coupling with the aminopyridine building block. Two distinct strategies were tried unsuccessfully, as depicted in Scheme 3. As first attempt, the whole reaction mixture was pumped into a continuous stirred tank reactor (CSTR) where it was reacted with a sodium chlorite solution for the mild and selective Pinnick oxidation, which is known for respecting the integrity of stereogenic centers. While the oxidation worked with high efficiency, the subsequent continuous phase separation with a membrane separator did not proceed satisfactorily, most likely due to the zwitterionic nature of the carboxylic acid.

**Scheme 3:**
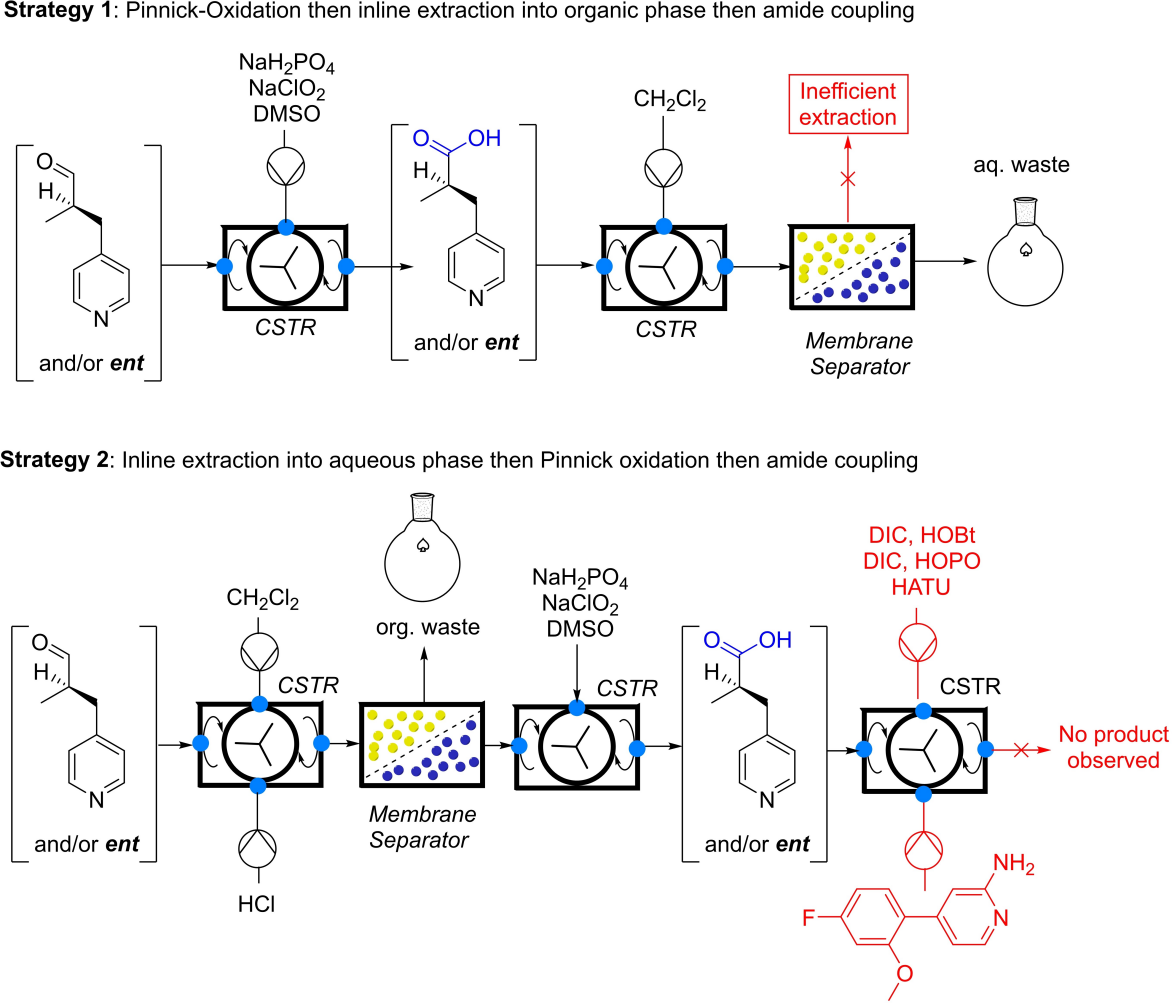
Attempted oxidation/amide coupling strategies.

In the second approach, the aldehyde was first extracted with high efficiency using the same continuous membrane separator into the aqueous phase by pumping in an acidic buffer sodium phosphate solution. This extraction step significantly cleaned the reaction mixture as most of the reagents remained in the organic waste. The aqueous phase was then used *in continuo* to perform the Pinnick oxidation; however, all attempts to make an aqueous amide coupling failed.^[20]^

As alternative strategy, we investigated the direct oxidative amidation of aldehydes with aminopyridines catalysed by Cu(I) in combination with *tert*‐butyl hydroperoxide (Scheme 4).^[21]^

**Scheme 4:**
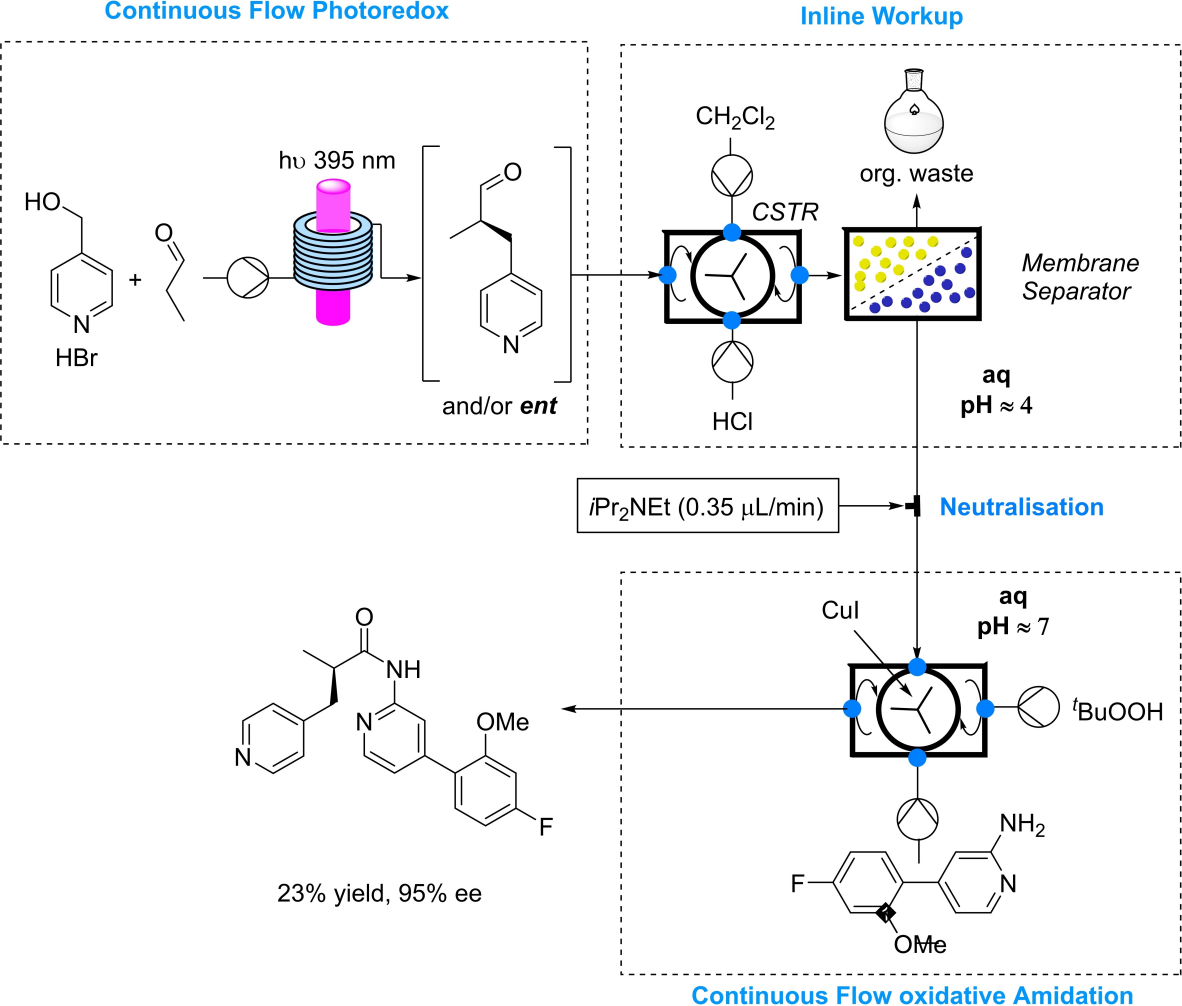
Fully telescoped, fully continuous synthesis of a privileged API.

After running some successful test reactions under batch conditions, this methodology was incorporated into the continuous flow process, and finally the fully continuous, fully telescoped process to synthesize a complex API was realized. The final process consists of 4 units of operation: asymmetric benzylation under continuous flow, inline continuous work‐up, neutralisation and the final oxidative amidation (Scheme 4).

The final product was obtained in 23 % overall yield and 95 % e.e. Shaving off 4 linear steps the overall yield was increased by a factor of 4, showcasing the powerful methodology of the stereoselective benzylation.

This work represents a further demonstration of how photoredox chemistry could be efficiently performed under continuous flow conditions. Comparing with the in‐batch process, the overall efficiency of tubular reactors is more than two order of magnitude higher. Getting insight from photophysical measurements is critical to know how the upscaling strategy can be developed. Having an efficient continuous flow photoredox reactor at hand allowed for the incorporation as one unit of operation into a fully telescoped, fully continuous highly enantioselective synthesis of a chiral privileged API.

## Conflict of interest

The authors declare no conflict of interest.

## Supporting information

As a service to our authors and readers, this journal provides supporting information supplied by the authors. Such materials are peer reviewed and may be re‐organized for online delivery, but are not copy‐edited or typeset. Technical support issues arising from supporting information (other than missing files) should be addressed to the authors.

Supporting Information

Supporting Information

## Data Availability

The data that support the findings of this study are available from the corresponding author upon reasonable request.
