# Supplementary material for: Enantioselective Organophotocatalytic Telescoped Synthesis of a Chiral Privileged Active Pharmaceutical Ingredient
Source: Chemistry. 2022 May 4;28(33):e202200164. doi: 10.1002/chem.202200164 (PMC9325444; doi:10.1002/chem.202200164)
Supplement: Supplementary file 2 — Supporting Information [file CHEM-28-0-s002.pdf]

Chemistry–A European Journal

Supporting Information

Enantioselective Organophotocatalytic Telescoped Synthesis of a Chiral Privileged Active Pharmaceutical Ingredient

Fabian Herbrik, Miguel Sanz, Alessandra Puglisi, Sergio Rossi, and Maurizio Benaglia*

Table of Contents

Table of Contents 1
General Description of Reagents and Methods 2
Experimental Procedures 3
Description of Equipment 3
Continuous Flow Coil Reactor Specifications 5
Photochemical and Photophysical Considerations for Scalability 6
General Procedure for Batch Reactions – Benchmarking the Photoreactor 7
Table S2. Results from the batch experiments 8
Purification and Characterization of Compounds 3aa, 3ba, 3ca, 3ab, 3ac 9
General Procedure for Microfluidic Reaction (screening) 11
General Procedure for Microfluidic Reactions (Synthesis) 13
Calculation of Productivity and Space-Time-Yield 17
Telescoping: Inline oxidation to the carboxylic acid then extraction 18
Telescoping: First extraction then oxidation then aqueous amide coupling 19
Fully Telescoped fully continuous Synthesis of the API 20
Appendix ^{1}H-NMR-Spectra 22
uHPLC-Traces coupled with APCI/ESI-MS-spectra 28
Racemic Samples in Chiral HPLC 33
Chiral Samples 38
Table S2 38
Table S3 43
Table 4 48
Table S5 55

General Description of Reagents and Methods

All reactions were carried out under a positive pressure of nitrogen (5 cm of mercury, or with a spring loadedsilicon oil bubbler set to 100 mbar). Oxygen being an effective quencher of the photoredox-catalyst it was taken special measures to avoid oxygen such as bubbling nitrogen through the stock solution for at least 30 min. As most reactions involved water as (co)-solvent no special care was taken to avoid water residues. If not otherwise stated reagents, solvents and such were used without further purifications. *N,N*-dimethylacetamide was degassed and stored under nitrogen. HPLC-grade water was degassed and stored under nitrogen. Octanal and propanal were flushed through a plug of basic aluminium oxide and then distilled under nitrogen and stored under nitrogen. Propanal when used in the reaction was not added before degassing the stock-solutions but was rather degassed separately each time and then was added to the stock-solutions due to its volatility. 3-phenyl-1-propanal (hydrocinnamaldehyde) was distilled under reduced pressure and then stored under nitrogen. 2,6-lutidine was first refluxed and then distilled over calcium hydride and stored under nitrogen. The benzylic alcohol radical sources were synthesized according to literature described procedures.[1] The chiral imidazolidinone catalysts (*R*- and *S*-enantiomer) were purchased from Sigma-Aldrich and used as received. Ir[p-^{t}Bu-ppy]$_3$ photoredox-catalyst was purchased from Sigma-Aldrich and used as received.

Reactions were monitored by thin layer chromatography (TLC) on Macherey-Nagel pre-coated silica gel plates (0.25 mm) and visualized by UV light. Flash chromatography was performed on Merck silica gel (60, particle size: 0.040–0.063 mm). ^{1}H NMR ^{13}C NMR and ^{19}F NMR spectra were recorded on Bruker Avance III HD 600, Bruker Avance-400, Bruker Avance-300 or Bruker Avance-250 spectrometer in $CDCl_3$ as solvents at room temperature. Chemical shifts for protons are reported using residual solvent protons (^{1}H NMR: δ = 7.26 ppm for $CDCl_3$) as internal standard. Carbon spectra were referenced to the shift of the ^{13}C signal of $CDCl_3$ (δ = 77.0 ppm). The following abbreviations are used to indicate the multiplicity in NMR spectra: s - singlet; d - doublet; t - triplet; q - quartet; dd - double doublet; ddd – doublet of doublet of doublets; dt - doublet of triplets; m - multiplet; quint – quintuplet; sext - sextuplet sept - septet; br - broad signal; dq – doublet of quartets.

High resolution mass spectra (HRMS) were acquired using a Bruker solariX XR Fourier transform ion cyclotron resonance mass spectrometer (Bruker Daltonik GmbH, Bremen, Germany) equipped with a 7 T refrigerated actively-shielded superconducting magnet. The samples were ionized in positive ion mode using a MALDI or ESI ionization sources.

Automatic weighing and transferring of liquids was made with a Zinssler Analytics custom robot “Calli”.

Samples were evaporated in a parallel fashion by employing a Genevac HT-4X vacuum centrifuge Series II System.

Preparative HPLC-MS was conducted on a Agilent 1260 Infinity Series (Autosampler, Fraction Collector, DAD, Pumps, Check valves, all while coupled to a Agilent 6120 LC-MS Quadrupole mass-spectrometer. MS-traces were generated in positive/negative switching mode and ESI/APCI as ionization method was used in tandem.

uHPLC-MS (ultrahigh performance) was conducted on a Agilent 1260 Infinity Series (Autosampler, Pump was 1290 Infinity Series) all while coupled to an Agilent 6120 LC-MS Quadrupole mass-spectrometer. MS-traces were generated in positive/negative switching mode and ESI/APCI as ionization method was used in tandem.

Chiral HPLC was measured on a Agilent 1100 Series (DAD, Autosampler, Pumps). The respective chiral stationary phase is indicated in the characterization part.

Experimental Procedures

Description of Equipment

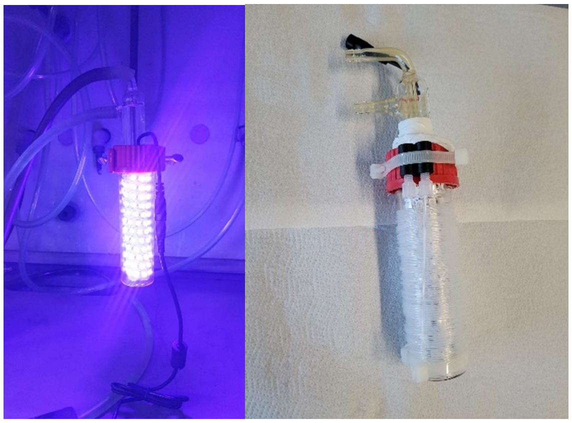

Figure 1: Photograph of the ignited watercooled high intensity LED photoreactor for cryogenic continuous flow operations. Photograph of the coil-reactor (12.33 m, 0.04 in ID Perfluoroalkoxyalkane (PFA) HPLC-Tubing that is wrapped around the aforementioned photoreactor.

A versatile LED-based photoreactor for continuous flow reactions under *cryogenic conditions* is unprecedented in Literature. The goal is to build a photoreactor for such conditions using only readily available and low-cost equipment that can be found in any organic chemistry laboratory. The herein presented Photoreactor can be immersed in any cryobath for temperature regulation as the LEDs are *hermetically sealed* inside a Pyrex glass tube to avoid glacier formation, which can obstruct efficient irradiation due to the high albedo of water ice that would form from atmospheric moisture. The heat generation which tends to burn high power LEDs under sealed conditions is counteracted by wrapping the LED-Strips around a central sublimator glass-piece which is water cooled. With this innovative reactor design, it is possible to run a continuous flow photoreaction at any temperature by simply immersing the photoreactor and coil reactor couple in a temperature-controlled liquid (Ice-mixtures, dry-ice mixtures, cryostat-baths, oil-baths). The here presented Reactor performed for over 300 h and is still fully operational.

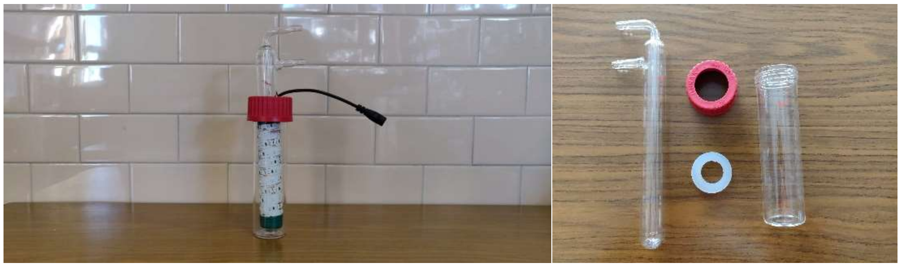

Figure 2:Photographs of the assembled and dissembled water-cooled photoreactor for cryogenic continuous flow operations

Construction of the Photoreactor: The central sublimator glass-piece is first wrapped to the desired length with heavy duty aluminium foil to generate a socket for the LED-strip that possesses high heat conductive properties. Around this first layer is then coiled and glued (doublesided adhesive tape) the LED-strip which is further secured in place at the top and bottom with electric isolating tape. The cable is guided through the silicon rubber seal by puncturing it. The final reactor is then assembled as presented in Figure 2. Video of the assembly can be found separately uploaded under supporting files.

Led-Specifications: Aftertech® 24v 24w UV 1m Strip 120 LEDs SMD5050 ultraviolet (395 nm), with a self-adhesive tape that holds the strip light safely and securely to the photoreactor support. The LEDs wavelength emission profile together with their specific light intensity (expressed as mW cm^{-2}) have been determined before their use. In particular, the spectrum has been obtained by using a compact CCD spectrometer (model CCS200/M) connected to a multimode optical fiber, purchased from Thorlabs. Ultraviolet LEDs are characterized by an almost monochromatic emission profile (with a full width at half-maximum intensity of ca. 10-20 nm) showing a maximum of intensity located at ca. 395 nm (Graphic 1). The light power intensity was thus checked using a Thorlabs PM200 power meter equipped with a S130VC power head with a Si detector. The measured light intensities, though slightly decreasing by moving the maximum of LEDs emission towards longer wavelenghts, was I = 70.94 mW cm^{-2} (std 0.0034).

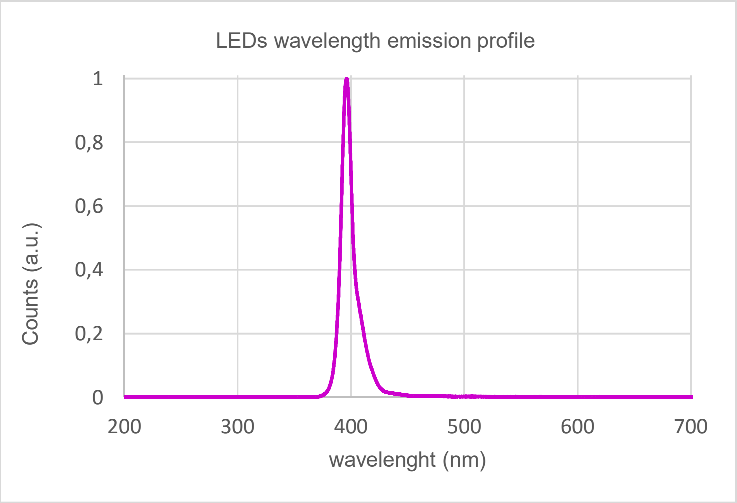

Graphic 1:UV- LEDs wavelength emission profile

Continuous Flow Coil Reactor Specifications

Table S1. Specifications for the reactors used in the work. All three coil reactors are made from standard HPLC-Tubing – Material PFA

Entry	Reactor	Reactor Volume	Internal Diameter (Outer Diameter) [in]	Reactor Length [m]	Illuminated Surface Area [cm^2]
**1**	Microwave-Vial	1 mL	--	---	1
**2**	Micro-	100 μL	0.01	2.00	8
**3**	Small Meso-	1 mL	0.02	5.00	40
**4**	Meso-	10 mL	0.04	12.33	200

All fluidic connections were made by ¼-28-bore fingertight ferules and adapters (connectors, Y- and T-type) and were purchased together with the HPLC-tubing. at Cole-Parmer.

Photochemical and Photophysical Considerations for Scalability

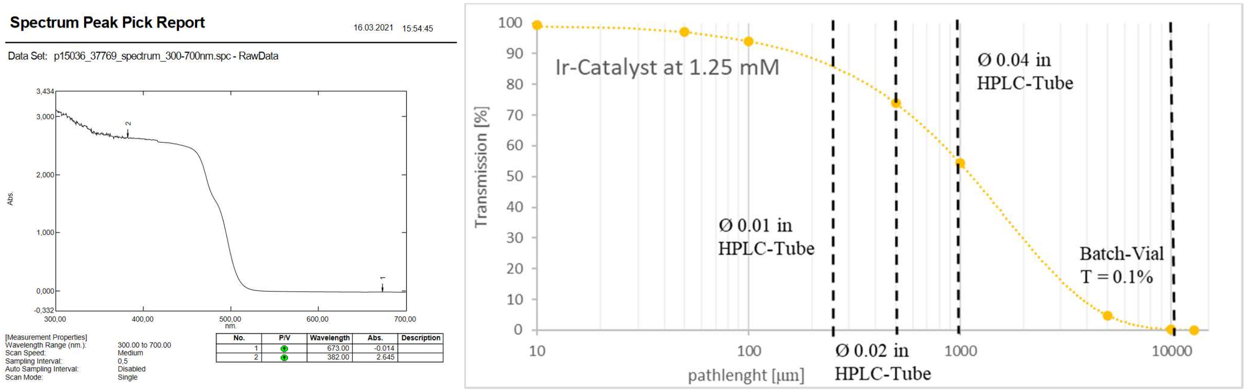

Figure 3: Absorption spectrum of the reaction solution, deaerated measured in a standard single-use cuvette. Calculated transmission curve (log-scale) based on the absorption spectrum. The intersection between the curve and the dashed lines represents the transmission that is to be expected at the respective reactor internal diameter.

Measuring the absorption spectrum of the reactive solution indicates the most suitable reactor type and size for scaling photoredox chemical processes. In the present example the photoredox solution absorbs light with an absorption of A = 2.63 au at 395 nm of UV-radiation. Based on this, it is generally sensible to quickly plot the transmission curve of aforementioned solution based on the following formula:

$$T\% = 10^{-A*ID(reactor)}\ , A: measured\ absorption, ID(reactor): internal\ diameter\ of\ reactor$$

Dealing with a negative exponential attenuation it is sensible to scale the x-axis logarithmically to visualize the different internal diameters of the reactors inside the curve. Generally, a good indicator is the transmission cut-off:

- at 0.1% for reactions with a high quantum yield
- at 1% for reactions with a low quantum yield

Optimally choosing the internal diameter of the reactor at exactly those transmission cut-offs allows for the most productive process. In practice self-made coil reactors have to be chosen on an availability basis.

In the present example all the employed continuous flow reactors are sized that there are still copious amounts of irradiation left and that several upscaling steps could still be undertaken.

General Procedure for Batch Reactions – Benchmarking the Photoreactor

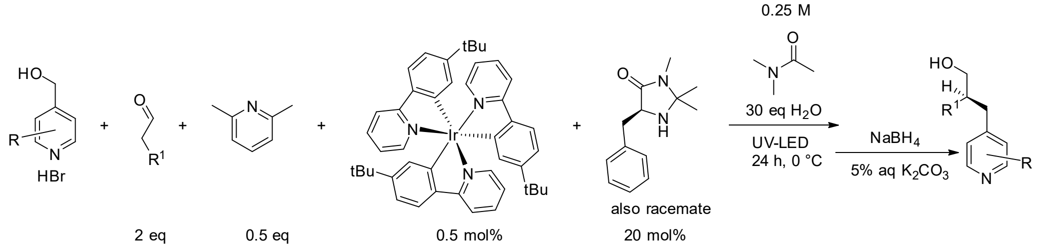

Benzylic alcohol radical source (250 µmol, 1.00 eq), (5*S*)-(-)-2,2,3-Trimethyl-5-benzyl-4-imidazolidinon -monohydrochloride 13.0 mg (50 µmol, 0.20 eq), Ir[4-tBu-ppy]$_3$ 1.00 mg (12.5 µmol, 0.005 eq) were placed inside MW-vial, sealed with a aluminium cap with rubber septum and a N_2-atmosphere was applied. *N*,*N*-Dimethylacetamide (degassed, stored under N_2) 1 mL Distilled water (degassed, stored under N_2) 134µL, 2,6-Lutidine (degassed, stored under N_2) 15.0 µL, (125 µmol, 0.50 eq), aldehyde (degassed, stored under N_2) (500 µmol, 2.00 eq) were cannulated under strict N_2-atmosphere. The mixture was degassed by bubbling N_2 for 30 minutes. The vial was placed directly adjacent to the 395 nm high intensity LED photoreactor and was irradiated for 24 h maintaining 0°C. The reaction was quenched with 10 eq $NaBH_4$ in 5% aqueous K_2CO_3-Solution at 0°C and left stirring overnight. 5 mL of dichloromethane were added to the mixture and the aqueous phase was extracted two additional times with 5 mL of dichloromethane. The combined organic phases were dried over magnesium sulfate and the volatiles evaporated in vacuo (HV-rotavap). The resulting remains were purified using preparative RP18-Silica and a gradient of water/acetonitrile with 0.5mM NH_4HCO_3 using an in-house generated protocolfor the semi-automated purification, evaporation. For this the workflow is illustrated in figure 4. After the automatic chromatographic purification of the compounds the solvent was evaporated in a parallelized fashion inside a vacuum centrifuge. The tube positions with product containing fractions were submitted automatically to the transfer robot. The robot then backfills the tubes with acetonitrile to redissolve and transfers this to barcode-vials which were weighed by robot before filling. The bar-code vials containing the solutions of the product were then evaporated again in a parallel fashion and the robot after weighing the vials again comminucates the final yield.

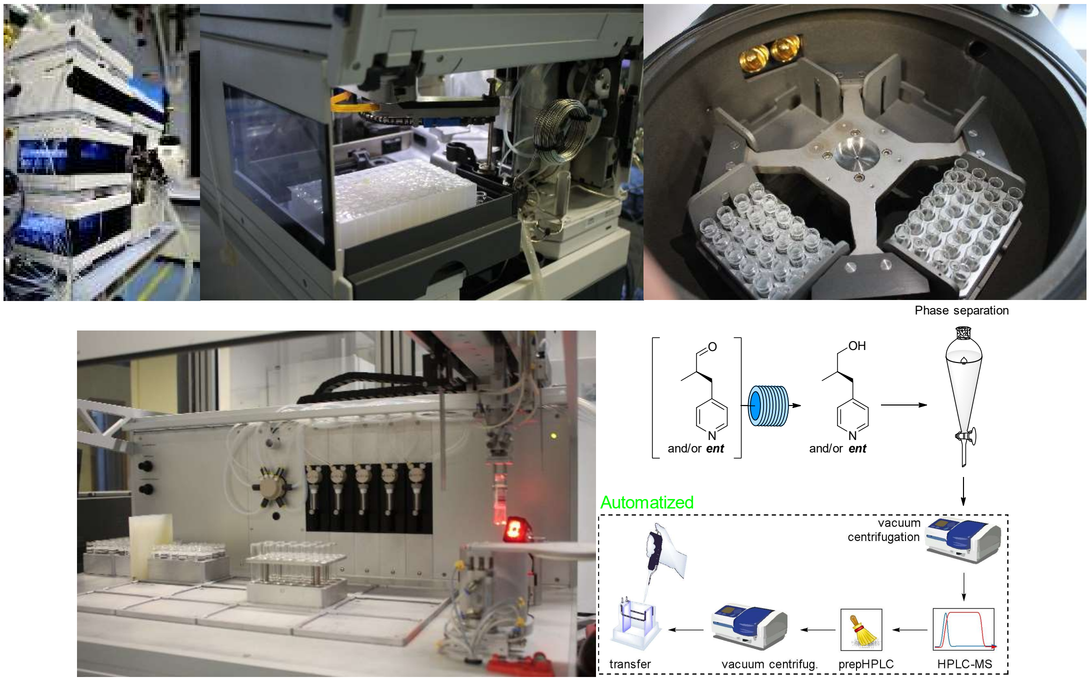

Figure 4. Semi automatized evaporation, purification, evapo-ration, transferring, weighing, after manual phase separation. (photos left-to-right: Pipetting and weighing robot, Vacuum centrifuge, analytical/prep. HPLC-MS)

After vacuum centrifugation the compounds were isolated as stated in Table S2. The overall procedure was repeated to generate the racemic samples by carefully weighing the *R*- and *S*-enantiomer of the chiral imidazolidinone catalyst in equal ratio.

Table S2. Results from the batch experiments

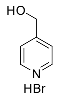
1a

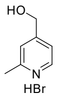
1b

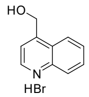
1c

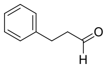
2a

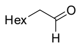
2b

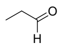
2c

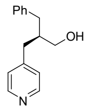
3aa

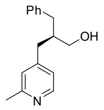
3ba

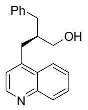
3ca

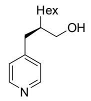
3ab

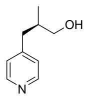
3ac

Entry[a]	Molecule	ee [%]	Yield [mg], {%}
1	3aa	96	24.3 , 43%
2	3ba	97	19,1 , 32%
3	3ca	96	14.6 , 32%
4	3ab	94	17.0 , 31%
5	3ac	96	16.8 , 44%

Purification and Characterization of Compounds 3aa, 3ba, 3ca, 3ab, 3ac

Purification	Crude after workup dissolved in 1 ml of MeOH/MeCN 1:1 and purified by prep-HPLC.<br>Column: XBridge BEH Prep C18 5 μm, 19 mm X 150 mm<br>Mobile phase: Acetonitrile / Water+5mM NH_4HCO_3<br>Flow rate: 32 ml/min<br>Gradient: 0-0.5 min 10:90<br>0.51-12,5 min 80:20<br>12,5-14,5 min 98:2	
^{1}H NMR	(300 MHz, Chloroform-*d*) δ 8.47 – 8.35 (m, 2H), 7.31 – 7.01 (m, 7H), 2.68 (ddd, *J* = 13.7, 7.9, 2.5 Hz, 2H), 2.56 (dd, *J* = 13.6, 6.6 Hz, 2H), 2.09 (ddt, *J* = 7.9, 6.4, 1.4 Hz, 1H), 1.82 (s, 2H).	
Chiral HPLC	Chiralpak IA, isocratic 9:1 nHex/EtOH eluent<br>Retention time 17.2 min (minor), Retention time 12.6 min (major),	
MS (ESI+APCI)	m/z = 228.2 [M+H$^+$]	

Purification	Crude after workup dissolved in 1 ml of MeOH/MeCN 1:1 and purified by prep-HPLC.<br>Column: XBridge BEH Prep C18 5 μm, 19 mm X 150 mm<br>Mobile phase: Acetonitrile / Water+5mM NH_4HCO_3<br>Flow rate: 32 ml/min<br>Gradient:<br>0-0.5 min 14:86<br>0.51-12,5 min 65:35<br>12,5-14,5 min 98:2	
^{1}H NMR	(300 MHz, Chloroform-*d*) δ 8.30 (d, *J* = 5.1 Hz, 1H), 7.29 – 7.19 (m, 2H), 7.19 – 7.07 (m, 3H), 6.91 (s, 1H), 3.42 (d, *J* = 4.9 Hz, 2H), 2.72 – 2.61 (m, 2H), 2.61 – 2.52 (m, 2H), 2.45 (s, 3H), 2.15 – 2.00 (m, 1H), 1.69 (s, 2H).	
Chiral HPLC	Lux Amylose 1, isocratic 95:5 nHex/EtOH eluent<br>Retention time 19.2 min (minor), Retention time 24.0 min (major),	
MS (ESI+APCI)	m/z = 242.2 [M+H$^+$]	

Purification	Crude after workup were dissolved in 1,2 ml of MeOH/MeCN 1:1 and purified by prep HPLC.<br>Column: XBridge BEH Prep C18 5 μm, 19 mm X 150 mm<br>Mobile phase: Acetonitrile / Water+5mM NH4HCO3<br>Flow rate: 32 ml/min<br>Gradient:<br>0-0.5 min 11:89<br>0.51-10,5 min82:18<br>10,5-12,5 min 98:2	
^{1}H NMR	(300 MHz, Chloroform-*d*) δ 8.68 (d, *J* = 4.4 Hz, 1H), 8.02 (dd, *J* = 8.5, 1.3 Hz, 1H), 7.73 (dd, *J* = 8.6, 1.4 Hz, 1H), 7.60 (ddd, *J* = 8.4, 6.9, 1.4 Hz, 1H), 7.39 (ddd, *J* = 8.3, 6.8, 1.3 Hz, 1H), 7.29 – 7.06 (m, 7H), 3.46 (d, *J* = 4.7 Hz, 2H), 3.11 (dd, *J* = 13.7, 8.4 Hz, 1H), 2.99 (dd, *J* = 13.6, 5.9 Hz, 1H), 2.80 (dd, *J* = 13.6, 7.5 Hz, 1H), 2.63 (dd, *J* = 13.5, 7.1 Hz, 1H), 2.26 – 2.12 (m, 1H), 1.73 (s, 2H).	
Chiral HPLC	Lux Amylose 1, isocratic 95:5 nHex/EtOH eluent<br>Retention time 36.9 min (minor), Retention time 39.4 min (major),	
MS (ESI+APCI)	m/z = 278.2 [M+H$^+$]	

Purification	Crude after workup dissolved in 1 ml of MeOH/MeCN 1:1 and purified by prep-HPLC.<br>Column: XBridge BEH Prep C18 5 µm, 19 mm X 150 mm<br>Mobile phase: Acetonitrile / Water+5mM NH4HCO3<br>Flow rate: 32 ml/min<br>Gradient:<br>0-0.5 min 20:80<br>0.51-12,5 min 76:24<br>12,5-14,5 min 98:2	
^{1}H NMR	(300 MHz, Chloroform-*d*) δ 8.57 – 8.37 (m, 2H), 7.17 – 7.03 (m, 2H), 3.52 (dd, *J* = 5.3, 2.9 Hz, 2H), 2.72 (dd, *J* = 13.6, 7.4 Hz, 1H), 2.60 (d, *J* = 6.9 Hz, 1H), 1.81 (ddd, *J* = 7.1, 5.2, 1.8 Hz, 1H), 1.69 (s, 1H), 1.48 – 1.15 (m, 11H), 0.94 – 0.77 (m, 3H).	
Chiral HPLC	Chiralpak AS-3, isocratic 98:2 nHex/IPA eluent<br>Retention time 35.5 min (minor), Retention time 39 min (major),	
MS (ESI+APCI)	m/z = 222.2 [M+H$^+$]	

Purification	Crude after workup dissolved in 1 ml of MeOH/MeCN 1:1 and purified by prep-HPLC.<br>Column: XBridge BEH Prep C18 5 µm, 19 mm X 150 mm<br>Mobile phase: Acetonitrile / Water+5mM NH4HCO3<br>Flow rate: 32 ml/min<br>Gradient:<br>0-0.5 min 10:90<br>0.51-12,5 min 56:44<br>12,5-14,5 min 98:2	
^{1}H NMR	(300 MHz, Chloroform-*d*) δ 8.49 – 8.40 (m, 2H), 7.14 – 7.07 (m, 2H), 3.49 (dd, *J* = 6.0, 0.9 Hz, 2H), 2.82 (dd, *J* = 13.4, 5.9 Hz, 1H), 2.38 (dd, *J* = 13.4, 8.4 Hz, 1H), 2.05 – 1.89 (m, 1H), 0.89 (d, *J* = 6.7 Hz, 3H).	
Chiral HPLC	Chiralpak AS-3, isocratic 9:1 nHex/IPA eluent<br>Retention time 35.5 min (minor), Retention time 39 min (major),	
MS (ESI+APCI)	m/z = 152.2 [M+H$^+$]	

General Procedure for Microfluidic Reaction (screening)

Photoredox stock solution: Benzylic alcohol radical source (250 µmol, 1.00 eq), (5*S*)-(-)-2,2,3-Trimethyl-5-benzyl-4-imidazolidinone -monohydrochloride 13.0 mg (50 µmol, 0.20 eq), Ir[4-tBu-ppy]$_3$ 1.00 mg (12.5 µmol, 0.005 eq) were placed inside MW-vial, sealed with a aluminium cap with rubber septum and a N_2-atmosphere was applied. *N*,*N*-Dimethylacetamide (degassed, stored under N_2) 1 mL Distilled water (degassed, stored under N_2) 134 µL, 2,6-Lutidine (degassed, stored under N_2) 15.0 µL (125 µmol, 0.50 eq), aldehyde (degassed, stored under N2) (500 µmol, 2.00 eq) were cannulated under strict N_2-atmosphere. The mixture was degassed by bubbling N_2 for 30 minutes.

Continuous flow reaction: A 2.5 mL SGE gastight syringe was flushed with N_2 three times. The previously prepared solution was taken into the syringe and quickly attached to the apparatus while applying a constant stream of N_2 through the apparatus. The solution was infused through the coil reactor to give the final residence times indicated in the graphs. Between the experiments one full residence time of reactor output was discarded to reach the steady state of the reactor.

$NaBH_4$- 160 mg (3 mmol, 10 eq) were dissolved in1 mL of 5% KOH, 200 µL sat. Aq. $NaHCO_3$ and 1.8 mL of HPLC-Water. 3 mL of this solution were taken into a 3 mL Norminject syringe and connected to the tubing leading into the Y-Connector piece and infused with the same flow rate as the photoredox solution.

Sampling: After reaching steady state, an aliquot was taken by collecting an idealized 10 µL of the photoredox-solution which were subsequently dissolved in 1 mL of HPLC-MeOH and the resulting mixture was analysed by HPLC-MS to check for the consumption of the starting benzylic alcohol radical source relative to the 2,6-lutidine signal which served as internal standard.

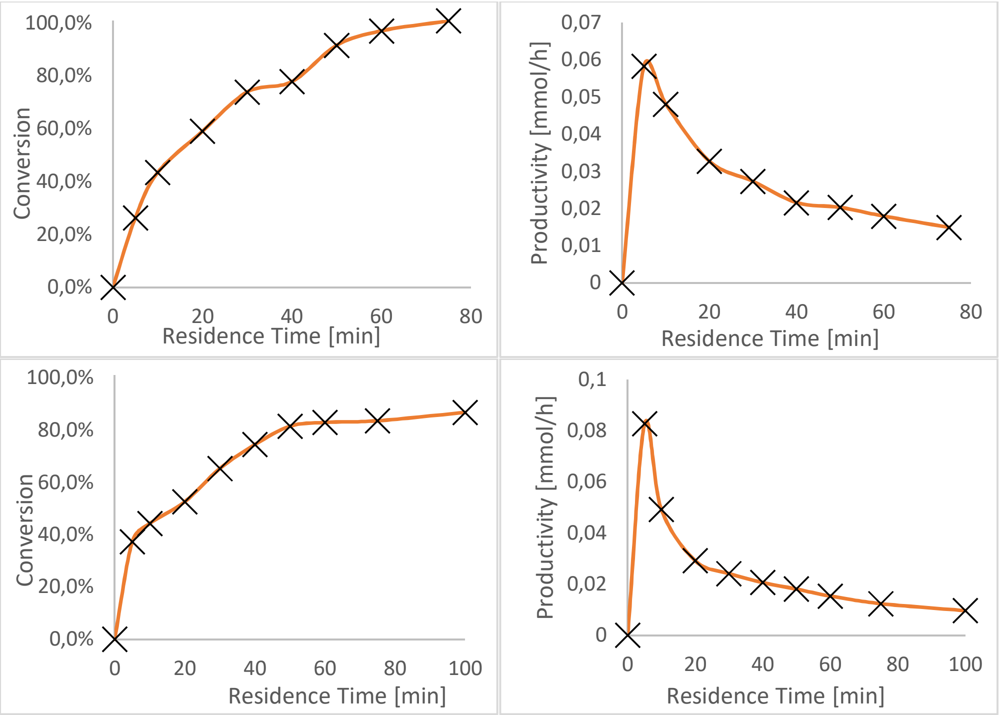

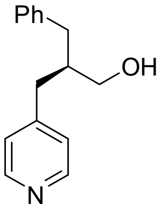

3aa

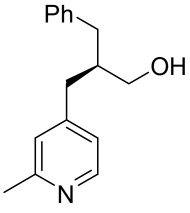

3ba

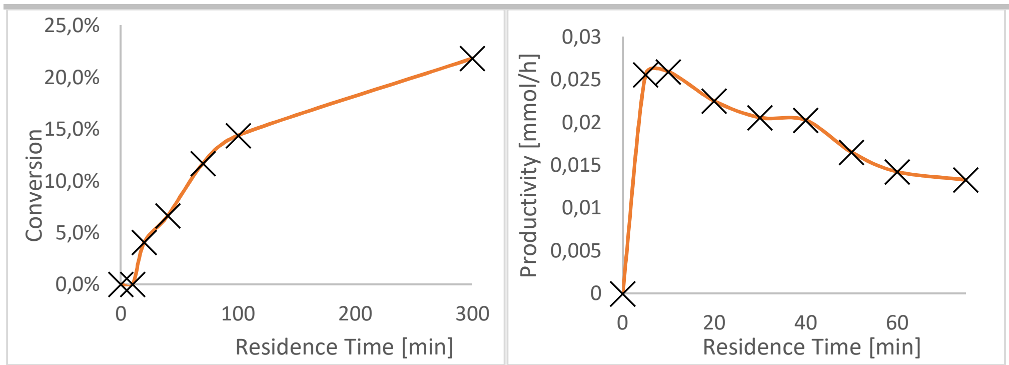
Conversion
25,0%
20,0%
15,0%
10,0%
5,0%
0,0%
0
100
200
300
Residence Time [min]
Productivity [mmol/h]
0,03
0,025
0,02
0,015
0,01
0,005
0
20
40
60
Residence Time [min]

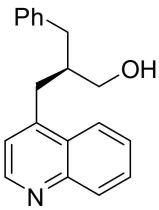
Ph
OH
N

3ca

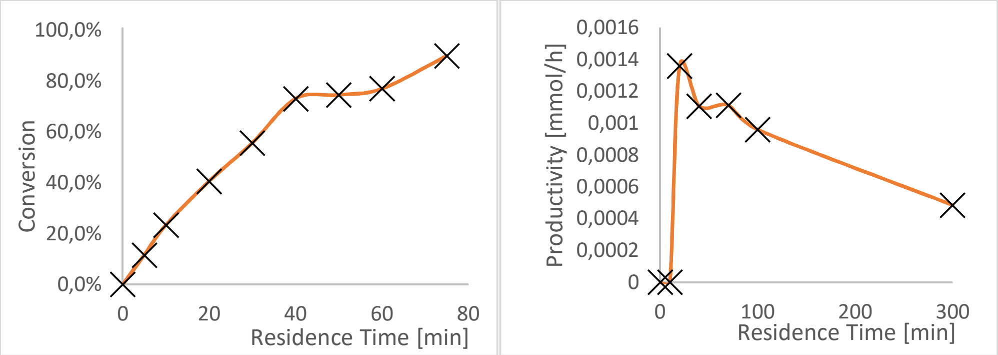
Conversion
100,0%
80,0%
60,0%
40,0%
20,0%
0,0%
0
20
40
60
80
Residence Time [min]
Productivity [mmol/h]
0,0016
0,0014
0,0012
0,001
0,0008
0,0006
0,0004
0,0002
0
100
200
300
Residence Time [min]

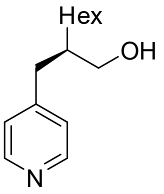
Hex
OH
N

3ab

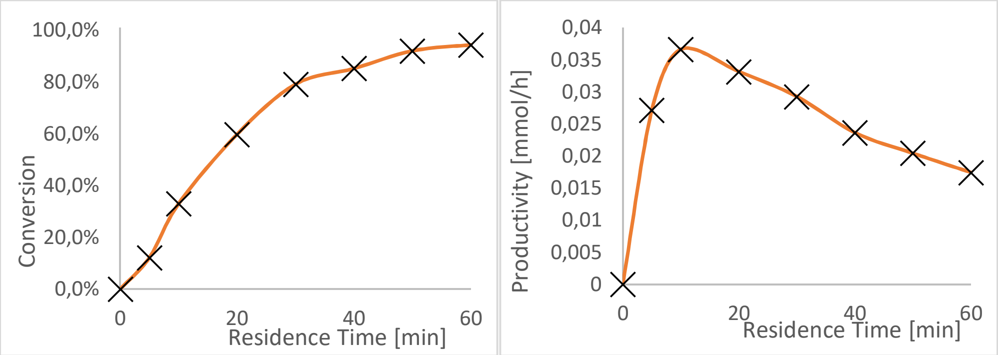
Conversion
100,0%
80,0%
60,0%
40,0%
20,0%
0,0%
0
20
40
60
Residence Time [min]
Productivity [mmol/h]
0,04
0,035
0,03
0,025
0,02
0,015
0,01
0,005
0
20
40
60
Residence Time [min]

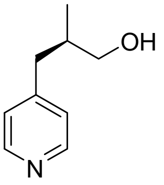
OH
N

3ac

General Procedure for Microfluidic Reactions (Synthesis)

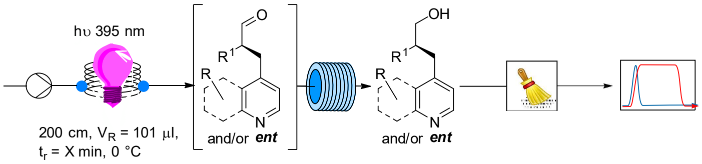

Photoredox stock solution: Benzylic alcohol radical source (250 µmol, 1.00 eq), (5*S*)-(-)-2,2,3-Trimethyl-5-benzyl-4-imidazolidinone -monohydrochloride 13.0 mg (50 µmol, 0.20 eq), Ir[4-tBu-ppy]$_3$ 1.00 mg (12.5 µmol, 0.005 eq) were placed inside MW-vial, sealed with a aluminium cap with rubber septum and a N_2-atmosphere was applied. N,N-Dimethylacetamide (degassed, stored under N2) 1 mL, Distilled water (degassed, stored under N_2) 134 µL, 2,6-Lutidine (degassed, stored under N_2) 15.0 µL (125 µmol, 0.50 eq), aldehyde (degassed, stored under N_2) (500 µmol, 2.00 eq) were cannulated under strict N_2-atmosphere. The mixture was degassed by bubbling N_2 for 30 minutes.

Continuous flow reaction: A 2.5 mL SGE gastight syringe was flushed with N_2 three times. The previously prepared solution (approx.. 0.2 M in concentration) was taken into the syringe and quickly attached to the apparatus while applying a constant stream of N_2 through the apparatus. The solution was infused through the coil reactor to give the final residence times indicated in the graphs. Between the experiments one full residence time of reactor output was discarded to reach the steady state of the reactor.

$NaBH_4$- 160 mg (3 mmol, 10 eq) were dissolved in1 mL of 5% KOH, 200 µL sat. Aq. $NaHCO_3$ and 1.8 mL of HPLC-Water. 3 mL of this solution were taken into a 3 mL Norminject syringe and connected to the tubing leading into the Y-Connector piece and infused with the same flow rate as the photoredox solution. The resulting output of the reactor was collected for the indicated amount of time in Table S3 and the remains were left overnight to be worked up. 5 mL of dichloromethane were added to the mixture and the aqueous phase was extracted two additional times with 5 mL of dichloromethane. The combined organic phases were dried over magnesium sulfate and the volatiles evaporated in vacuo (HV-rotavap). The resulting remains were purified using the automated protocols disclosed in the general procedure After vacuum centrifugation the compounds were isolated as stated in Table S3

Table S3: Results of the Microfluidic Experiments

Entry[a]	Molecule	Residence Time [min]	Time of collection [min]	ee [%]	Yield [mg], {%}
**1**	**3aa**	30	360	98	16.4 , 30%
**2**	**3ba**	35	420	99	23.1 , 38%
**3**[a]	**3ca**	1440	--	91	23.6 , 40%
**4**	**3ab**	40	480	94	20.8 , 37%
**5**	**3ac**[b]	25	300	96	22.3 , 58%

[a]Stop flow experiment, reaction time 24 h in a 1 mL Reactor, [b]due to the high water solubility of this compound, additional extraction steps have to be undertaken.

General Procedure for Mesofluidic (1 mL-Coil- Reactor)

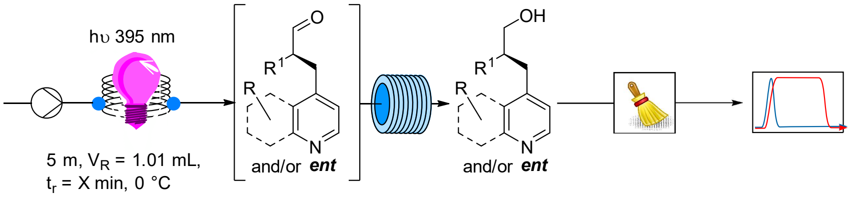

Photoredox stock solution: Benzylic alcohol radical source (2.50 mmol, 1.00 eq), (5*S*)-(-)-2,2,3-Trimethyl-5-benzyl-4-imidazolidinone -monohydrochloride 130 mg (500 µmol, 0.20 eq), Ir[4-tBu-ppy]$_3$ 10.00 mg (125 µmol, 0.005 eq) were Placed inside MW-vial, sealed with a aluminium cap with rubber septum and a N_2-atmosphere was applied. *N*,*N*-Dimethylacetamide (degassed, stored under N_2) 10 mL, Distilled water (degassed, stored under N_2) 1.35 mL, 2,6-Lutidine (degassed, stored under N_2) 150 µL (1.25 mmol, 0.50 eq), aldehyde (degassed, stored under N_2) (5.00 mmol, 2.00 eq) were cannulated under strict N_2-atmosphere. The mixture was degassed by bubbling N_2 for 30 minutes.

Continuous flow reaction: A 10 mL SGE gastight syringe was flushed with N_2 three times. The previously prepared solution (approx.. 0.2 M in concentration) was taken into the syringe and quickly attached to the apparatus while applying a constant stream of N_2 through the apparatus. The solution was infused through the coil reactor to give the final residence times indicated in the graphs. Between the experiments one full residence time of reactor output was discarded to reach the steady state of the reactor.

$NaBH_4$- 1.60 g (30 mmol, 10 eq) were dissolved in10 mL of 5% KOH, 2 mL sat. Aq. $NaHCO_3$ and 18 mL of HPLC-Water. 24 mL of this solution were taken into a 24 mL Norminject syringe and connected to the tubing leading into the Y-Connector piece and infused with the same flow rate as the photoredox solution. The resulting output of the reactor was collected for the indicated amount of time in Table S4 and the remains were left overnight to be worked up. 5 mL of dichloromethane were added to the mixture and the aqueous phase was extracted two additional times with 5 mL of dichloromethane. The combined organic phases were dried over magnesium sulfate and the volatiles evaporated in vacuo (HV-rotavap). The resulting remains were purified using the automated protocols disclosed in the general procedure. After vacuum centrifugation the compounds were isolated as stated in Table S4

Table S4: Results of the mesofluidic Experiments

Entry[a]	Molecule	Residence Time [min]	Time of collection [min]	ee [%]	Yield [mg], {%}
1	3aa	30	36	98	16.2 , 28%
2	3aa	60	72	98	26.8 , 47%
3[a]	3aa	60	72	96	35.5 , 62%
4	3ac	25	30	96	11.9 , 31%
5	3ac[b]	50	60	95	24.5 , 64%
6	3ac	75	90	95	29.5 , 77%
7[a]	3ac	50	60	93	11.9 , 31%

[a]Experiment conducted at room temperature, [b]due to the high water solubility of this compound, additional extraction steps have to be undertaken.

General Procedure for Segmented Mesofluidic Reactions (10 mL-Coil-Reactor)

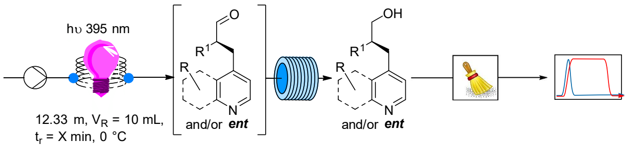

Photoredox stock solution: Benzylic alcohol radical source (2.50 mmol, 1.00 eq), (5*S*)-(-)-2,2,3-Trimethyl-5-benzyl-4-imidazolidinone -monohydrochloride 130 mg (500 µmol, 0.20 eq), Ir[4-tBu-ppy]$_3$ 10.00 mg (125 µmol, 0.005 eq) were Placed inside MW-vial, sealed with a aluminium cap with rubber septum and a N_2-atmosphere was applied. *N*,*N*-Dimethylacetamide (degassed, stored under N_2) 10 mL, Distilled water (degassed, stored under N_2) 1.35 mL, 2,6-Lutidine (degassed, stored under N_2) 150 µL (1.25 mmol, 0.50 eq), aldehyde (degassed, stored under N_2) (5.00 mmol, 2.00 eq) were cannulated under strict N_2-atmosphere. The mixture was degassed by bubbling N_2 for 30 minutes.

Continuous flow reaction: A 25 mL SGE gastight syringe was flushed with N_2 three times. The previously prepared solution (approx.. 0.2 M in concentration) was taken into the syringe and quickly attached to the apparatus while applying a constant stream of N_2 through the apparatus. The solution was infused through the coil reactor to give the final residence times indicated in the graphs. The Photoredox stock-solution was infused through the coil reactor in the time indicated in Table S5 and the syringe pump turned off. Afterwards heptane was infused with the same flow rate to give the final residence time indicated in Table S5.

$NaBH_4$- 1.60 g (30 mmol, 10 eq) were dissolved in10 mL of 5% KOH, 2 mL sat. Aq. $NaHCO_3$ and 18 mL of HPLC-Water. 24 mL of this solution were taken into a 24 mL Norminject syringe and connected to the tubing leading into the Y-Connector piece and infused with the same flow rate as the photoredox solution. The resulting output of the reactor was collected for the indicated amount of time in Table S4 and the remains were left overnight to be worked up. 5 mL of dichloromethane were added to the mixture and the aqueous phase was extracted two additional times with 5 mL of dichloromethane. The combined organic phases were dried over magnesium sulfate and the volatiles evaporated in vacuo (HV-rotavap). The resulting remains were purified using the automated protocols disclosed in the general procedure. After vacuum centrifugation the compounds were isolated as stated in Table S5

Table S5: Results of the segmented mesofluidic Experiments

Entry[a]	Molecule	Residence Time [min]	Time of collection [min]	ee [%]	Yield [mg], {%}
**1**	**3aa**	30	3:40	96	23.2 , 40%
**2**	**3aa**	60	7:20	97	20.1 , 35%
**3**	**3aa**	120	14:40	97	27.3 , 48%
**4**[a]	**3ac**	25	3	96	3.2 , 8%
**5**[a]	**3ac**[a]	50	6	97	9.5 , 25%
**6**[a]	**3ac**	75	90	95	14.5 , 38%

[a]due to the high water solubility of this compound, additional extraction steps have to be undertaken.

Procedure for Mesofluidic Reaction (10 mL-Coil-Reactor)

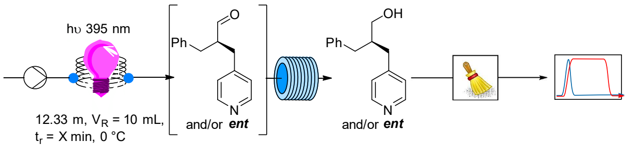

Photoredox stock solution: pyridine-4-methanol hydrobromide (7.5 mmol, 1.00 eq), (5*S*)-(-)-2,2,3-Trimethyl-5-benzyl-4-imidazolidinone -monohydrochloride 390 mg (1.25 mmol, 0.20 eq), Ir[4-tBu-ppy]$_3$ 30.00 mg (375 µmol, 0.005 eq) were placed inside MW-vial, sealed with a aluminium cap with rubber septum and a N_2-atmosphere was applied. *N*,*N*-Dimethylacetamide (degassed, stored under N_2) 30 mL, Distilled water (degassed, stored under N_2) 4.05 mL, 2,6-Lutidine (degassed, stored under N_2) 450 µL (1.25 mmol, 0.50 eq), 3-phenyl-propanal (degassed, stored under N_2) (15.0 mmol, 2.00 eq) were cannulated under strict N_2-atmosphere. The mixture was degassed by bubbling N_2 for 30 minutes.

Continuous flow reaction: A 25 mL SGE gastight syringe was flushed with N_2 three times. The previously prepared solution (approx.. 0.2 M in concentration) was taken into the syringe and quickly attached to the apparatus while applying a constant stream of N_2 through the apparatus. The solution was infused through the coil reactor to give the final residence times of 60 min. Two full residence times of reactor output were discarded.

$NaBH_4$- 1.60 g (30 mmol, 10 eq) were dissolved in10 mL of 5% KOH, 2 mL sat. Aq. $NaHCO_3$ and 18 mL of HPLC-Water. 24 mL of this solution were taken into a 24 mL Norminject syringe and connected to the tubing leading into the Y-Connector piece and infused with the same flow rate as the photoredox solution. An aliquot from the reactor output revealed 60% consumption of the starting material. The resulting output of the reactor was collected for 72 min (1.2 reactor volumes, 2.5 mmol of hypothetical yield) and the remains were left overnight. 50 mL of dichloromethane were added to the mixture and the aqueous phase was extracted two additional times with 50 mL of dichloromethane. The combined organic phases were dried over magnesium sulfate and the volatiles evaporated in vacuo (HV-rotavap). The resulting remains were purified using preparative RP18-Silica and a Gradient of water/acetonitrile with 0.5 mM NH_4HCO_3. After vacuum centrifugation 220 mg of Product was isolated in 38% Yield and with an *ee* of 92%.

```
=====================================================================
Acq. Operator   : SYSTEM                        Seq. Line :   1
Acq. Instrument : HPLC                           Location : Vial 72
Injection Date  : 5/18/2021 1:28:38 PM                Inj :   1
                                               Inj Volume : 10.000 µl
Different Inj Volume from Sample Entry!  Actual Inj Volume : 4.000 µl
Method          : C:\CHEM32\1\DATA\P15036\P15036 2021-05-18 13-26-04\P15036_CHIRALPACK_IA_90-
                  10.M (Sequence Method)
Last changed    : 5/18/2021 1:26:04 PM by SYSTEM
Additional Info : Peak(s) manually integrated
```

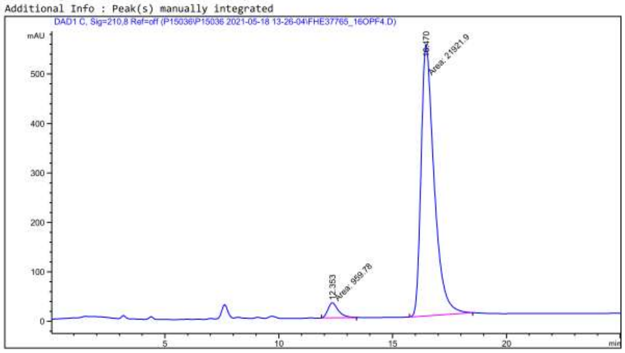


```
=====================================================================
                         Area Percent Report
=====================================================================

Sorted By             :      Signal
Multiplier            :      1.0000
Dilution              :      1.0000
Use Multiplier & Dilution Factor with ISTDs


Signal 1: DAD1 C, Sig=210,8 Ref=off

Peak RetTime Type  Width     Area      Height     Area
  #   [min]        [min]   [mAU*s]     [mAU]        %
----|-------|----|-------|----------|----------|--------|
   1  12.353 MM    0.5244  959.78027   30.50532   4.1945
   2  16.470 MM    0.6662 2.19219e4   548.44763  95.8055

Totals :                  2.28817e4   578.95295

=====================================================================
                          *** End of Report ***
```

Calculation of Productivity and Space-Time-Yield

Entry	Method	Yield	$t_{collection}$ until 250 µmol [h]	P [mmol/h]	Rel. Factor	Residence time t_R [h]	STY [mmol/ml*h]	Rel-Factor
**1**	Batch (1.2 mL)	43%	24	4.5E-3	1	24	0,003673	1
**2**	Microfluidic	43%	6	1.3E-2	2.8	0.5	0,123	33,48837
**3**	1 mL Meso	47%	1.2	9.8E-2	22	1	0,09635	26,23256
**4**	10 mL Meso	38%	0.12	7.9E-1	176	1	0,0779	21,2093

$$\mathrm{P} = \frac{\mathrm{n_{prod}} * \%\mathrm{Y}}{t_{collection} * 100\%}$$

$$\mathrm{STY} = \frac{\mathrm{n_R} * \%\mathrm{Y}}{V_R * t_R * 100\%}$$

As to rule out effects of reaction scale that will change isolated yield (e.g. purification methods, work-up methods). All of the reactions were performed on the same scale by collecting reactor outputs for the given time it would take to have the same amount of moles as under batch conditions (250 µmol). Then the above formula was used to calculate P, in which n_{prod} stands for the (idealized) quantitative yield of product. %Y stands for isolated yield in % and $t_{collection}$ stands for collection time until the idealized n_{prod} would be collected.

When calculating the space-time-yield (STY) the above formula was used, where n_R and V_R are the moles and volume inside the reactor, t_R is residence time, %Y stands for isolated yield in %.

Telescoping: Inline oxidation to the carboxylic acid then extraction

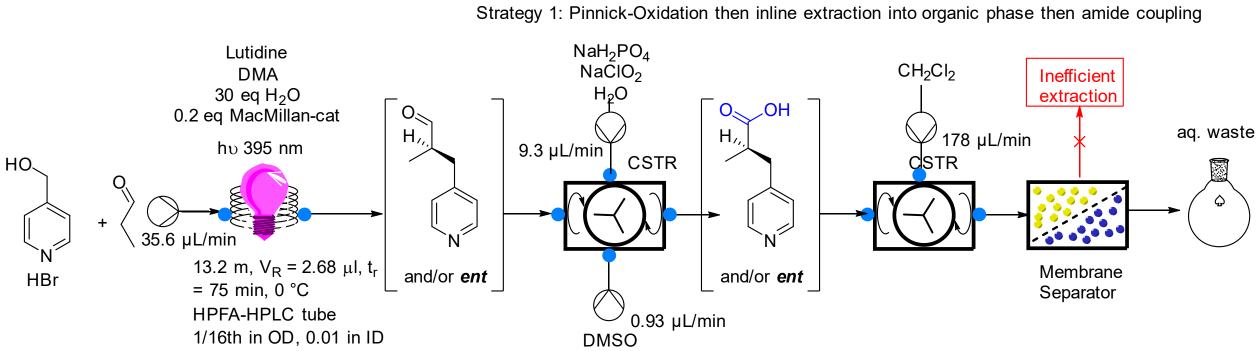

Photoredox stock solution: Benzylic alcohol radical source 935 mg (4.92 mmol, 1.00 eq), (5*S*)-(-)-2,2,3-Trimethyl-5-benzyl-4-imidazolidinone 215 mg (998 µmol, 0.20 eq), Ir[4-*t*Bu-ppy]$_3$ 20.25 mg (24.6 µmol, 0.005 eq) were placed inside 25 mL conical flask, sealed with rubber septum and a N_2-atmosphere was applied. *N,N*-Dimethylacetamide (degassed, stored under N_2) 20 mL, Distilled water (degassed, stored under N_2) 2.66 µL, 2,6-Lutidine (degassed, stored under N_2) 285 µL (2.46 mmol, 0.50 eq were added using syringe in the flask under strict N_2-atmosphere. The mixture was degassed by bubbling N_2 for 30 minutes. Propionaldehyde (degassed, stored under N_2) 1.74 mL (24.6 mmol, 2.00 eq) has been also degassed for 30 minutes and added to the conical flask: the resulting mixture has been degassed 10 minutes.

Continuous flow photoredox reaction: A 10 mL SGE gastight syringe was flushed with N_2 three times. The previously prepared solution (0.183 M) was taken into the syringe and quickly attached to the apparatus while applying a constant stream of N_2 through the apparatus. The first 75 min of elution has been wasted.

Continuous flow Pinnick-oxidation: In a 10 mL SGE syringe was taken up a 10 mL of solution of 1.83 M sodium dihydrophosphate mixed with 1.83 M sodium chlorite in distilled water. The mixture was infused into the first CSTR after infusing DMSO into the same CSTR that was previously taken up in a 250 µL SGE syringe. The first residence time of infusion (40 min) is discarded. The output of the CSTR is connected to the next CSTR in the cascade.

Continuous Extraction: dichloromethane (5 volume equivalents) was infused in second CSTR. Residence time (extraction time) is approximately 9 min.

Continuous Phase separation: The output of the second CSTR was infused into a Zaiput continuous Phase separator (Laboratory scale) with a 0.5 µm hydrophobic membrane inside. After experiencing successful phase separation two full residence times were infused and the mixture was collected. After checking the organic phase for the carboxylic acid (tlc 9:1 dichloromethane/methanol) also the aqueous phase was checked, and the product was found inside it. These findings discouraged the further development of an organic phase driven amide coupling due to the excellent water solubility of the carboxylic acid.

Telescoping: First extraction then oxidation then aqueous amide coupling

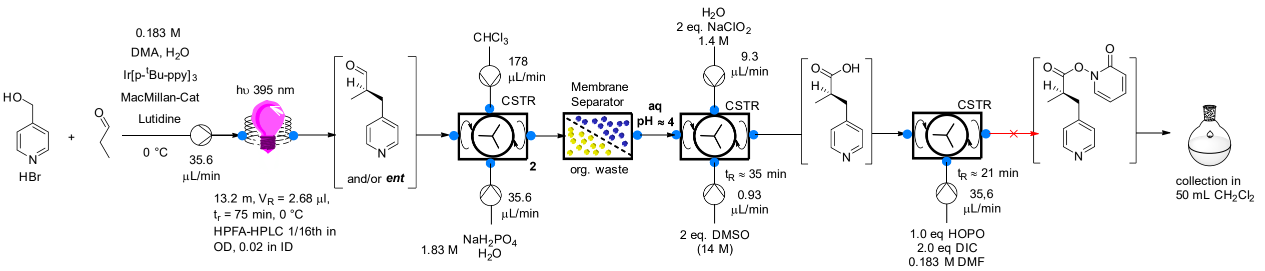

Photoredox stock solution: Benzylic alcohol radical source 935 mg (4.92 mmol, 1.00 eq), (5*S*)-(-)-2,2,3-Trimethyl-5-benzyl-4-imidazolidinone 215 mg (998 µmol, 0.20 eq), Ir[4-*t*Bu-ppy]3 20.25 mg (24.6 µmol, 0.005 eq) were placed inside 25 mL conical flask, sealed with rubber septum and a N_2-atmosphere was applied. *N,N*-Dimethylacetamide (degassed, stored under N_2) 20 mL, Distilled water (degassed, stored under N_2) 2.66 µL, 2,6-Lutidine (degassed, stored under N_2) 285 µL (2.46 mmol, 0.50 eq were added using syringe in the flask under strict N_2-atmosphere. The mixture was degassed by bubbling N_2 for 30 minutes. Propionaldehyde (degassed, stored under N_2) 1.74 mL (24.6 mmol, 2.00 eq) has been also degassed for 30 minutes and added to the conical flask: the resulting mixture has been degassed 10 minutes.

Continuous flow photoredox reaction: A 10 mL SGE gastight syringe was flushed with N_2 three times. The previously prepared solution (0.183 M) was taken into the syringe and quickly attached to the apparatus while applying a constant stream of N_2 through the apparatus. The first 75 min of elution has been wasted.

Continuous Extraction: In a 10 mL SGE syringe was taken up 10 mL of a solution of 1.83 M sodium dihydrophosphate. dichloromethane (5 volume equivalents) was infused into the first two CSTRs. Residence time (extraction time) is approximately 18 min.

Continuous Phase separation: The output of the second CSTR was infused into a Zaiput continuous Phase separator (Laboratory scale) with a 0.5 µm hydrophobic membrane inside. This time the aqueous output was of interest and thus was further infused into a third CSTR.

Continuous flow Pinnick-oxidation: In a 10 mL SGE syringe was taken up a 10 mL of solution of 1.83 M sodium chlorite in distilled water. The mixture was infused into the third CSTR after infusing DMSO into the same CSTR that was previously taken up in a 250 µL SGE syringe. The first residence time of infusion 35 min) is discarded. The output of the third CSTR is infused into the fourth CSTR for the aqueous amide coupling.

Continuous flow aqueous amide coupling: into a 10 mL SGE syringe was taken up a solution of 546 mg (4.92 mmol, 1.00 eq) 2-hydroxy pyridine oxide (HOPO) together with 1.54 mL (9.84 mmol, 2.00 eq) diisopropylcarbodiimide in 27 mL dimethylformamide (c=0.183 M). This solution was then infused into the fourth CSTR and the first residence time was discarded. The output was collected in dichlormethane under stirring for 2 full residence times. After unsuccessful HOPO-active ester formation (indicated by tlc, acid remained in aqueous phase) the aqueous phase was evaporated and the remains were purified by normal phase flash column chromatography (5% methanol in dichloromethane) to give the carboxylic acid product in 74% yield.

Fully Telescoped fully continuous Synthesis of the API

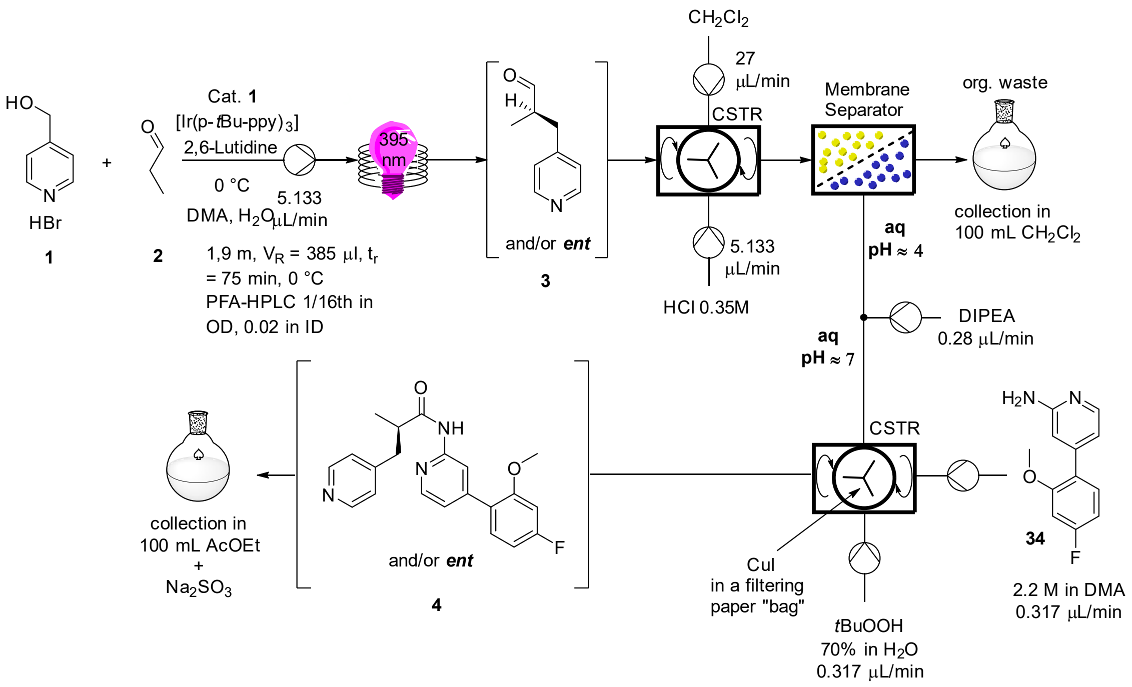

Photoredox stock solution: Benzylic alcohol radical source 290 mg (1.53 mmol, 1.00 eq), (5*S*)-(-)-2,2,3-Trimethyl-5-benzyl-4-imidazolidinone 66.6 mg (305 µmol, 0.20 eq), Ir[4-*t*Bu-ppy]$_3$ 6.3 mg (7.6 µmol, 0.005 eq) were placed inside 25 mL conical flask, sealed with rubber septum and a N_2-atmosphere was applied. *N,N*-Dimethylacetamide (degassed, stored under N_2) 6.1 mL, Distilled water (degassed, stored under N_2) 825 µL, 2,6-Lutidine (degassed, stored under N_2) 89.0 µL (763 µmol, 0.50 eq were added using syringe in the flask under strict N_2-atmosphere. The mixture was degassed by bubbling N_2 for 30 minutes. Propionaldehyde (degassed, stored under N_2) 547µL (7.63 mmol, 2.00 eq) has been also degassed for 30 minutes and added to the conical flask: the resulting mixture has been degassed 10 minutes.

Continuous flow photoredox reaction: A 10 mL SGE gastight syringe was flushed with N_2 three times. The previously prepared solution (0.183 M) was taken into the syringe and quickly attached to the apparatus while applying a constant stream of N_2 through the apparatus. The first 75 min elution has been wasted.

Continuous flow extraction: a 25 mL SGE gastight syringe has been flushed with N_2 three times and then filled with dichloromethane, another 10 mL SGE gastight syringe has been flushed with nitrogen three times and then equipped with 0.35M HCl (aq.). Under strong flux of nitrogen in a CSTR equipped with a stirring bar, were rapidly connected the syringe with DCM, the syringe with 0.35M HCl and the output of the photoredox flow reaction; the pH at the output of the CSTR has been tested between 3 and 4 with pH-indicator paper. The membrane separator has been connected, fluxed for some minutes with nitrogen from the output and then connected to the CSTR: the organic output of the CSTR was collected in a 50 mL flask and discarded. A complete reactor volume has been wasted (80 minutes).

Continuous flow neutralization: the pH at the aqueous output of the extraction has been tested 4 with pH indicator paper. The output has been connected to a T connecter. A 250 µL syringe has been fluxed with nitrogen three times, charged with *N*,*N*-Diisopropylethylamine and connected to the T connecter. pH at the output of the T-shaped connector has been tested to be 7. The output of this T-connector was connected to the last CSTR, containing a filtering paper bag loaded with CuI (1.00 g, 5.25 mmol).

Continuous flow amidation: In a vial cold 4-(4-fluoro-2-methoxyphenyl)pyridin-2-amine 100 mg (458 µmol, 1.00 eq) was diluted with 210 µL of DMA to obtain an homogeneous 2.2 M solution; this solution has been charged in a 250 µL SGE syringe and connected to the CSTR.

In another 250 µL SGE syringe was added the same volume of *Tert*-butyl hydrogen peroxide 70% and connected to the CSTR.

The CSTR output was dropped inside a flask containing 20 mL of AcOEt and 500 mg of Na_2SO_3. After 180 min the flask has been changed with another one containing 20 mL of AcOEt and 500 mg of Na_2SO_3.

The product has been purified by preparative-TLC using 8% EtOH in DCM (violet spot under UV light).

Yield 22%

R*f* = 0.52 8% EtOH in DCM
Purified by Preparative TLC
^{1}H NMR (300 MHz, CDCl3) δ 8.51 (s, 2H), 8.32 (s, 1H), 8.22 (d, J = 5.2 Hz, 1H), 7.82 (d, J = 14.6 Hz, 2H), 7.34 (t, J = 7.4 Hz, 2H), 7.24 – 7.17 (m, 1H), 6.87 – 6.63 (m, 2H), 3.85 (d, J = 7.5 Hz, 5H), 3.20 – 3.03 (m, 1H), 2.91 – 2.54 (m, 2H), 1.29 (d, J = 4.7 Hz, 3H).
^{13}C NMR (75 MHz, CDCl3) δ 173.63, 164.16 (d, J = 248.2 Hz), 151.29, 150.08, 148.42 (d, J = 8.2 Hz), 147.34, 131.74 (d, J = 10.1 Hz), 124.46, 121.11, 114.45, 107.69 (d, J = 21.4 Hz), 99.82 (d, J = 25.8 Hz), 56.03, 43.85, 39.34, 29.85, 18.06.
^{19}F NMR (282 MHz, CDCl3) δ -109.66.
Exact Mass = 366.1611 m/z (TOF ESI+; calculated: 366.1610 m/z)
Chiral HPLC: PHENOMENEX LUX 3u Amylose-1, 70 Hex 30 EtOH 0,1 DEA, 1 mL/min, P=97 bar. Retention time: 9.287 min, 13.313 min *ee 95%*

Appendix ^{1}H-NMR-Spectra

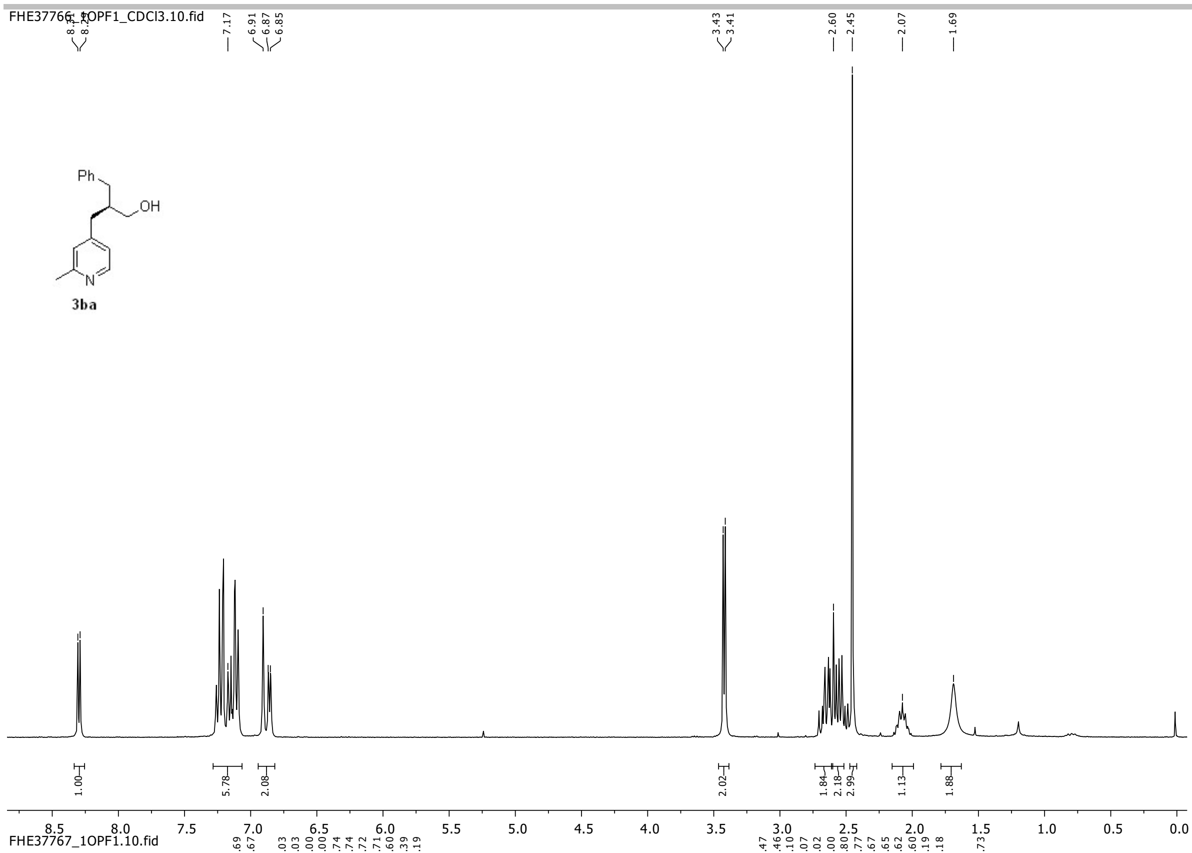
FHE37766_1OPF1_CDCl3.10.fid
3ba
8.5 8.0 7.5 7.0 6.5 6.0 5.5 5.0 4.5 4.0 3.5 3.0 2.5 2.0 1.5 1.0 0.5 0.0

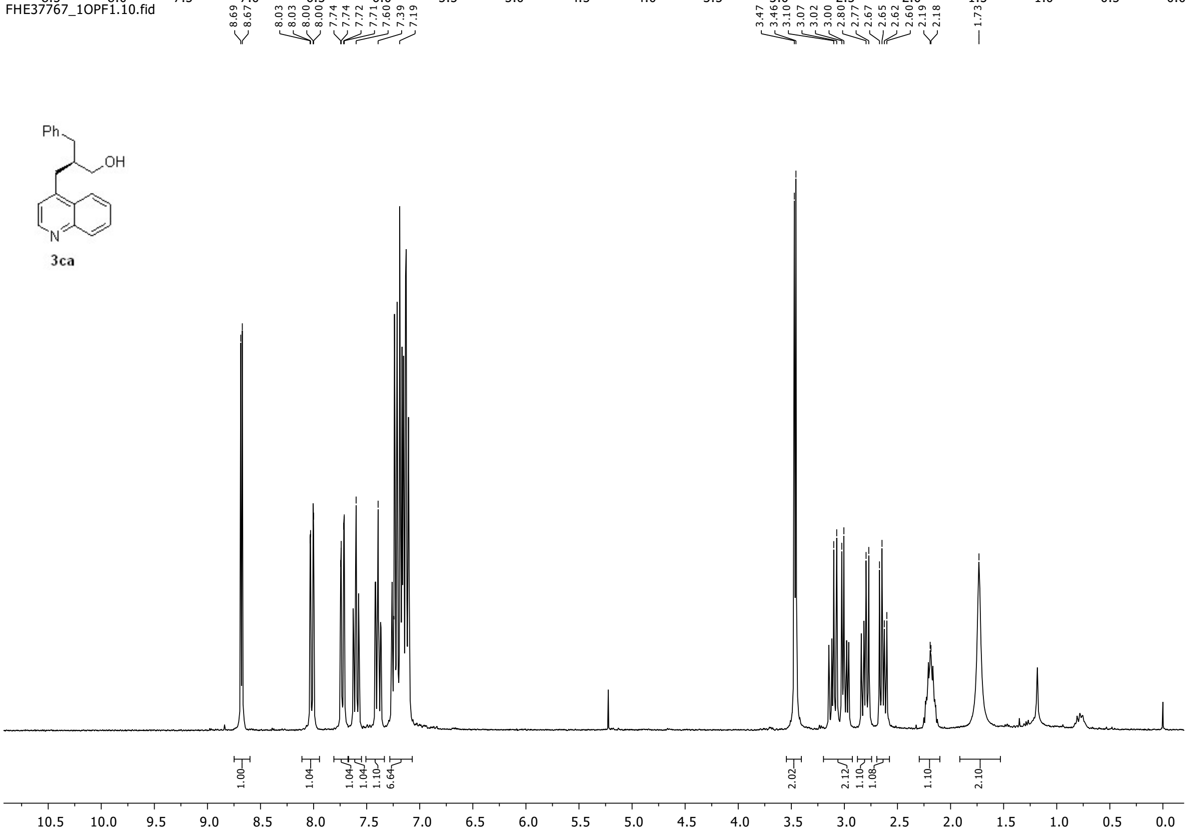
FHE37767_1OPF1.10.fid
3ca
10.5 10.0 9.5 9.0 8.5 8.0 7.5 7.0 6.5 6.0 5.5 5.0 4.5 4.0 3.5 3.0 2.5 2.0 1.5 1.0 0.5 0.0

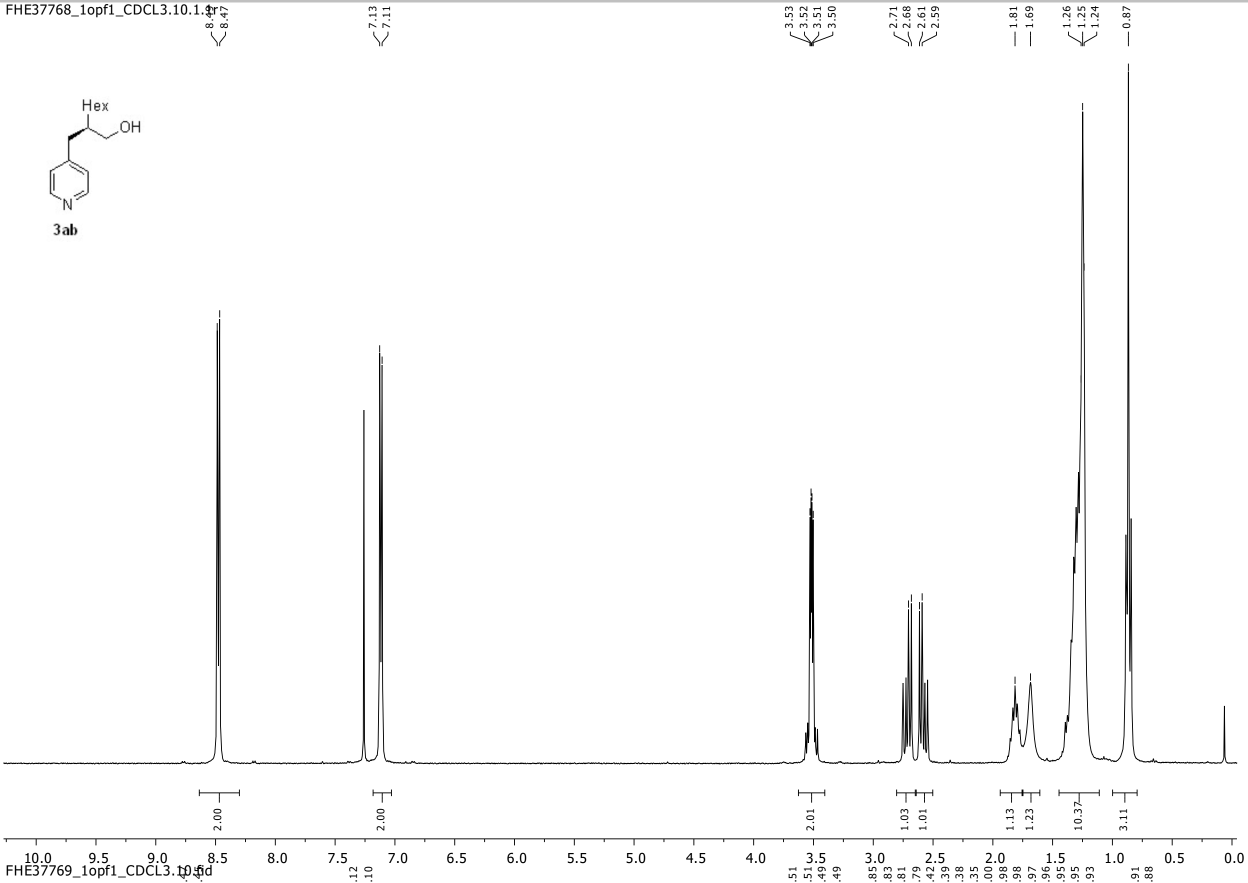
FHE37768_1opf1_CDCL3.10.1.1r
8.47
7.13
7.11
3.53
3.52
3.51
3.50
2.71
2.68
2.61
2.59
1.81
1.69
1.26
1.25
1.24
0.87
Hex
OH
N
3ab
2.00
2.00
2.01
1.03
1.01
1.13
1.23
10.37
3.11
10.0 9.5 9.0 8.5 8.0 7.5 7.0 6.5 6.0 5.5 5.0 4.5 4.0 3.5 3.0 2.5 2.0 1.5 1.0 0.5 0.0

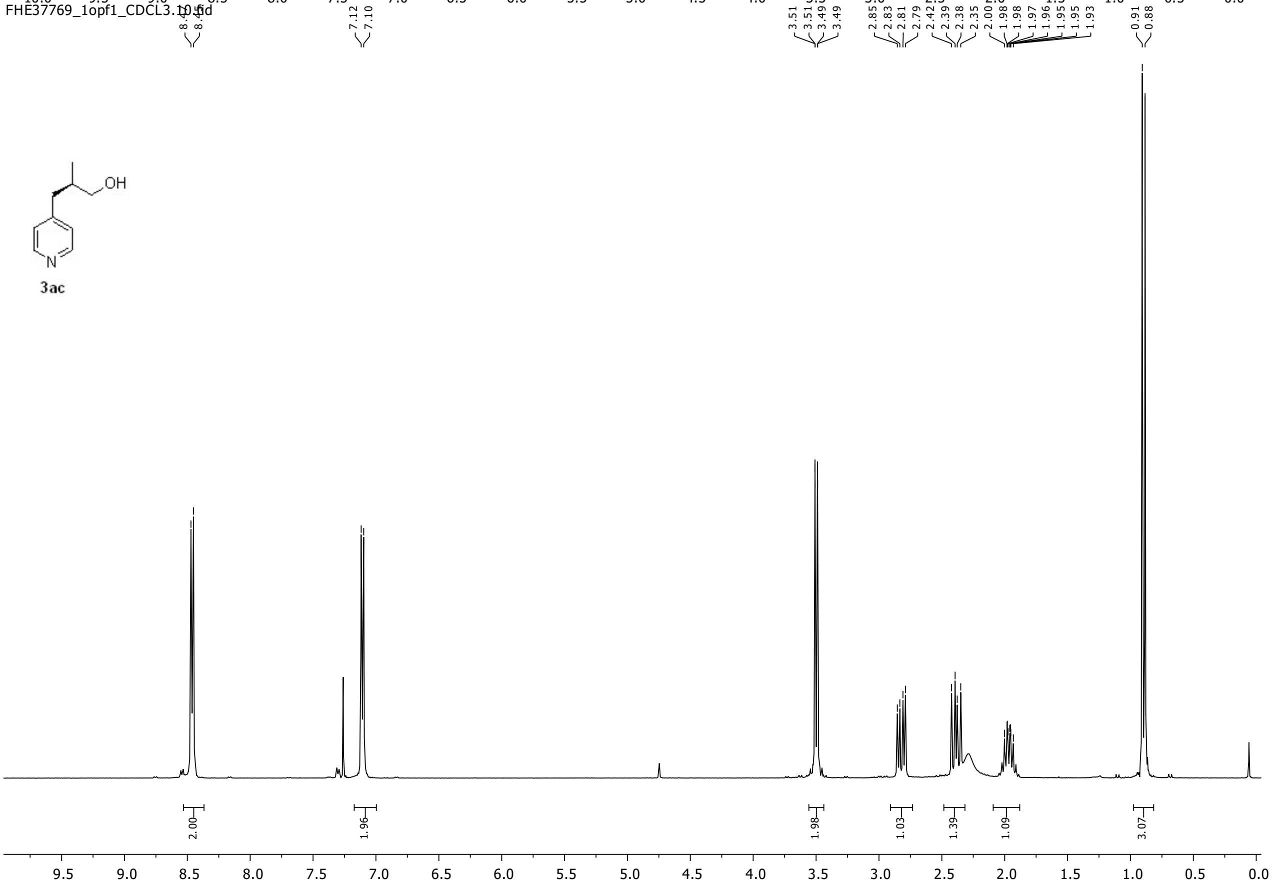
FHE37769_1opf1_CDCL3.10.fid
7.12
7.10
3.51
3.51
3.49
3.49
2.85
2.83
2.81
2.79
2.42
2.39
2.38
2.35
2.00
1.98
1.98
1.97
1.96
1.95
1.95
1.93
0.91
0.88
OH
N
3ac
2.00
1.96
1.98
1.03
1.39
1.09
3.07
9.5 9.0 8.5 8.0 7.5 7.0 6.5 6.0 5.5 5.0 4.5 4.0 3.5 3.0 2.5 2.0 1.5 1.0 0.5 0.0

Telescoped Carboxylic acid API precursor after unsuccessful amide coupling.

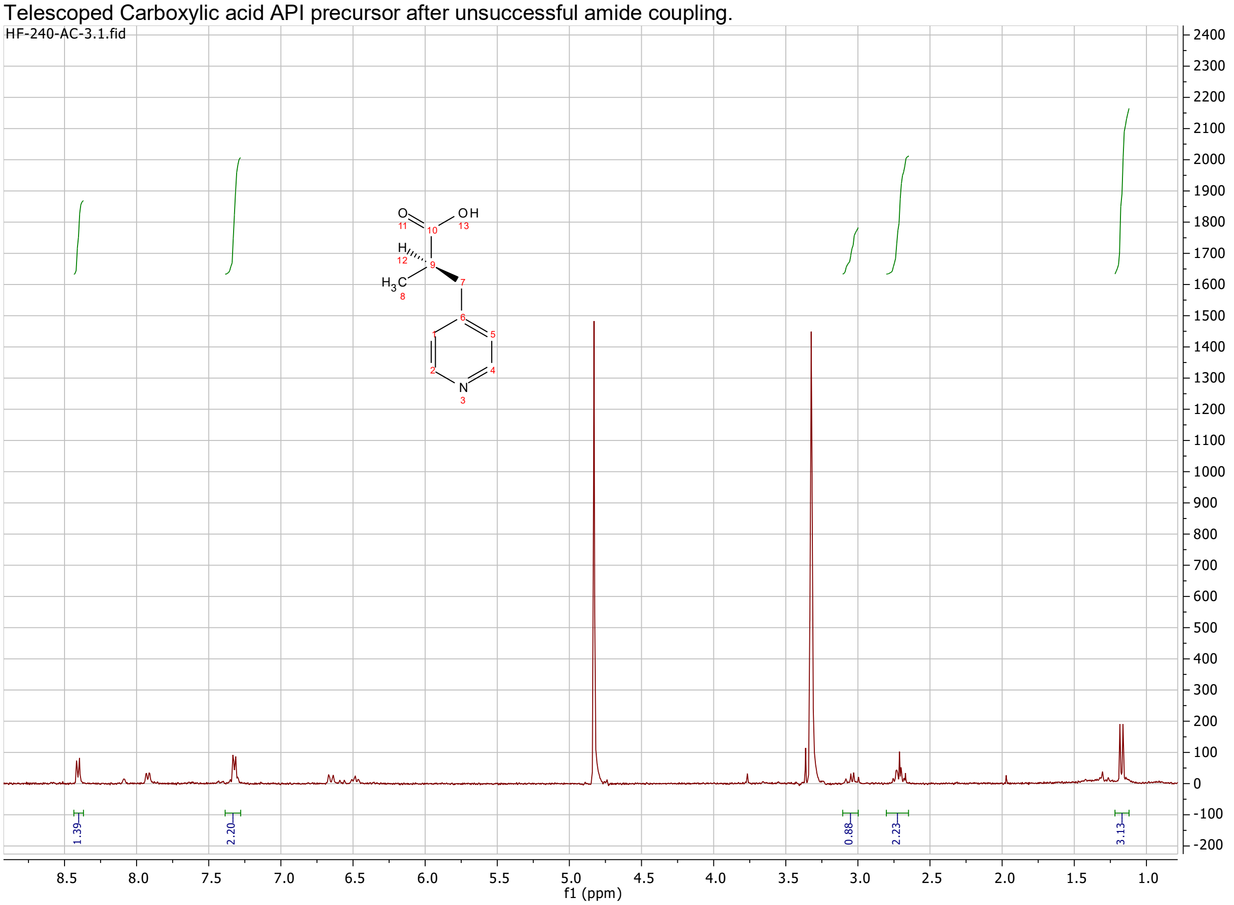

^{1}H-NMR of API

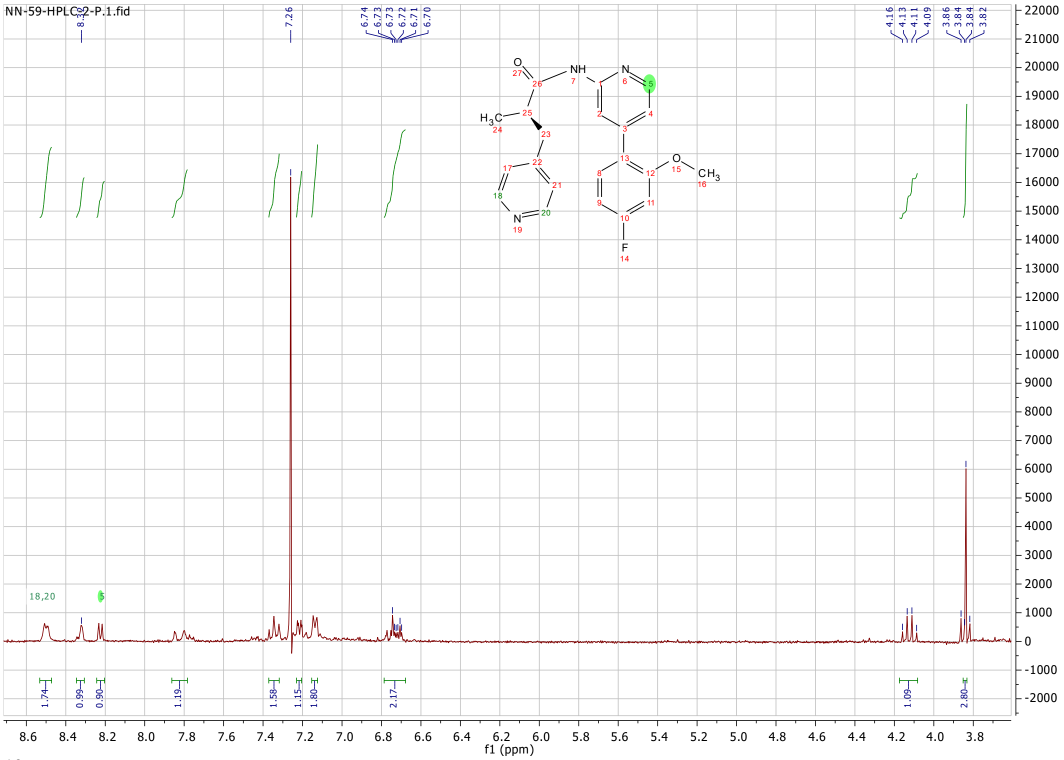

^{13}C-NMR of API

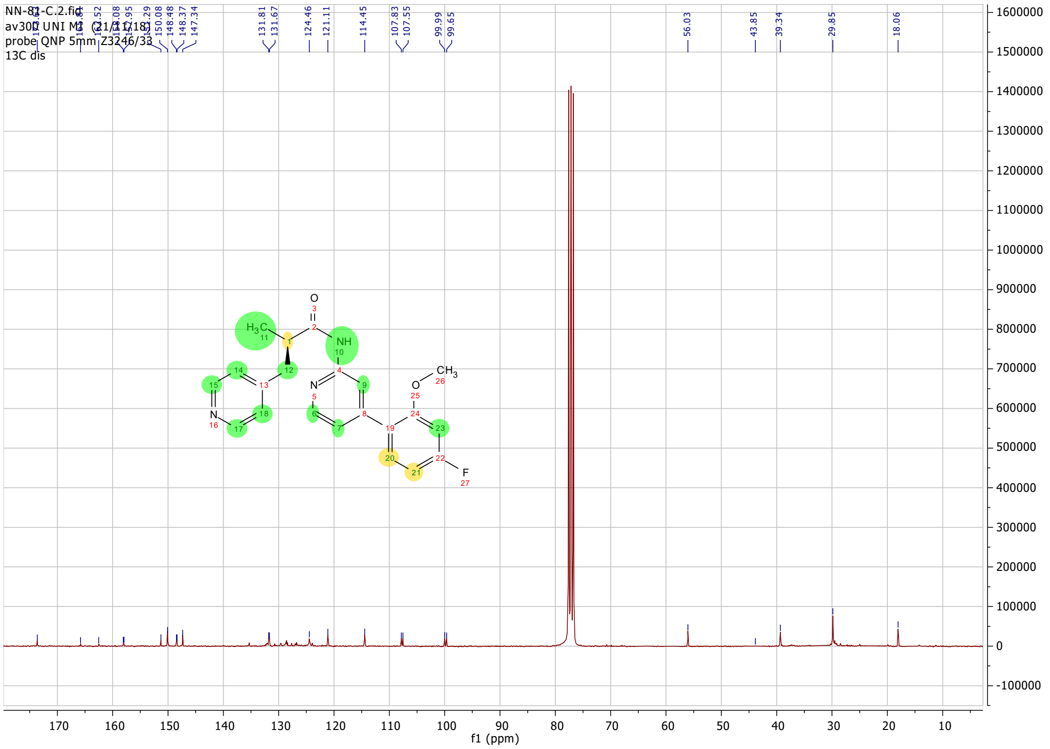

F-NMR of API

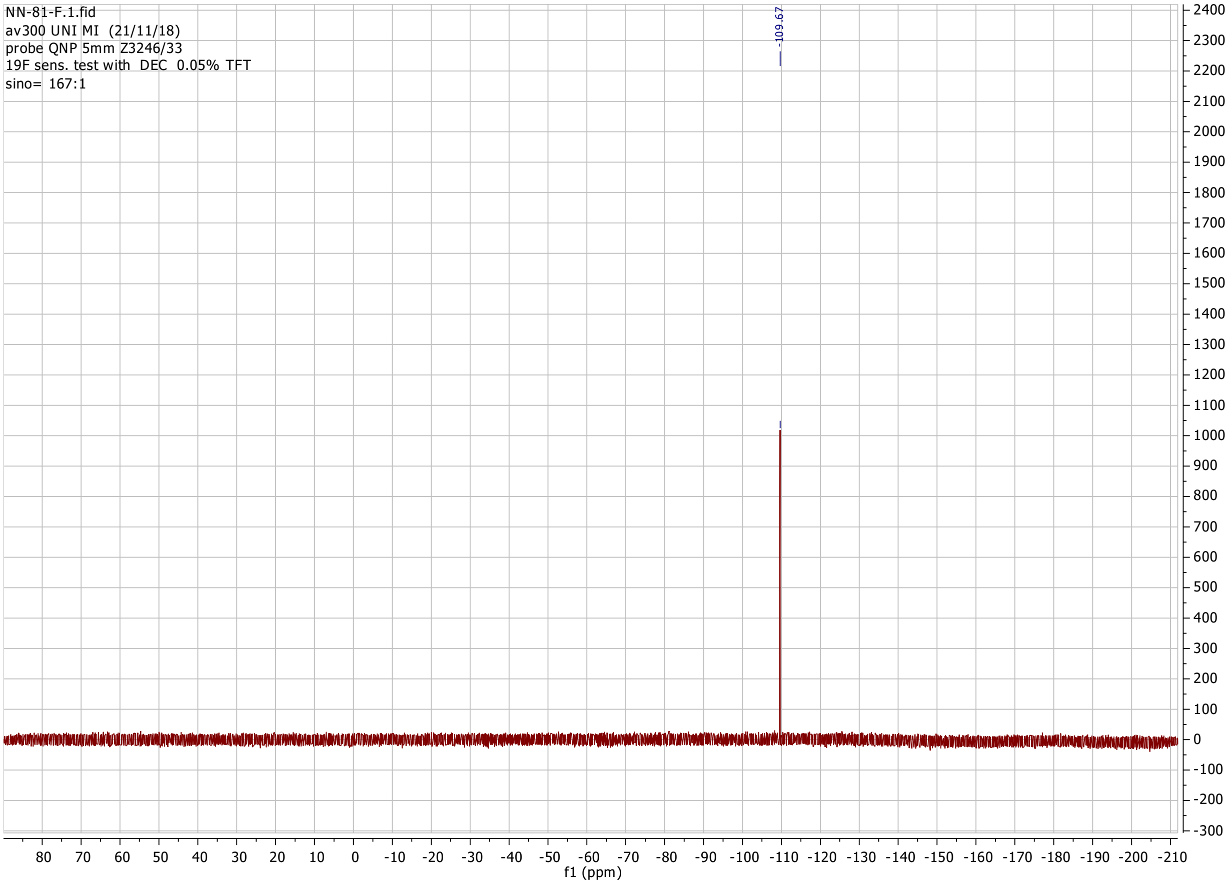

HR-MS of API

Elemental Composition Report **Page 1**

Single Mass Analysis
Tolerance = 5.0 PPM / DBE: min = -2.5, max = 200.0
Element prediction: Off
Number of isotope peaks used for i-FIT = 5

Monoisotopic Mass, Even Electron Ions
2 formula(e) evaluated with 1 results within limits (all results (up to 1000) for each mass)
Elements Used:
C: 21-21 H: 20-21 N: 3-3 O: 2-2 F: 1-1 Na: 0-2
NN-81 57 (1.139) AM2 (Ar,40000.0,0.00,0.00); Cm (10:103) 1: TOF MS ES+ 5.84e+007

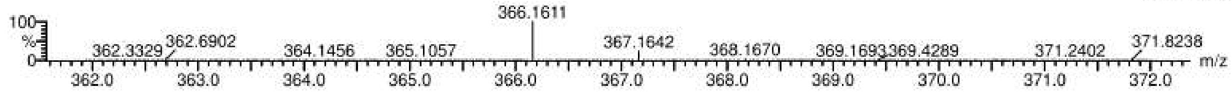

Mass	Calc. Mass	mDa	PPM	DBE	i-FIT	Norm	Conf(%)	Formula
Minimum:				-2.5				
Maximum:		5.0	5.0	200.0				
366.1611	366.1618	-0.7	-1.9	12.5	2787.4	n/a	n/a	C21 H21 N3 O2 F

uHPLC-Traces coupled with APCI/ESI-MS-spectra

Compound **3aa**

Sample name: FHE37765_4OpF1
Analysis method: TAROSFASTSTANDARD_basic.M
Injection volume: 2.000
Injection date: 3/10/2021 1:04:19 PM
Acq. operator: chemist
Instrument: uHPLC Agilent 1290 Infinity

Description: Column: ACQUITY UHPLC BEH C18 (1.7µm) 2.1mmx50mm T30°C Detection: DAD + 6120 Quadrupole Flow: 1.0mL/min. SolventA: Water+0.005 NH4HCO3 SolventB:MeCN;Gradient:0min.2%B;0.2min.2%B;2.0min.98%B;2.2min.98%B., 2.21min.2%B;2.5min.2%B;200-300nm

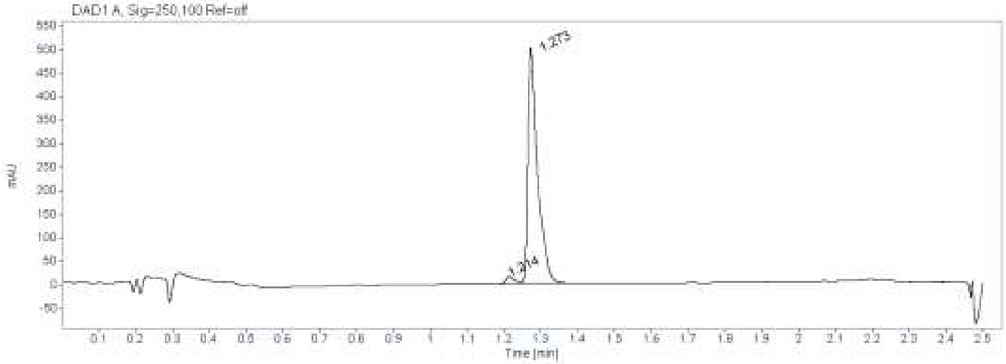

MSD1 SPC, MM-ES+APCI

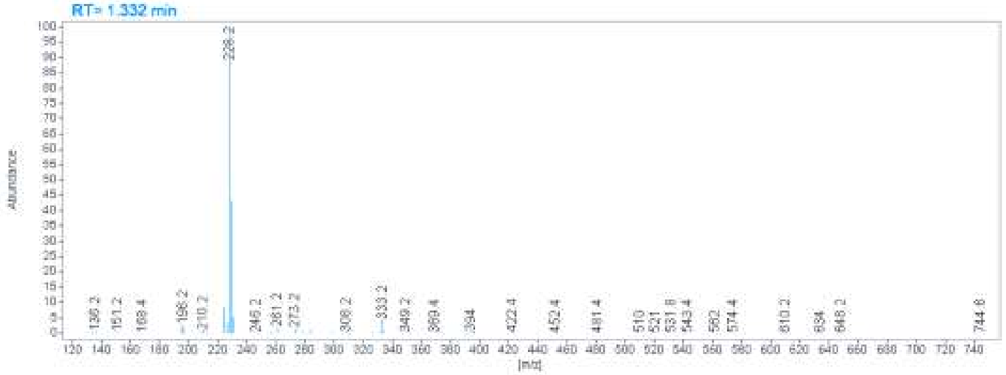

Signal: DAD1 A, Sig=250,100 Ref=off

RT [min]	Peak Area Percent
1.214	2.58
1.273	97.42

Compound **3ba**

**Sample name:**	FHE37766_4OpF1	**Injection date:**	3/10/2021 1:11:18 PM
**Analysis method:**	TAROSFASTSTANDARD_basic .M	**Acq. operator:**	chemist
**Injection volume:**	2.000	**Instrument:**	uHPLC Agilent 1290 Infinity

Description: Column: ACQUITY UHPLC BEH C18 (1.7µm) 2.1mmx50mm T30°C Detection: DAD + 6120 Quadrupole Flow: 1.0mL/min. SolventA: Water+0.005 NH4HCO3 SolventB:MeCN;Gradient:0min.2%B;0.2min.2%B;2.0min.98%B;2.2min.98%B,.2.21min.2%B;2.5min.2%B;200-300nm

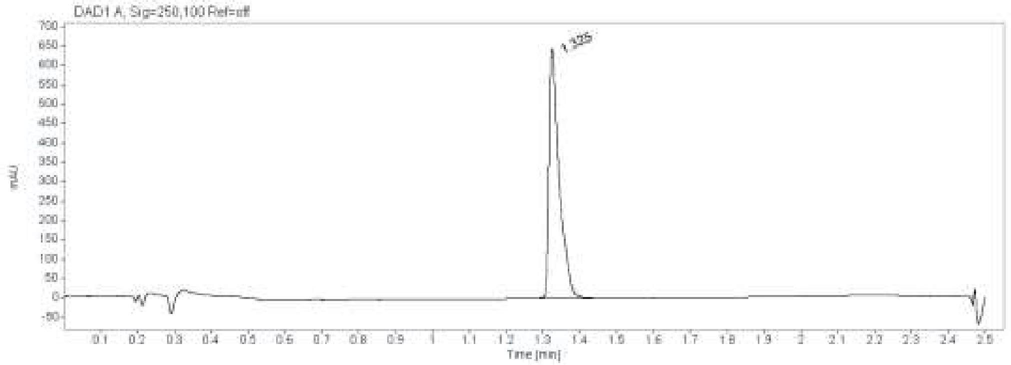

MSD1 SPC, MM-ES+APCI

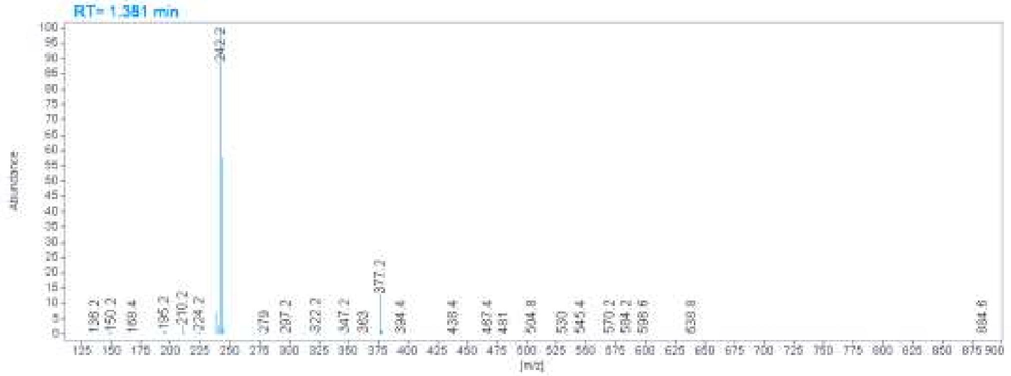

Signal: DAD1 A, Sig=250,100 Ref=off

RT [min]	Peak Area Percent
1.325	100.00

TAROS

Sample name: FHE37767_2OPF1
Injection date: 2/3/2021 6:07:51 PM
Analysis method: TAROSFASTSTANDARD_lowmass.M
Acq. operator: chemist
Injection volume: 5.000
Instrument: uHPLC Agilent 1290 Infinity
Description: Column: ACQUITY UHPLC BEH C18 (1.7μm) 2.1mmx50mm T40°C Detection: DAD + 6120 Quadrupole Flow: 1.0mL/min. SolventA: Water+0.1% formic acid SolventB:MeCN+0.1% formic acid;Gradient:0min.2%B;0.2min.2%B;2.0min.98%B;2.2min.98%B.;2.21min.2%B;2.5min.2%B;200-300nm

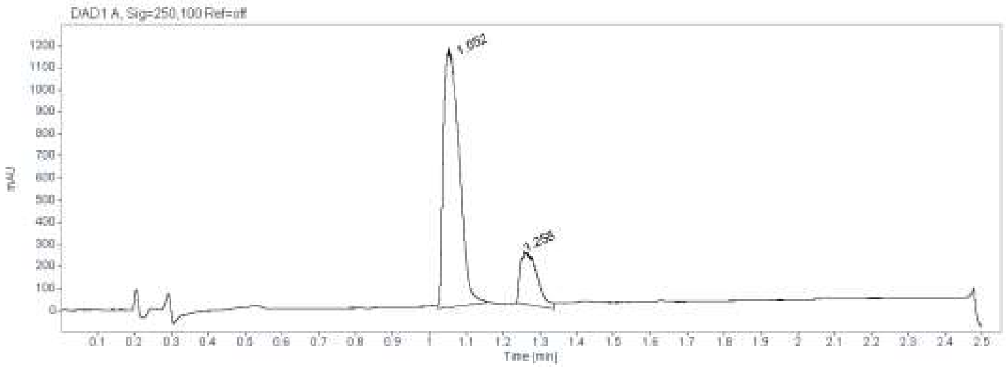

MSD1 SPC, MM-ES+APCI

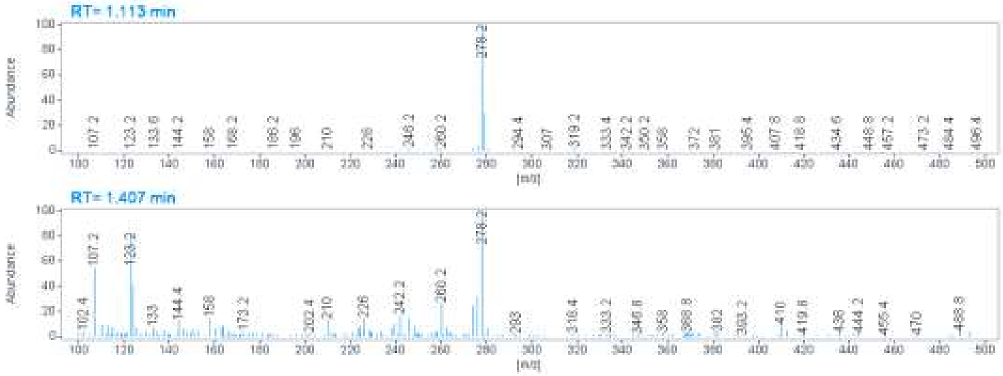

Signal: DAD1 A, Sig=250,100 Ref=off

RT [min]	Peak Area Percent
1.052	82.23
1.258	17.77

Compound **3ab**

Sample name: FHE37768_4OpF1

Injection date: 3/10/2021 1:29:27 PM

Analysis method: TAROSFASTSTANDARD_basic.M

Acq. operator: chemist

Injection volume: 2.000

Instrument: uHPLC Agilent 1290 Infinity

Description: Column: ACQUITY UHPLC BEH C18 (1.7µm) 2.1mmx50mm T30°C Detection: DAD + 6120 Quadrupole Flow: 1.0mL/min. SolventA: Water+0.005 NH4HCO3 SolventB:MeCN;Gradient:0min.2%B;0.2min.2%B;2.0min.98%B;2.2min.98%B., 2.21min.2%B;2.5min.2%B;200-300nm

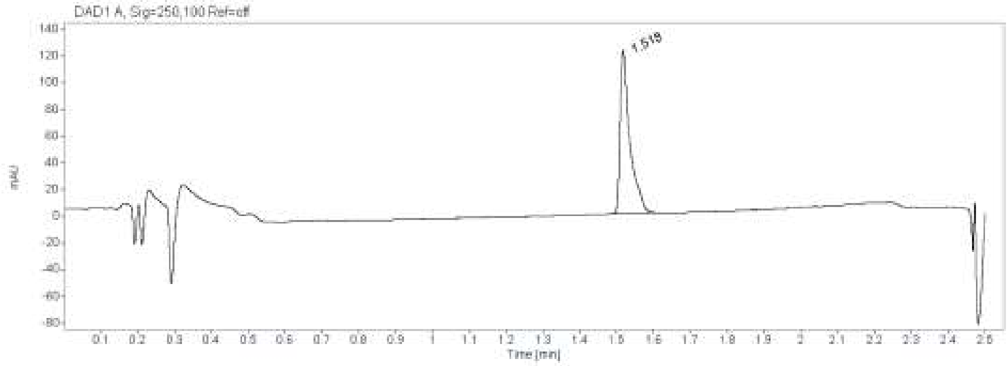

MSD1 SPC, MM-ES+APCI

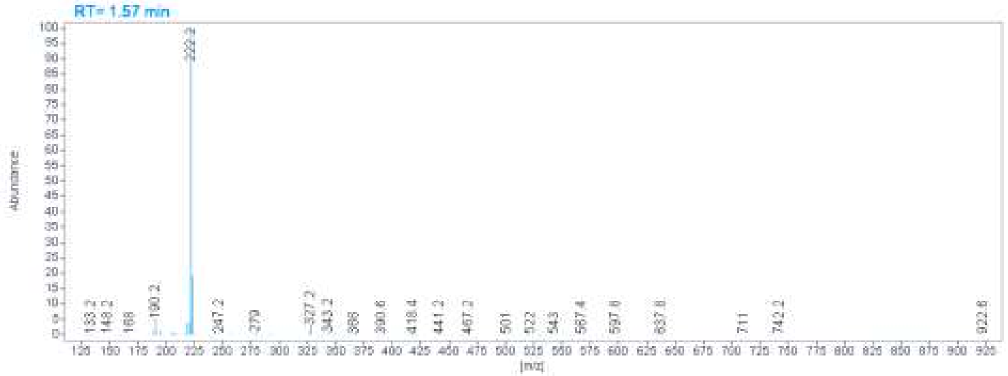

Signal: DAD1 A, Sig=250,100 Ref=off

RT [min]	Peak Area Percent
1.518	100.00

Compound **3ac**

**Sample name:**	FHE37769_9OpF1	**Injection date:**	3/10/2021 2:09:20 PM
**Analysis method:**	TAROSFASTSTANDARD_basic .M	**Acq. operator:**	chemist
**Injection volume:**	2.000	**Instrument:**	uHPLC Agilent 1290 Infinity

Description: Column: ACQUITY UHPLC BEH C18 (1.7µm) 2.1mmx50mm T30°C Detection: DAD + 6120 Quadrupole Flow: 1.0mL/min. SolventA: Water+0.005 NH4HCO3 SolventB:MeCN;Gradient:0min.2%B;0.2min.2%B;2.0min.98%B;2.2min.98%B., 2.21min.2% B;2.5min.2%B;200-300nm

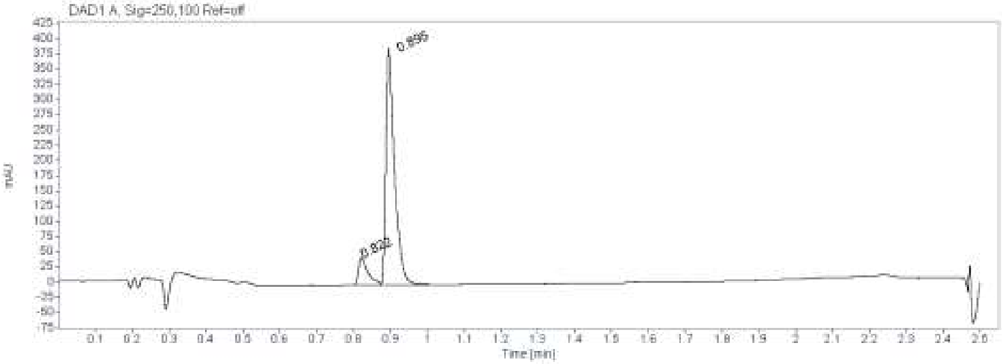

MSD1 SPC, MM-ES+APCI

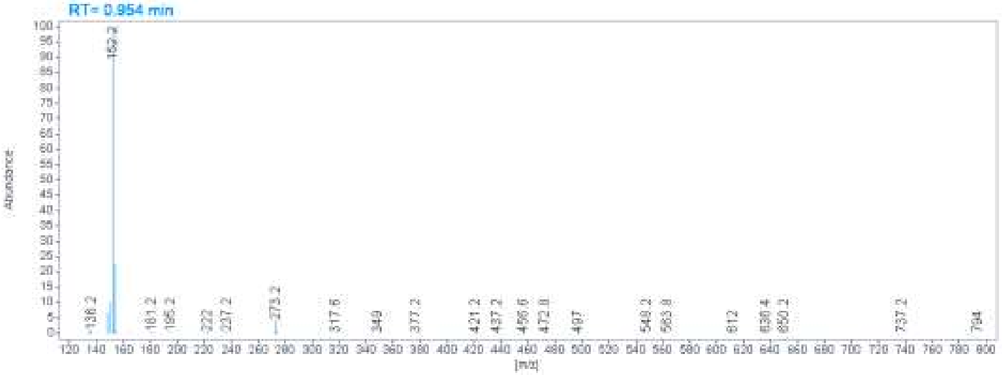

Signal: DAD1 A, Sig=250,100 Ref=off

RT [min]	Peak Area Percent
0.822	11.25
0.895	88.75

Racemic Samples in Chiral HPLC

Rac-3aa

===

Acq. Operator : SYSTEM Seq. Line : 1
Acq. Instrument : HPLC Location : Vial 32
Injection Date : 2/15/2021 12:19:27 PM Inj : 1
Inj Volume : 10.000 µl
Different Inj Volume from Sample Entry! Actual Inj Volume : 4.000 µl
Acq. Method : C:\CHEM32\1\DATA\P15036\P15036 2021-02-15 12-17-59\P15036_CHIRALPACK_IA_90-10.M
Last changed : 2/15/2021 12:17:59 PM by SYSTEM
Analysis Method : C:\CHEM32\1\DATA\P15036\P15036 2021-02-15 12-17-59\P15036_CHIRALPACK_IA_90-10.M (Sequence Method)
Last changed : 2/15/2021 1:30:28 PM by SYSTEM
(modified after loading)
Additional Info : Peak(s) manually integrated

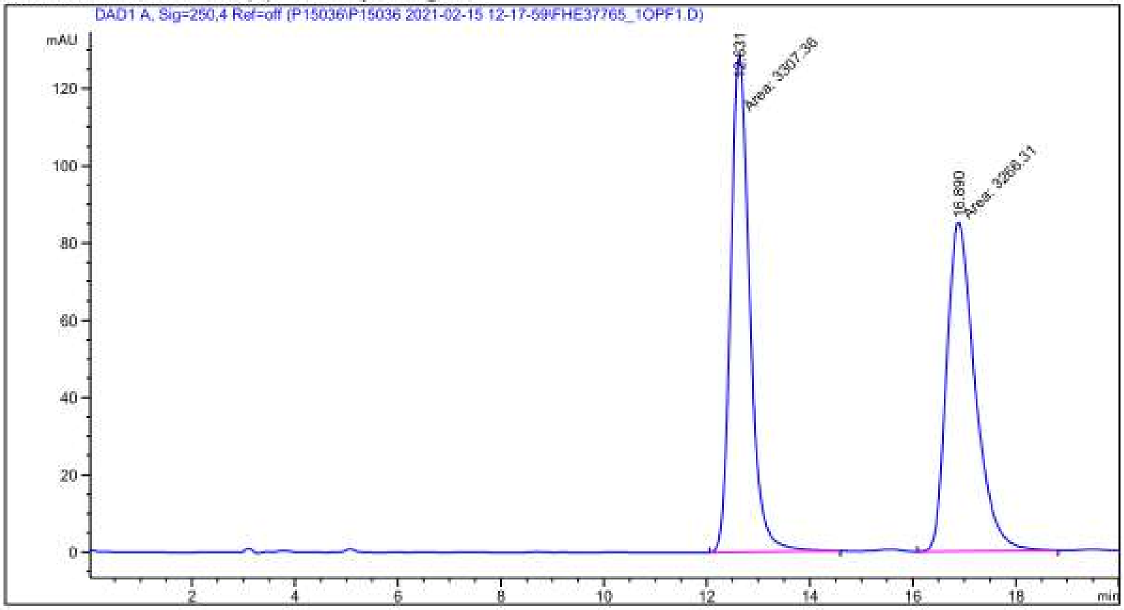

===
Area Percent Report
===

Sorted By : Signal
Multiplier : 1.0000
Dilution : 1.0000
Use Multiplier & Dilution Factor with ISTDs

Signal 1: DAD1 A, Sig=250,4 Ref=off

Peak #	RetTime [min]	Type	Width [min]	Area [mAU*s]	Height [mAU]	Area %
1	12.631	MM	0.4291	3307.35693	128.44942	50.3122
2	16.890	MM	0.6380	3266.31494	85.32535	49.6878

Rac-3ba

```
=====================================================================
Acq. Operator   : SYSTEM                         Seq. Line :   2
Acq. Instrument : HPLC                            Location : Vial 13
Injection Date  : 3/22/2021 1:11:42 PM                 Inj :   2
                                                Inj Volume : 10.000 µl
Method          : C:\CHEM32\1\DATA\P15036\P15036 2021-03-22 11-46-46\P15036_LUX_AMYLOSE-1_95-
                  5_HEX-ETOH.M (Sequence Method)
Last changed    : 3/22/2021 11:46:47 AM by SYSTEM
Additional Info : Peak(s) manually integrated
```

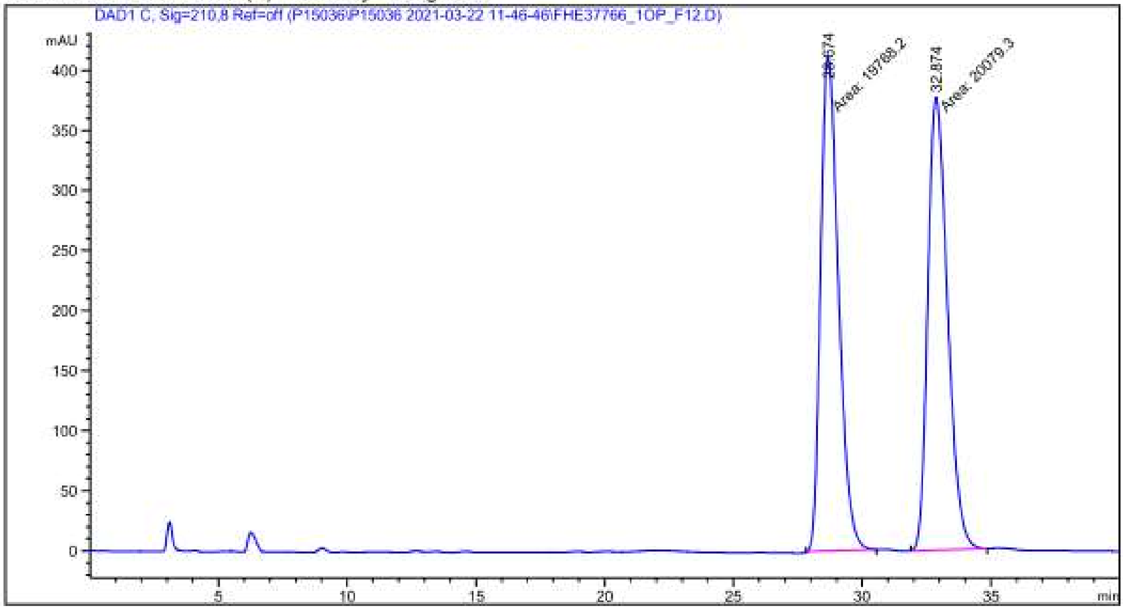


```
=====================================================================
                         Area Percent Report
=====================================================================

Sorted By             :      Signal
Multiplier            :      1.0000
Dilution              :      1.0000
Use Multiplier & Dilution Factor with ISTDs


Signal 1: DAD1 C, Sig=210,8 Ref=off

Peak RetTime Type  Width     Area      Height     Area
  #   [min]        [min]   [mAU*s]     [mAU]        %
----|-------|----|-------|----------|----------|--------|
   1  28.674 MM    0.8013 1.97682e4  411.14981  49.6097
   2  32.874 MM    0.8870 2.00793e4  377.29437  50.3903

Totals :                  3.98475e4  788.44418


=====================================================================
                          *** End of Report ***
```

Rac-3ca

```
=====================================================================
Acq. Operator   : SYSTEM                        Seq. Line :   4
Acq. Instrument : HPLC                           Location : Vial 41
Injection Date  : 2/24/2021 4:41:59 PM                Inj :   1
                                               Inj Volume : 10.000 µl
Acq. Method     : C:\CHEM32\1\DATA\P15036\P15036 2021-02-24 14-25-30\P15036_LUX_AMYLOSE-1_95-
                  5.M
Last changed    : 2/24/2021 3:57:46 PM by SYSTEM
Analysis Method : C:\CHEM32\1\DATA\P15036\P15036 2021-02-24 14-25-30\P15036_LUX_AMYLOSE-1_95-
                  5.M (Sequence Method)
Last changed    : 2/25/2021 7:51:09 AM by SYSTEM
                  (modified after loading)
Additional Info : Peak(s) manually integrated
```

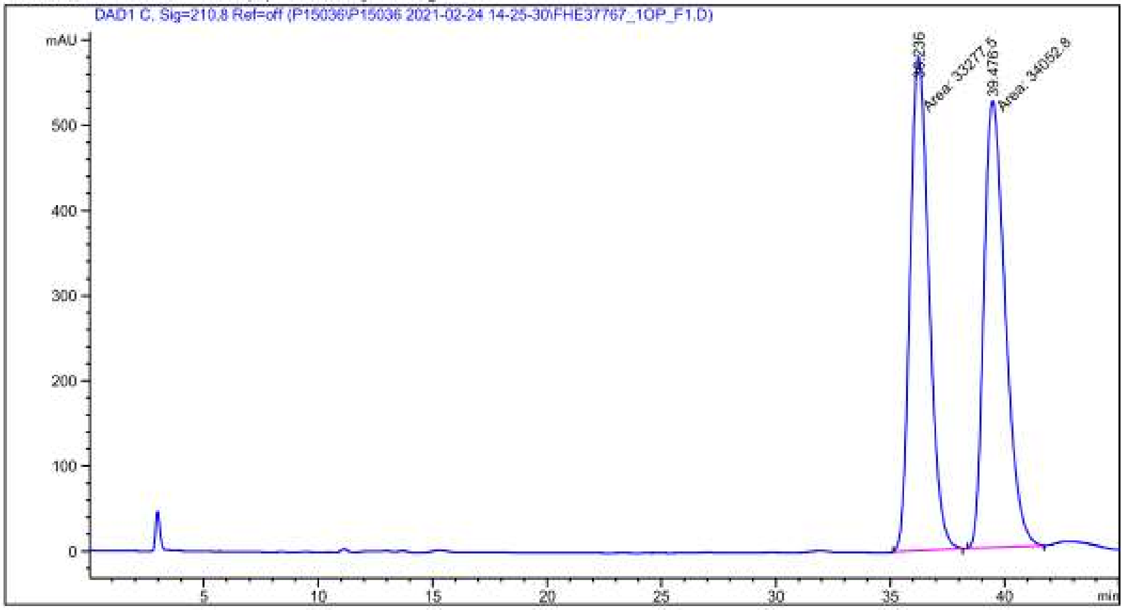


```
=====================================================================
                         Area Percent Report
=====================================================================

Sorted By             :      Signal
Multiplier            :      1.0000
Dilution              :      1.0000
Use Multiplier & Dilution Factor with ISTDs


Signal 1: DAD1 C, Sig=210,8 Ref=off

Peak RetTime Type  Width     Area      Height     Area
  #   [min]         [min]   [mAU*s]     [mAU]       %
----|-------|----|-------|----------|----------|--------|
   1  36.236 MM     0.9561 3.32775e4  580.09332  49.4242
   2  39.476 MM     1.0832 3.40528e4  523.96460  50.5758

Totals :                   6.73302e4  1104.05792
```

Rac-3ab

```
=====================================================================
Acq. Operator   : SYSTEM                        Seq. Line :   1
Acq. Instrument : HPLC                           Location : Vial 93
Injection Date  : 4/12/2021 3:09:13 PM                Inj :   1
                                               Inj Volume : 10.000 µl
Method          : C:\CHEM32\1\DATA\P15036\P15036 2021-04-12 15-07-42\P15036_CHIRALPAK_AS-3_98
                  -2_N-HEXANE-IPA.M (Sequence Method)
Last changed    : 4/12/2021 3:07:42 PM by SYSTEM
Additional Info : Peak(s) manually integrated
```

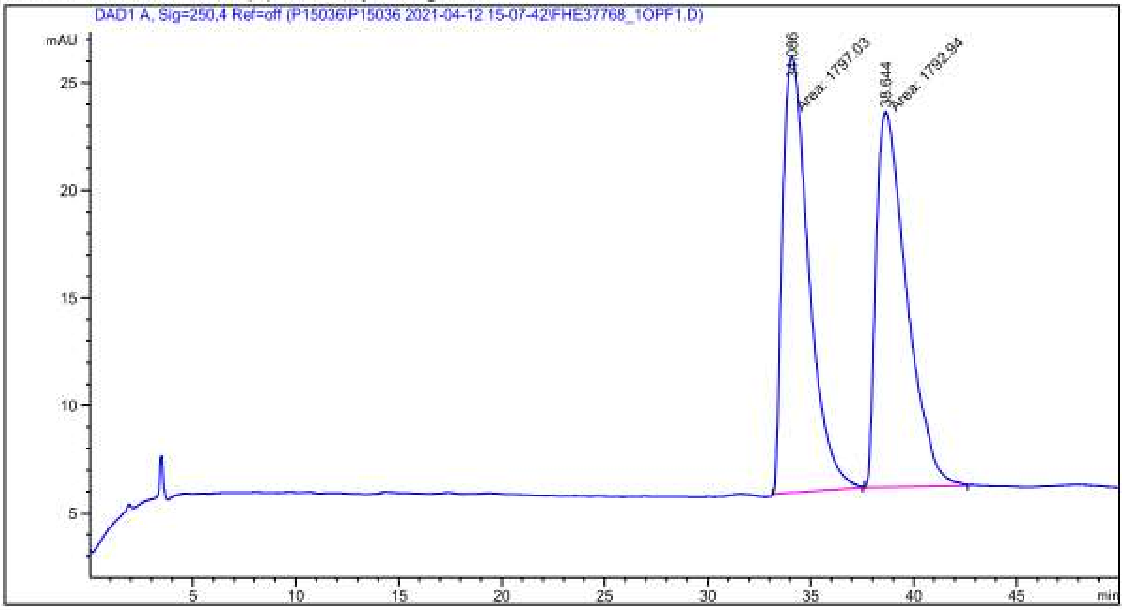


```
=====================================================================
                         Area Percent Report
=====================================================================

Sorted By             :      Signal
Multiplier            :      1.0000
Dilution              :      1.0000
Use Multiplier & Dilution Factor with ISTDs


Signal 1: DAD1 A, Sig=250,4 Ref=off

Peak RetTime Type  Width     Area      Height     Area
  #   [min]        [min]   [mAU*s]     [mAU]        %
----|-------|----|-------|----------|----------|--------|
   1  34.086 MM    1.4777 1797.03284   20.26788  50.0570
   2  38.644 MM    1.7135 1792.93945   17.43956  49.9430

Totals :                  3589.97229   37.70744


=====================================================================
                          *** End of Report ***
```

Rac-3ac

```
=======================================================================
Acq. Operator   : SYSTEM                          Seq. Line :   2
Acq. Instrument : HPLC                             Location : Vial 71
Injection Date  : 4/9/2021 12:07:49 PM                  Inj :   2
                                                Inj Volume : 10.000 µl
Different Inj Volume from Sample Entry!  Actual Inj Volume : 5.000 µl
Acq. Method     : C:\CHEM32\1\DATA\P15036\P15036 2021-04-09 11-23-13\P15036_CHIRALPAK_AS-3_90
                  -10_N-HEXANE-IPA.M
Last changed    : 4/9/2021 11:41:57 AM by SYSTEM
Analysis Method : C:\CHEM32\1\DATA\P15036\P15036 2021-04-09 11-23-13\P15036_CHIRALPAK_AS-3_90
                  -10_N-HEXANE-IPA.M (Sequence Method)
Last changed    : 4/9/2021 1:26:07 PM by SYSTEM
Additional Info : Peak(s) manually integrated
```

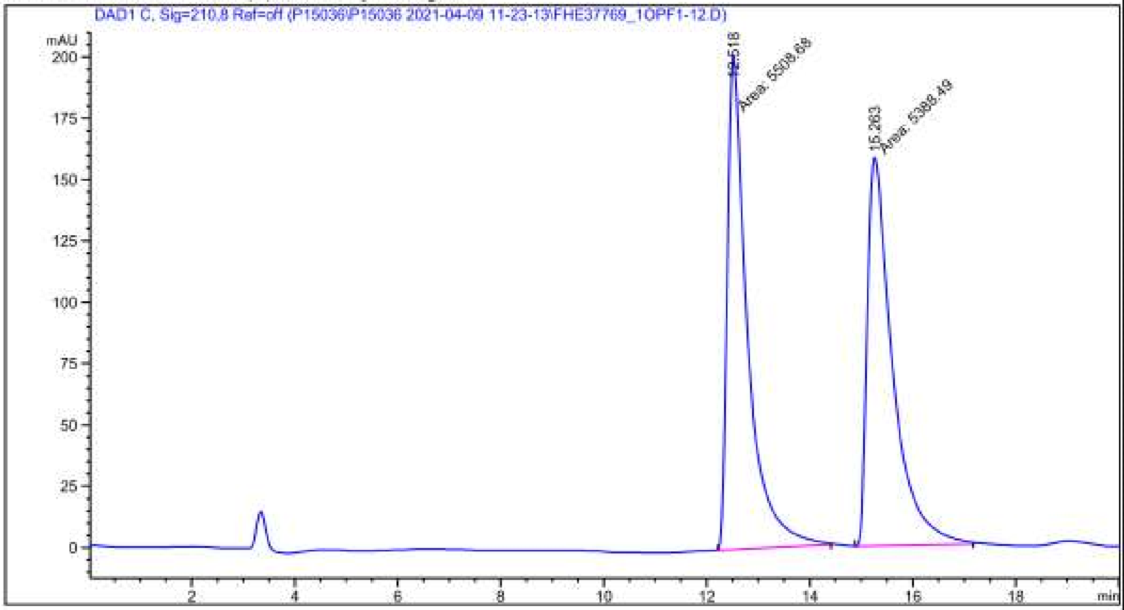


```
=======================================================================
                         Area Percent Report
=======================================================================

Sorted By             :      Signal
Multiplier            :      1.0000
Dilution              :      1.0000
Use Multiplier & Dilution Factor with ISTDs


Signal 1: DAD1 C, Sig=210,8 Ref=off

Peak RetTime Type  Width     Area      Height     Area
  #   [min]        [min]   [mAU*s]     [mAU]        %
----|-------|----|-------|----------|----------|--------|
   1  12.518 MM    0.4546 5508.67969  201.96335  50.5515
   2  15.263 MM    0.5660 5388.48926  158.67679  49.4485

Totals :                  1.08972e4   360.64014
```

Chiral Samples

Table S2

Table S2 Entry 1

```
=====================================================================
Acq. Operator   : SYSTEM                        Seq. Line :   2
Acq. Instrument : HPLC                           Location : Vial 33
Injection Date  : 2/15/2021 12:41:02 PM               Inj :   1
                                               Inj Volume : 10.000 µl
Different Inj Volume from Sample Entry!  Actual Inj Volume : 2.000 µl
Acq. Method     : C:\CHEM32\1\DATA\P15036\P15036 2021-02-15 12-17-59\P15036_CHIRALPACK_IA_90-
                  10.M
Last changed    : 2/15/2021 12:17:59 PM by SYSTEM
Analysis Method : C:\CHEM32\1\DATA\P15036\P15036 2021-02-15 12-17-59\P15036_CHIRALPACK_IA_90-
                  10.M (Sequence Method)
Last changed    : 2/15/2021 1:30:28 PM by SYSTEM
                  (modified after loading)
Additional Info : Peak(s) manually integrated
```

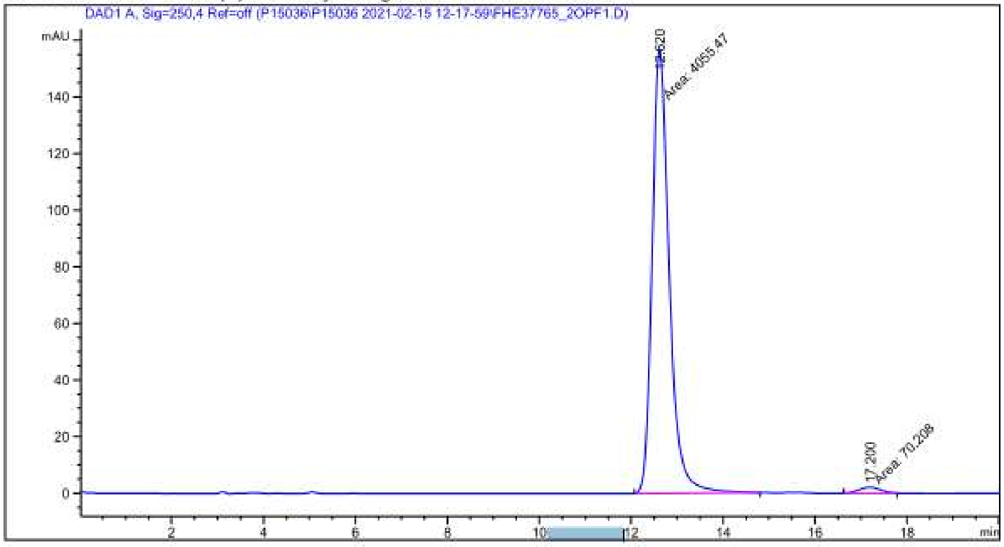


```
=====================================================================
                         Area Percent Report
=====================================================================

Sorted By             :      Signal
Multiplier            :      1.0000
Dilution              :      1.0000
Use Multiplier & Dilution Factor with ISTDs


Signal 1: DAD1 A, Sig=250,4 Ref=off

Peak RetTime Type  Width     Area      Height     Area
  #   [min]        [min]   [mAU*s]     [mAU]        %
----|-------|----|-------|----------|----------|--------|
   1  12.620 MM    0.4311 4055.46606  156.80089  98.2983
   2  17.200 MM    0.5444   70.20801    2.14956   1.7017

Totals :                  4125.67407  158.95045
```

Table S2 Entry 2

```
=====================================================================
Acq. Operator   : SYSTEM                        Seq. Line :   2
Acq. Instrument : HPLC                           Location : Vial 35
Injection Date  : 2/24/2021 1:31:31 PM                Inj :   1
                                               Inj Volume : 10.000 µl
Method          : C:\CHEM32\1\DATA\P15036\P15036 2021-02-24 12-58-15\P15036_LUX_AMYLOSE-1_95-
                  5.M (Sequence Method)
Last changed    : 2/24/2021 12:58:16 PM by SYSTEM
Additional Info : Peak(s) manually integrated
```

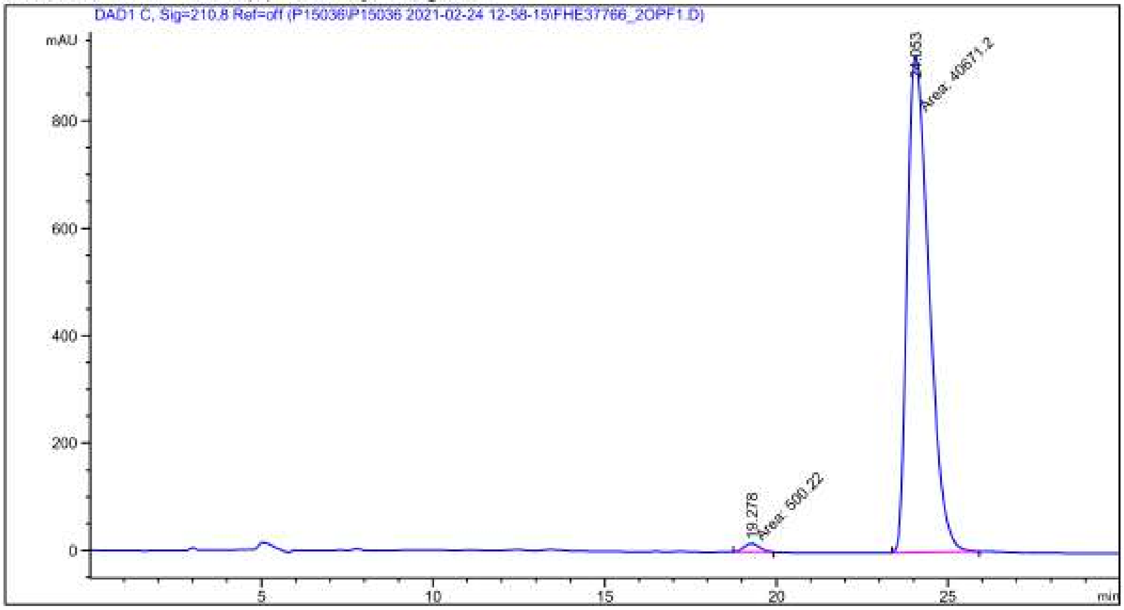


```
=====================================================================
                         Area Percent Report
=====================================================================

Sorted By             :      Signal
Multiplier            :      1.0000
Dilution              :      1.0000
Use Multiplier & Dilution Factor with ISTDs


Signal 1: DAD1 C, Sig=210,8 Ref=off

Peak RetTime Type  Width     Area      Height     Area
  #   [min]        [min]   [mAU*s]     [mAU]        %
----|-------|----|-------|----------|----------|--------|
   1  19.278 MM    0.5265  500.22034   15.83353   1.2150
   2  24.053 MM    0.7336 4.06712e4  924.05859  98.7850

Totals :                  4.11714e4  939.89212


=====================================================================
                         *** End of Report ***
```

Table S2 Entry 3

```
========================================================================
Acq. Operator   : SYSTEM                         Seq. Line :    5
Acq. Instrument : HPLC                            Location : Vial 43
Injection Date  : 2/24/2021 5:28:40 PM                 Inj :    1
                                                Inj Volume : 10.000 µl
Acq. Method     : C:\CHEM32\1\DATA\P15036\P15036 2021-02-24 14-25-30\P15036_LUX_AMYLOSE-1_95-
                  5.M
Last changed    : 2/24/2021 3:57:46 PM by SYSTEM
Analysis Method : C:\CHEM32\1\DATA\P15036\P15036 2021-02-24 14-25-30\P15036_LUX_AMYLOSE-1_95-
                  5.M (Sequence Method)
Last changed    : 2/25/2021 7:51:09 AM by SYSTEM
                  (modified after loading)
Additional Info : Peak(s) manually integrated
```

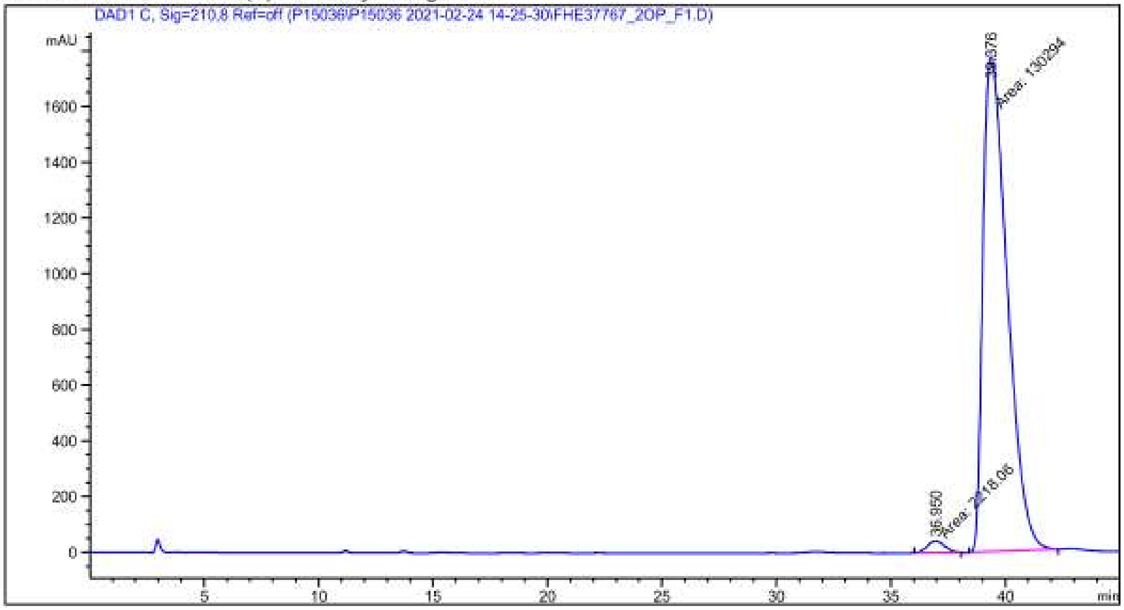


```
========================================================================
                       Area Percent Report
========================================================================

Sorted By             :      Signal
Multiplier            :      1.0000
Dilution              :      1.0000
Use Multiplier & Dilution Factor with ISTDs


Signal 1: DAD1 C, Sig=210,8 Ref=off

Peak RetTime Type  Width     Area      Height     Area
  #   [min]        [min]   [mAU*s]     [mAU]       %
----|-------|----|-------|----------|----------|--------|
   1  36.950 MM    0.8810 2218.06152   41.96149   1.6739
   2  39.376 MM    1.2228 1.30294e5  1775.93982  98.3261

Totals :                  1.32513e5  1817.90131
```

Table S2 Entry 4

```
=====================================================================
Acq. Operator   : SYSTEM                        Seq. Line :   2
Acq. Instrument : HPLC                           Location : Vial 94
Injection Date  : 4/12/2021 4:00:48 PM                Inj :   1
                                               Inj Volume : 10.000 µl
Method          : C:\CHEM32\1\DATA\P15036\P15036 2021-04-12 15-07-42\P15036_CHIRALPAK_AS-3_98
                  -2_N-HEXANE-IPA.M (Sequence Method)
Last changed    : 4/12/2021 3:07:42 PM by SYSTEM
Additional Info : Peak(s) manually integrated
```

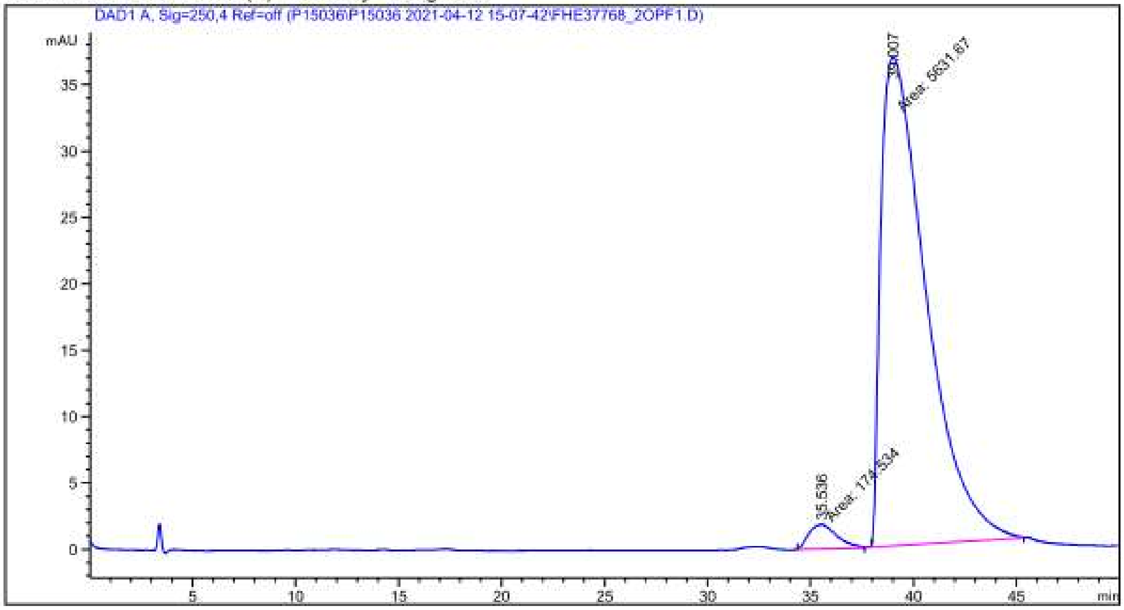


```
=====================================================================
                     Area Percent Report
=====================================================================

Sorted By             :      Signal
Multiplier            :      1.0000
Dilution              :      1.0000
Use Multiplier & Dilution Factor with ISTDs


Signal 1: DAD1 A, Sig=250,4 Ref=off

Peak RetTime Type  Width     Area      Height     Area
  #   [min]        [min]   [mAU*s]     [mAU]        %
----|-------|----|-------|----------|----------|--------|
   1  35.536 MM    1.5894  174.53391    1.83020   3.0060
   2  39.007 MM    2.5493 5631.66650   36.81835  96.9940

Totals :                  5806.20041   38.64855


=====================================================================
                     *** End of Report ***
```

Table S2 Entry 5

```
=====================================================================
Acq. Operator   : SYSTEM                          Seq. Line :   1
Acq. Instrument : HPLC                             Location : Vial 81
Injection Date  : 4/9/2021 1:04:27 PM                   Inj :   1
                                                 Inj Volume : 10.000 µl
Different Inj Volume from Sample Entry!  Actual Inj Volume : 5.000 µl
Acq. Method     : C:\CHEM32\1\DATA\P15036\P15036-2021-04-09 2021-04-09 13-01-37\P15036_
                  CHIRALPAK_AS-3_90-10_N-HEXANE-IPA.M
Last changed    : 4/9/2021 1:16:35 PM by SYSTEM
                  (modified after loading)
Analysis Method : C:\CHEM32\1\DATA\P15036\P15036-2021-04-09 2021-04-09 13-01-37\P15036_
                  CHIRALPAK_AS-3_90-10_N-HEXANE-IPA.M (Sequence Method)
Last changed    : 4/9/2021 1:33:23 PM by SYSTEM
                  (modified after loading)
Additional Info : Peak(s) manually integrated
```

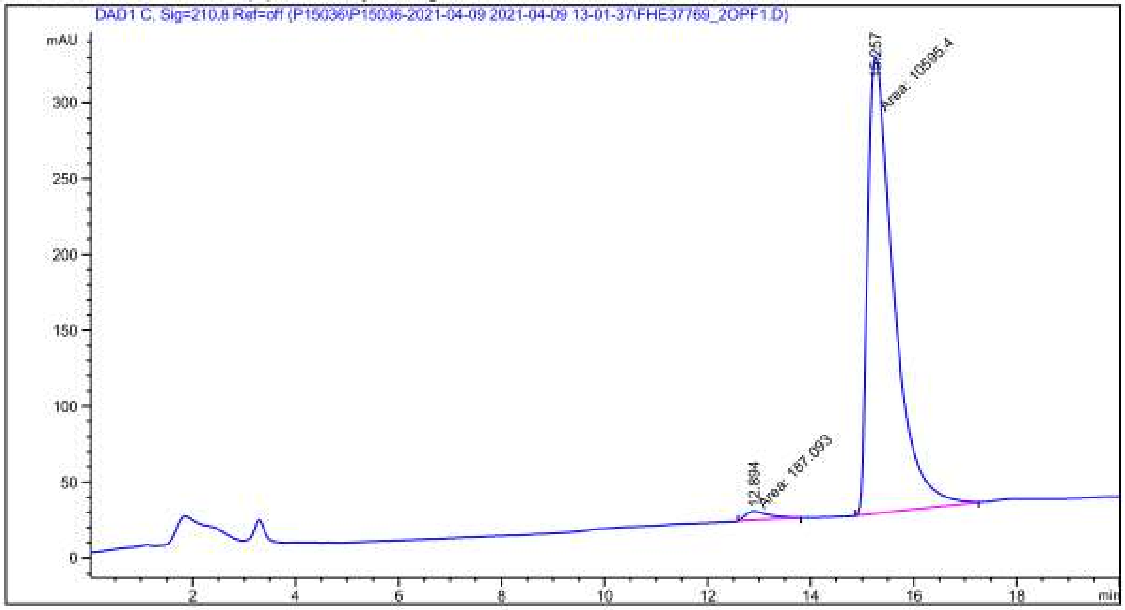


```
=====================================================================
                         Area Percent Report
=====================================================================

Sorted By             :      Signal
Multiplier            :      1.0000
Dilution              :      1.0000
Use Multiplier & Dilution Factor with ISTDs


Signal 1: DAD1 C, Sig=210,8 Ref=off

Peak RetTime Type  Width     Area      Height     Area
  #   [min]        [min]   [mAU*s]     [mAU]        %
----|-------|----|-------|----------|----------|--------|
   1  12.894 MM    0.5348  187.09317    5.83090   1.7352
   2  15.257 MM    0.5852 1.05954e4   301.75394  98.2648
```

Table S3

Table S3 Entry 1

```
=====================================================================
Acq. Operator   : SYSTEM                        Seq. Line :   2
Acq. Instrument : HPLC                           Location : Vial 22
Injection Date  : 3/18/2021 2:24:27 PM                Inj :   1
                                               Inj Volume : 10.000 µl
Different Inj Volume from Sample Entry!  Actual Inj Volume : 5.000 µl
Method          : C:\CHEM32\1\DATA\P15036\P15036 2021-03-18 13-51-23\P15036_CHIRALPACK_IA_90-
                  10.M (Sequence Method)
Last changed    : 3/18/2021 1:51:23 PM by SYSTEM
Additional Info : Peak(s) manually integrated
```

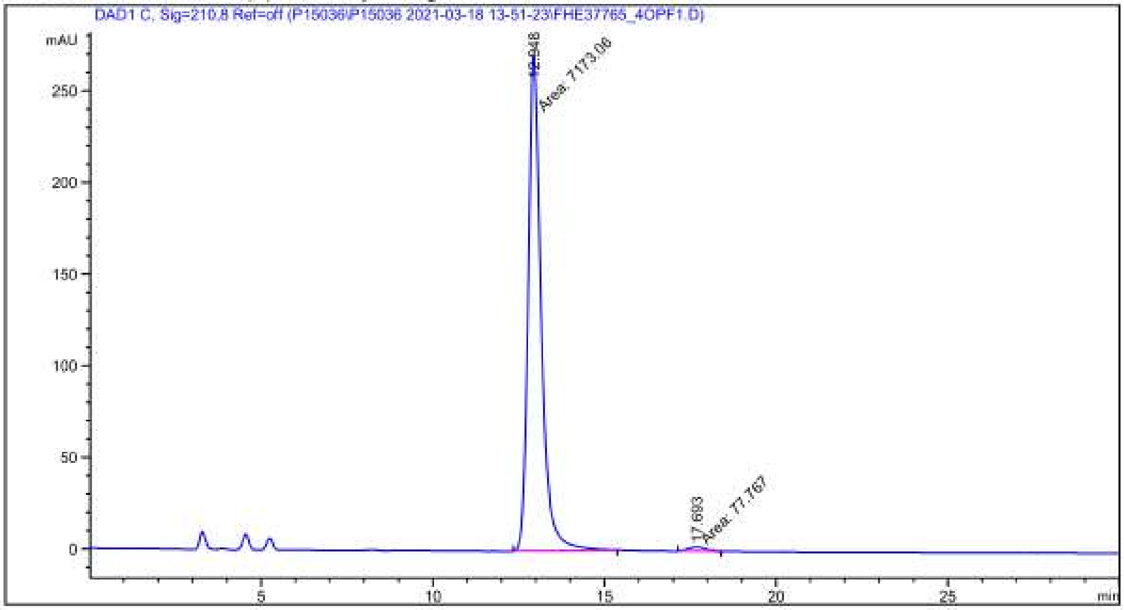


```
=====================================================================
                         Area Percent Report
=====================================================================

Sorted By             :      Signal
Multiplier            :      1.0000
Dilution              :      1.0000
Use Multiplier & Dilution Factor with ISTDs


Signal 1: DAD1 C, Sig=210,8 Ref=off

Peak RetTime Type  Width     Area      Height     Area
  #   [min]        [min]   [mAU*s]     [mAU]       %
----|-------|----|-------|----------|----------|--------|
   1  12.948 MM    0.4427 7173.05566  270.02209  98.9275
   2  17.693 MM    0.5788   77.76697    2.23934   1.0725

Totals :                  7250.82263  272.26143


=====================================================================
                          *** End of Report ***
```

Table S3 Entry 2

```
=====================================================================
Acq. Operator   : SYSTEM                        Seq. Line :   3
Acq. Instrument : HPLC                           Location : Vial 14
Injection Date  : 3/22/2021 2:02:26 PM                Inj :   1
                                               Inj Volume : 10.000 µl
Method          : C:\CHEM32\1\DATA\P15036\P15036 2021-03-22 11-46-46\P15036_LUX_AMYLOSE-1_95-
                  5_HEX-ETOH.M (Sequence Method)
Last changed    : 3/22/2021 11:46:47 AM by SYSTEM
Additional Info : Peak(s) manually integrated
```

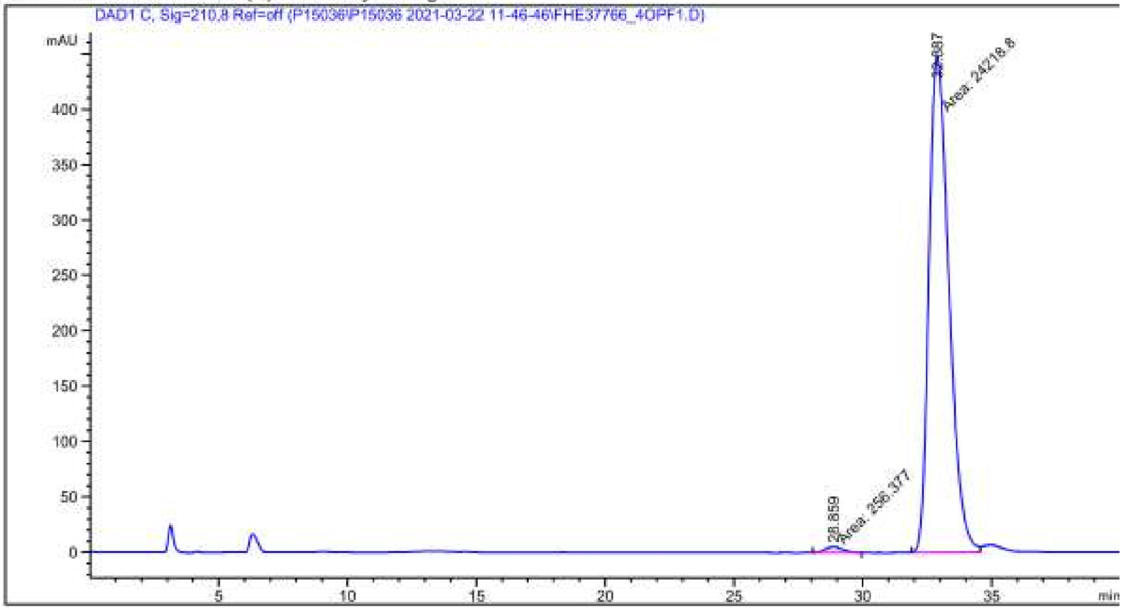


```
=====================================================================
                         Area Percent Report
=====================================================================

Sorted By             :      Signal
Multiplier            :      1.0000
Dilution              :      1.0000
Use Multiplier & Dilution Factor with ISTDs


Signal 1: DAD1 C, Sig=210,8 Ref=off

Peak RetTime Type  Width     Area      Height     Area
  #   [min]        [min]   [mAU*s]     [mAU]        %
----|-------|----|-------|----------|----------|--------|
   1  28.859 MM    0.7835  256.37653    5.45384   1.0475
   2  32.887 MF    0.9024 2.42188e4  447.32291  98.9525

Totals :                  2.44752e4  452.77675


=====================================================================
                          *** End of Report ***
```

Table S3 Entry 3

```
=====================================================================
Acq. Operator   : SYSTEM                        Seq. Line :   2
Acq. Instrument : HPLC                           Location : Vial 92
Injection Date  : 3/23/2021 3:54:23 PM                Inj :   1
                                               Inj Volume : 10.000 µl
Different Inj Volume from Sample Entry!  Actual Inj Volume : 1.000 µl
Method          : C:\CHEM32\1\DATA\P15036\P15036 2021-03-23 14-56-24\P15036_LUX_AMYLOSE-1_95-
                  5_HEX-ETOH.M (Sequence Method)
Last changed    : 3/23/2021 2:56:24 PM by SYSTEM
Additional Info : Peak(s) manually integrated
```

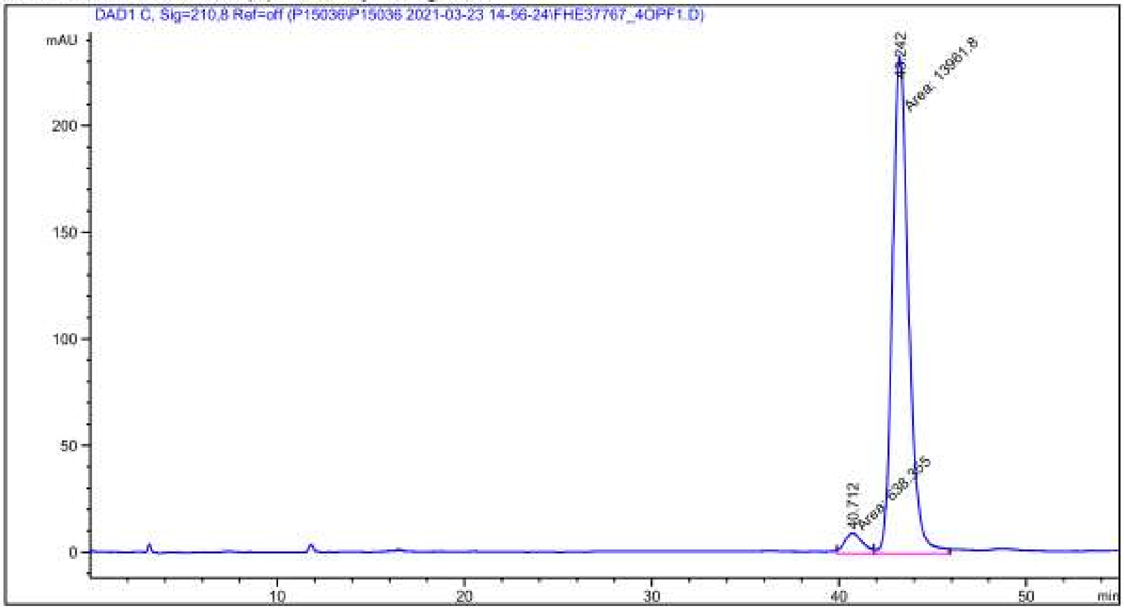


```
=====================================================================
                         Area Percent Report
=====================================================================

Sorted By             :      Signal
Multiplier            :      1.0000
Dilution              :      1.0000
Use Multiplier & Dilution Factor with ISTDs


Signal 1: DAD1 C, Sig=210,8 Ref=off
```

Peak #	RetTime [min]	Type	Width [min]	Area [mAU*s]	Height [mAU]	Area %
1	40.712	MF	1.1257	638.35455	9.45161	4.3723
2	43.242	FM	0.9985	1.39618e4	233.03938	95.6277

Totals : 1.46001e4 242.49099

```
=====================================================================
                         *** End of Report ***
```

Table S3 Entry 4

===

Acq. Operator : SYSTEM Seq. Line : 3
Acq. Instrument : HPLC Location : Vial 95
Injection Date : 4/12/2021 4:52:23 PM Inj : 1
Inj Volume : 10.000 µl
Method : C:\CHEM32\1\DATA\P15036\P15036 2021-04-12 15-07-42\P15036_CHIRALPAK_AS-3_98-2_N-HEXANE-IPA.M (Sequence Method)
Last changed : 4/12/2021 3:07:42 PM by SYSTEM
Additional Info : Peak(s) manually integrated

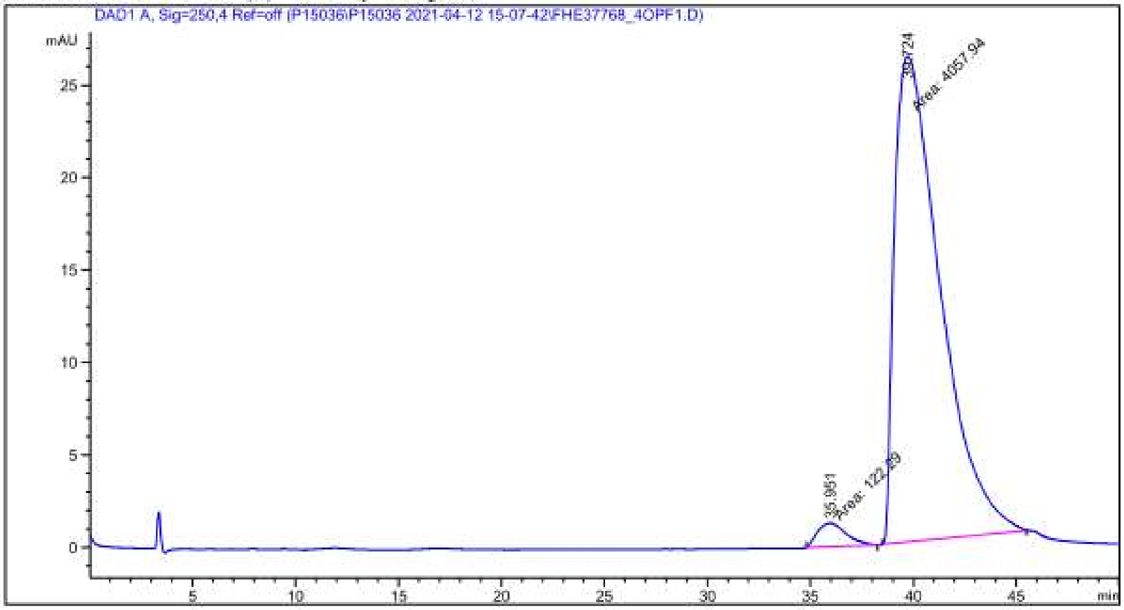

===

Area Percent Report

===

Sorted By : Signal
Multiplier : 1.0000
Dilution : 1.0000
Use Multiplier & Dilution Factor with ISTDs

Signal 1: DAD1 A, Sig=250,4 Ref=off

Peak #	RetTime [min]	Type	Width [min]	Area [mAU*s]	Height [mAU]	Area %
1	35.951	MM	1.6091	122.29038	1.26666	2.9254
2	39.724	MM	2.5787	4057.93799	26.22735	97.0746

Totals : 4180.22837 27.49401

===

*** End of Report ***

Table S3 Entry 5

```
=====================================================================
Acq. Operator   : SYSTEM                          Seq. Line :   1
Acq. Instrument : HPLC                             Location : Vial 91
Injection Date  : 4/12/2021 11:58:31 AM                 Inj :   1
                                                 Inj Volume : 10.000 µl
Method          : C:\CHEM32\1\DATA\P15036\P15036 2021-04-12 11-57-06\P15036_CHIRALPAK_AS-3_90
                  -10_N-HEXANE-IPA.M (Sequence Method)
Last changed    : 4/12/2021 11:57:06 AM by SYSTEM
Additional Info : Peak(s) manually integrated
```

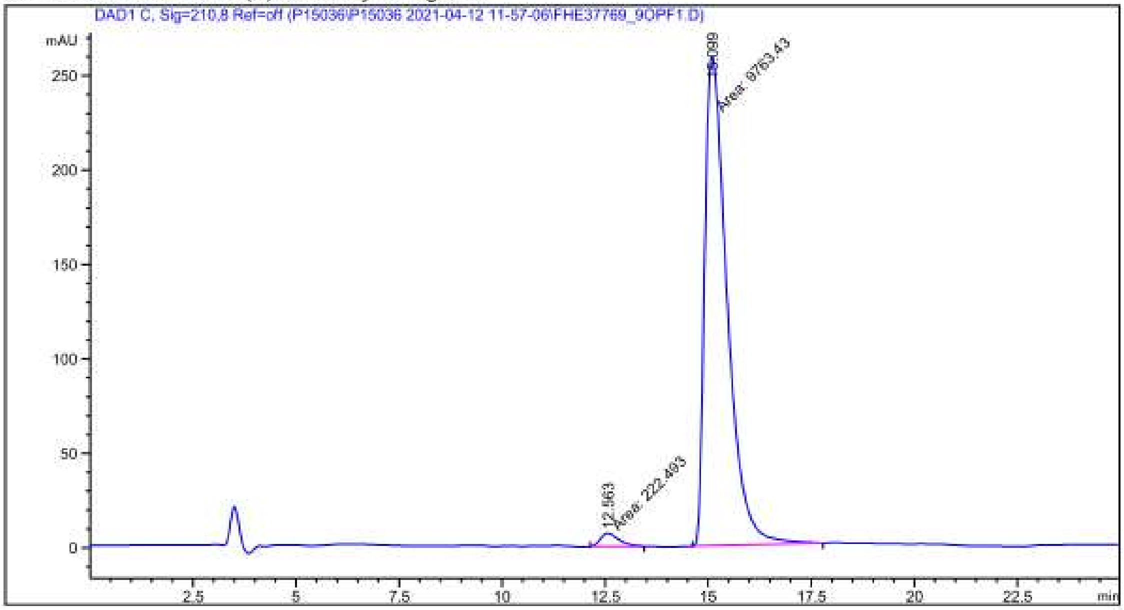


```
=====================================================================
                         Area Percent Report
=====================================================================

Sorted By             :      Signal
Multiplier            :      1.0000
Dilution              :      1.0000
Use Multiplier & Dilution Factor with ISTDs


Signal 1: DAD1 C, Sig=210,8 Ref=off

Peak RetTime Type  Width     Area      Height     Area
  #   [min]        [min]   [mAU*s]     [mAU]        %
----|-------|----|-------|----------|----------|--------|
   1  12.563 MM    0.5267  222.49327    7.04049   2.2281
   2  15.099 MM    0.6284 9763.42969  258.95157  97.7719

Totals :                  9985.92296  265.99205


=====================================================================
                          *** End of Report ***
```

Table 4

Table 4 Entry 1

```
=====================================================================
Acq. Operator   : SYSTEM                        Seq. Line :   1
Acq. Instrument : HPLC                           Location : Vial 42
Injection Date  : 4/8/2021 3:32:09 PM                 Inj :   1
                                               Inj Volume : 10.000 µl
Different Inj Volume from Sample Entry!  Actual Inj Volume : 5.000 µl
Acq. Method     : C:\CHEM32\1\DATA\P15036\P15036 2021-04-08 15-30-38\P15036_CHIRALPACK_IA_90-
                  10.M
Last changed    : 4/8/2021 3:47:31 PM by SYSTEM
                  (modified after loading)
Analysis Method : C:\CHEM32\1\DATA\P15036\P15036 2021-04-08 15-30-38\P15036_CHIRALPACK_IA_90-
                  10.M (Sequence Method)
Last changed    : 4/8/2021 3:30:38 PM by SYSTEM
Additional Info : Peak(s) manually integrated
```

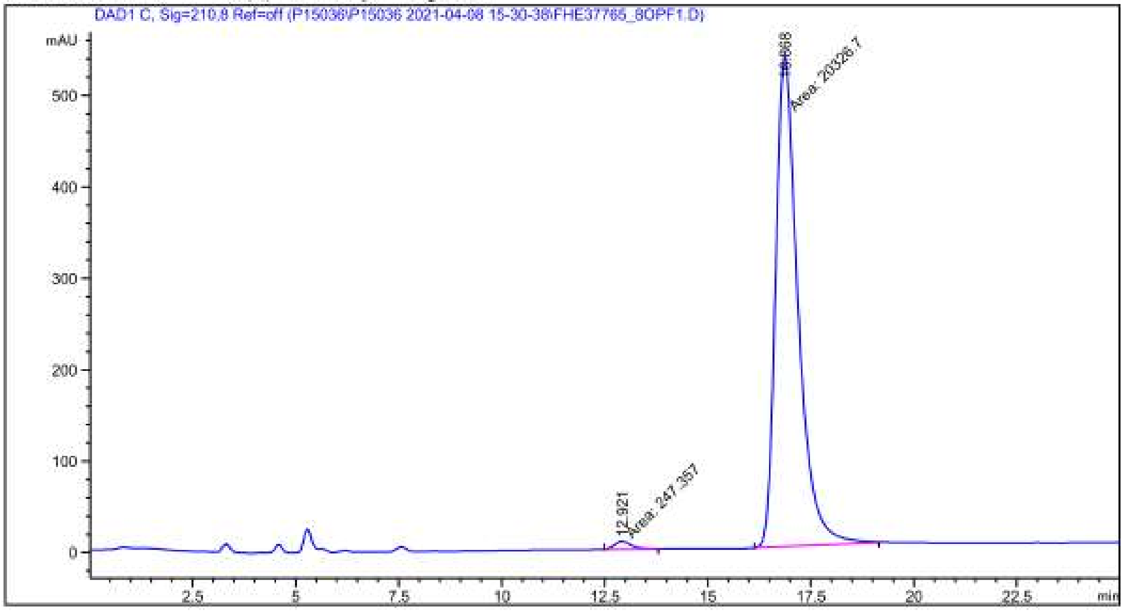


```
=====================================================================
                         Area Percent Report
=====================================================================

Sorted By             :      Signal
Multiplier            :      1.0000
Dilution              :      1.0000
Use Multiplier & Dilution Factor with ISTDs


Signal 1: DAD1 C, Sig=210,8 Ref=off

Peak RetTime Type  Width     Area      Height     Area
  #   [min]        [min]   [mAU*s]     [mAU]        %
----|-------|----|-------|----------|----------|--------|
   1  12.921 MM    0.4708  247.35719    8.75686   1.2023
   2  16.868 MM    0.6317 2.03267e4  536.30658  98.7977

Totals :                  2.05740e4  545.06344
```

Table 4 Entry 2

```
=====================================================================
Acq. Operator   : SYSTEM                         Seq. Line :   2
Acq. Instrument : HPLC                            Location : Vial 43
Injection Date  : 4/8/2021 3:58:59 PM                  Inj :   1
                                                Inj Volume : 10.000 µl
Different Inj Volume from Sample Entry!  Actual Inj Volume : 5.000 µl
Acq. Method     : C:\CHEM32\1\DATA\P15036\P15036 2021-04-08 15-30-38\P15036_CHIRALPACK_IA_90-
                  10.M
Last changed    : 4/8/2021 3:47:31 PM by SYSTEM
Analysis Method : C:\CHEM32\1\DATA\P15036\P15036 2021-04-08 15-30-38\P15036_CHIRALPACK_IA_90-
                  10.M (Sequence Method)
Last changed    : 4/8/2021 3:30:38 PM by SYSTEM
Additional Info : Peak(s) manually integrated
```

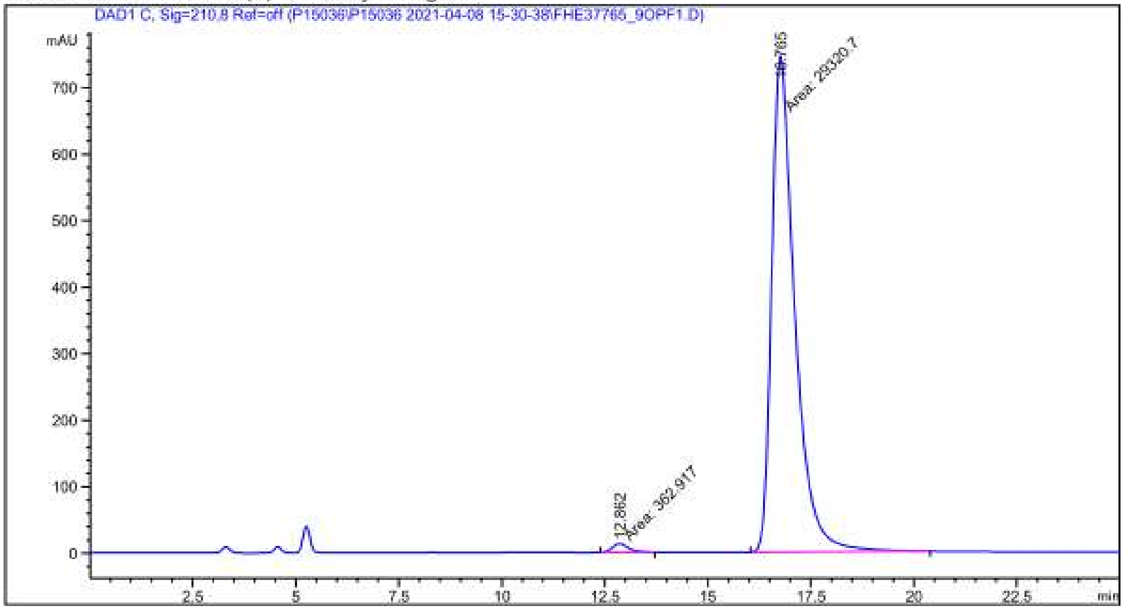


```
=====================================================================
                         Area Percent Report
=====================================================================

Sorted By             :      Signal
Multiplier            :      1.0000
Dilution              :      1.0000
Use Multiplier & Dilution Factor with ISTDs


Signal 1: DAD1 C, Sig=210,8 Ref=off

Peak RetTime Type  Width     Area      Height     Area
  #   [min]        [min]   [mAU*s]     [mAU]       %
----|-------|----|-------|----------|----------|--------|
   1  12.862 MM    0.4594  362.91724   13.16511   1.2226
   2  16.765 MM    0.6553 2.93207e4   745.67810  98.7774

Totals :                  2.96836e4   758.84321
```

Table 4 Entry 3

```
=====================================================================
Acq. Operator   : SYSTEM                         Seq. Line :   3
Acq. Instrument : HPLC                            Location : Vial 44
Injection Date  : 4/8/2021 4:25:39 PM                  Inj :   1
                                                Inj Volume : 10.000 µl
Different Inj Volume from Sample Entry!  Actual Inj Volume : 5.000 µl
Acq. Method     : C:\CHEM32\1\DATA\P15036\P15036 2021-04-08 15-30-38\P15036_CHIRALPACK_IA_90-
                  10.M
Last changed    : 4/8/2021 3:47:31 PM by SYSTEM
Analysis Method : C:\CHEM32\1\DATA\P15036\P15036 2021-04-08 15-30-38\P15036_CHIRALPACK_IA_90-
                  10.M (Sequence Method)
Last changed    : 4/8/2021 3:30:38 PM by SYSTEM
Additional Info : Peak(s) manually integrated
```

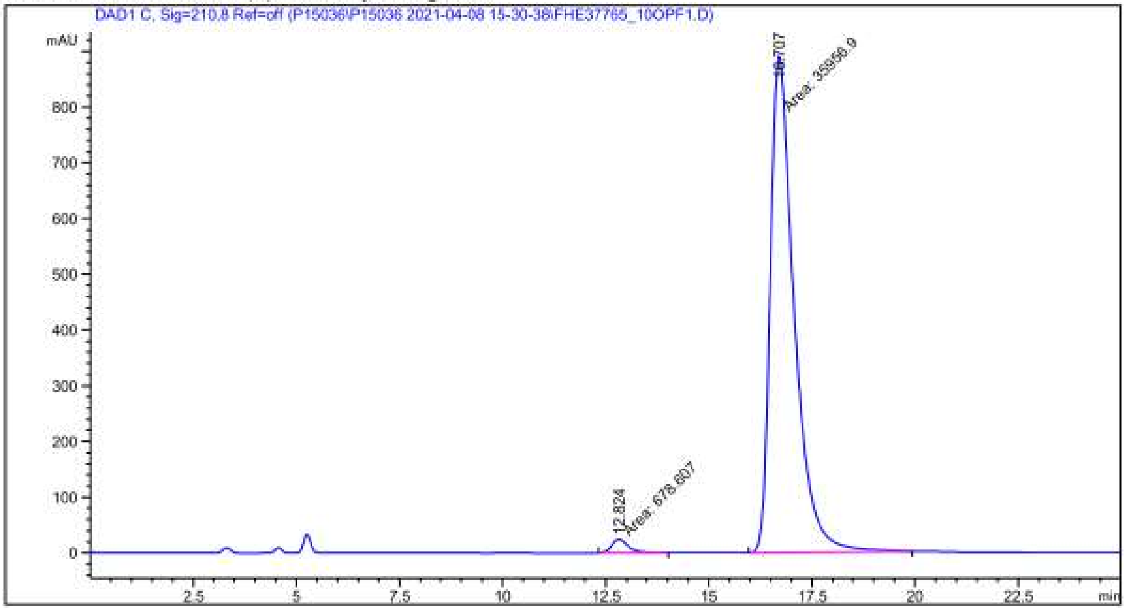


```
=====================================================================
                        Area Percent Report
=====================================================================

Sorted By             :      Signal
Multiplier            :      1.0000
Dilution              :      1.0000
Use Multiplier & Dilution Factor with ISTDs


Signal 1: DAD1 C, Sig=210,8 Ref=off

Peak RetTime Type  Width     Area      Height     Area
  #   [min]        [min]   [mAU*s]     [mAU]        %
----|-------|----|-------|----------|----------|--------|
   1  12.824 MM    0.4641  678.60748   24.37027   1.8523
   2  16.707 MM    0.6726 3.59569e4  890.98999  98.1477

Totals :                  3.66355e4  915.36026
```

Table 4 Entry 4

```
=====================================================================
Acq. Operator   : SYSTEM                        Seq. Line :   3
Acq. Instrument : HPLC                           Location : Vial 83
Injection Date  : 4/9/2021 1:47:31 PM                 Inj :   1
                                               Inj Volume : 10.000 µl
Different Inj Volume from Sample Entry!  Actual Inj Volume : 5.000 µl
Acq. Method     : C:\CHEM32\1\DATA\P15036\P15036-2021-04-09 2021-04-09 13-01-37\P15036_
                  CHIRALPAK_AS-3_90-10_N-HEXANE-IPA.M
Last changed    : 4/9/2021 1:16:35 PM by SYSTEM
Analysis Method : C:\CHEM32\1\DATA\P15036\P15036-2021-04-09 2021-04-09 13-01-37\P15036_
                  CHIRALPAK_AS-3_90-10_N-HEXANE-IPA.M (Sequence Method)
Last changed    : 4/9/2021 2:08:05 PM by SYSTEM
                  (modified after loading)
Additional Info : Peak(s) manually integrated
```

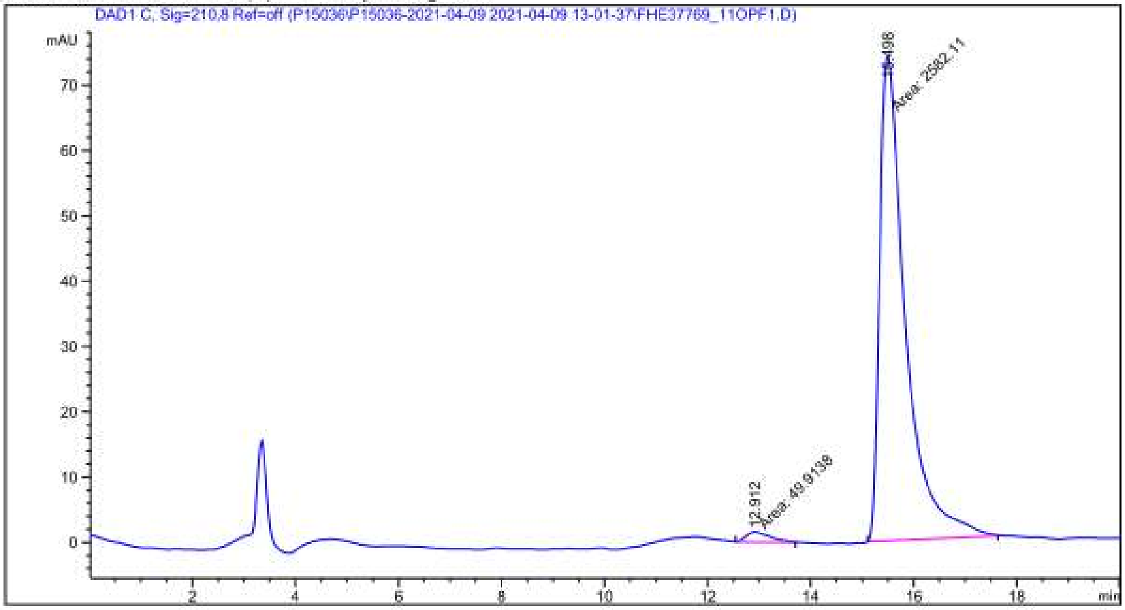


```
=====================================================================
                         Area Percent Report
=====================================================================

Sorted By             :      Signal
Multiplier            :      1.0000
Dilution              :      1.0000
Use Multiplier & Dilution Factor with ISTDs


Signal 1: DAD1 C, Sig=210,8 Ref=off

Peak RetTime Type  Width     Area      Height     Area
  #   [min]        [min]   [mAU*s]     [mAU]        %
----|-------|----|-------|----------|----------|--------|
   1  12.912 MM    0.5432   49.91383    1.53145   1.8964
   2  15.498 MM    0.5793 2582.10571   74.28701  98.1036

Totals :                  2632.01955   75.81846
```

Table 4 Entry 5

```
=====================================================================
Acq. Operator   : SYSTEM                        Seq. Line :   1
Acq. Instrument : HPLC                           Location : Vial 84
Injection Date  : 4/9/2021 3:18:02 PM                 Inj :   1
                                               Inj Volume : 10.000 µl
Different Inj Volume from Sample Entry!  Actual Inj Volume : 5.000 µl
Method          : C:\CHEM32\1\DATA\P15036\P15036-2021-04-09 2021-04-09 15-15-17\P15036_
                  CHIRALPAK_AS-3_90-10_N-HEXANE-IPA.M (Sequence Method)
Last changed    : 4/9/2021 3:15:18 PM by SYSTEM
Additional Info : Peak(s) manually integrated
```

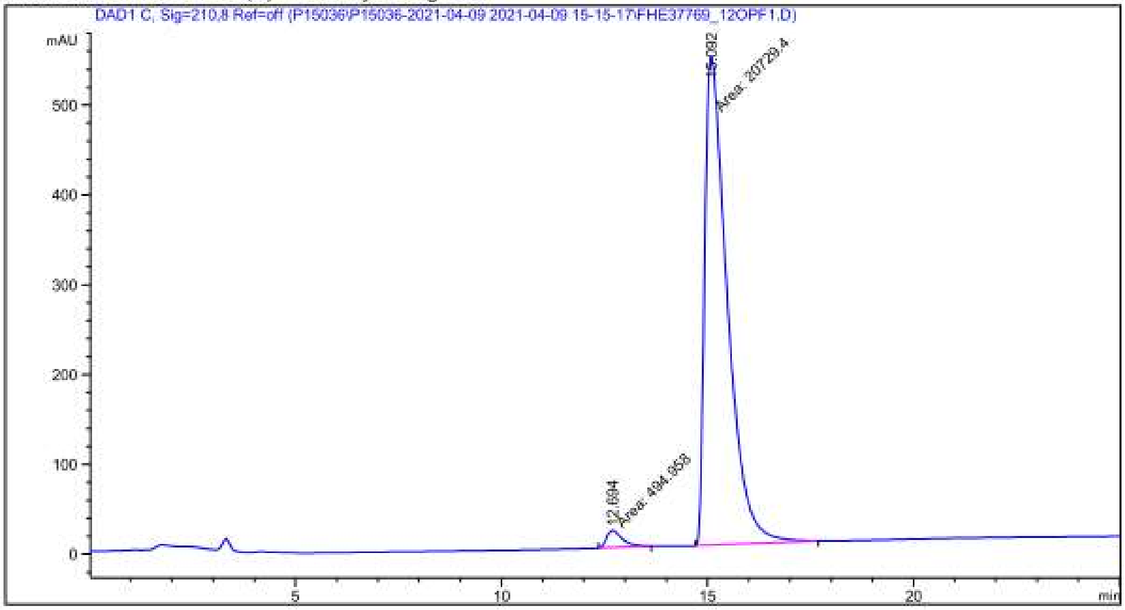


```
=====================================================================
                         Area Percent Report
=====================================================================

Sorted By             :      Signal
Multiplier            :      1.0000
Dilution              :      1.0000
Use Multiplier & Dilution Factor with ISTDs


Signal 1: DAD1 C, Sig=210,8 Ref=off

Peak RetTime Type  Width     Area      Height     Area
  #   [min]        [min]   [mAU*s]     [mAU]        %
----|-------|----|-------|----------|----------|--------|
   1  12.694 MM    0.4373  494.95840   18.86523   2.3320
   2  15.092 MM    0.6351 2.07294e4  544.02533  97.6680

Totals :                  2.12243e4  562.89056


=====================================================================
                         *** End of Report ***
```

Table 4 Entry 6

```
=====================================================================
Acq. Operator   : SYSTEM                        Seq. Line :   2
Acq. Instrument : HPLC                           Location : Vial 85
Injection Date  : 4/9/2021 3:44:35 PM                 Inj :   1
                                               Inj Volume : 10.000 µl
Different Inj Volume from Sample Entry!  Actual Inj Volume : 5.000 µl
Method          : C:\CHEM32\1\DATA\P15036\P15036-2021-04-09 2021-04-09 15-15-17\P15036_
                  CHIRALPAK_AS-3_90-10_N-HEXANE-IPA.M (Sequence Method)
Last changed    : 4/9/2021 3:15:18 PM by SYSTEM
Additional Info : Peak(s) manually integrated
```

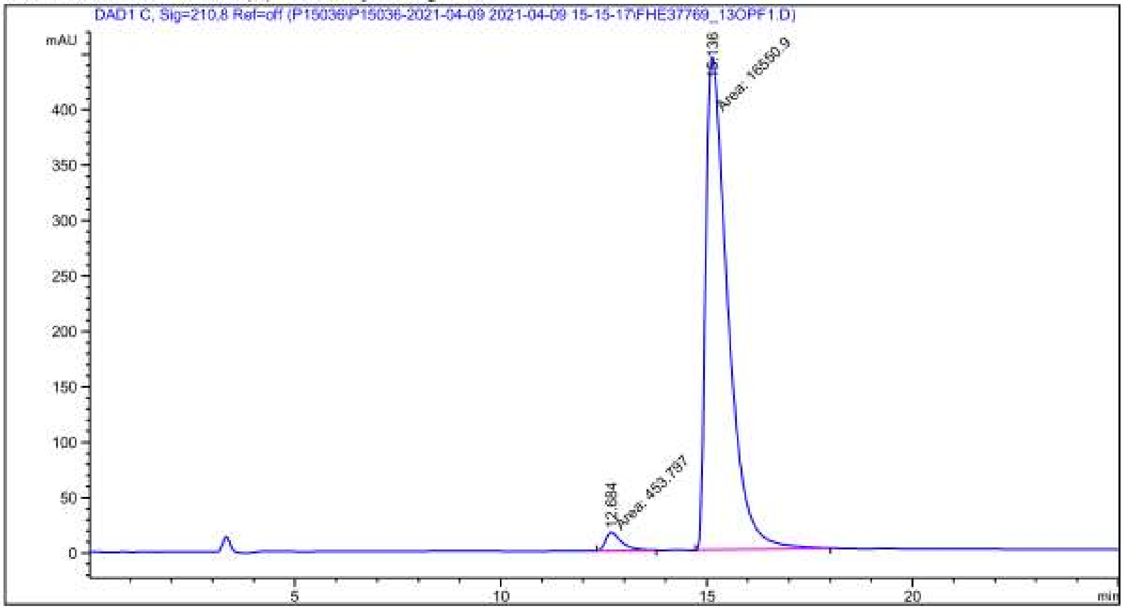


```
=====================================================================
                       Area Percent Report
=====================================================================

Sorted By             :      Signal
Multiplier            :      1.0000
Dilution              :      1.0000
Use Multiplier & Dilution Factor with ISTDs


Signal 1: DAD1 C, Sig=210,8 Ref=off

Peak RetTime Type  Width     Area      Height     Area
  #   [min]        [min]   [mAU*s]     [mAU]        %
----|-------|----|-------|----------|----------|--------|
   1  12.684 MM    0.4564  453.79651   16.57302   2.6687
   2  15.136 MM    0.6196 1.65509e4  445.21445  97.3313

Totals :                  1.70047e4  461.78747


=====================================================================
                        *** End of Report ***
```

Table 4 Entry 7

```
=====================================================================
Acq. Operator   : SYSTEM                        Seq. Line :   3
Acq. Instrument : HPLC                           Location : Vial 86
Injection Date  : 4/9/2021 4:11:08 PM                 Inj :   1
                                               Inj Volume : 10.000 µl
Different Inj Volume from Sample Entry!  Actual Inj Volume : 5.000 µl
Method          : C:\CHEM32\1\DATA\P15036\P15036-2021-04-09 2021-04-09 15-15-17\P15036_
                  CHIRALPAK_AS-3_90-10_N-HEXANE-IPA.M (Sequence Method)
Last changed    : 4/9/2021 3:15:18 PM by SYSTEM
Additional Info : Peak(s) manually integrated
```

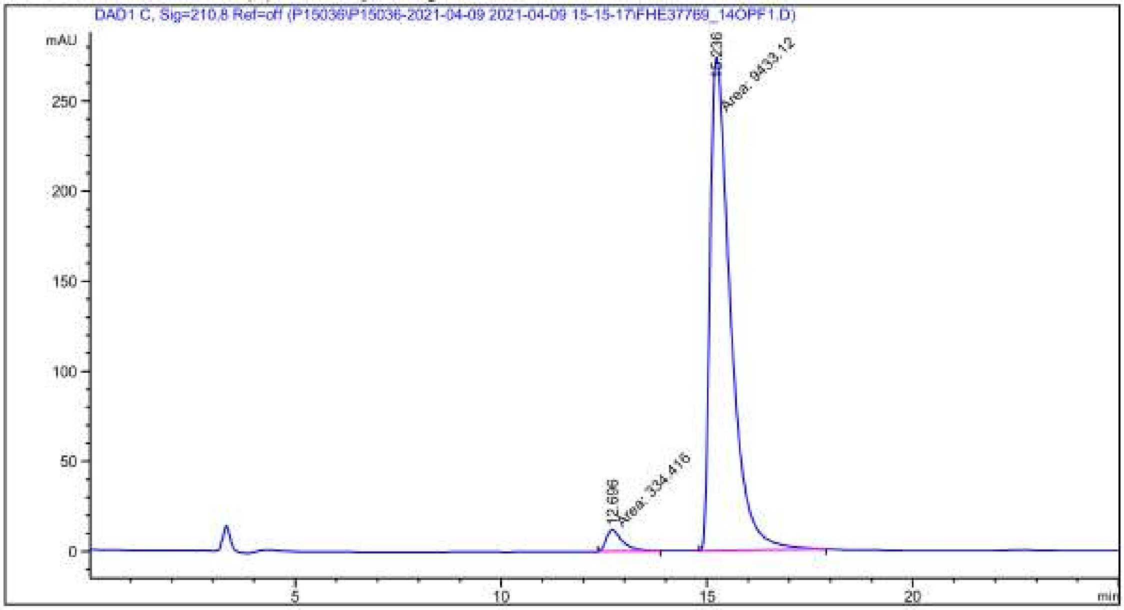


```
=====================================================================
                         Area Percent Report
=====================================================================

Sorted By             :      Signal
Multiplier            :      1.0000
Dilution              :      1.0000
Use Multiplier & Dilution Factor with ISTDs


Signal 1: DAD1 C, Sig=210,8 Ref=off

Peak RetTime Type  Width     Area      Height     Area
  #   [min]        [min]   [mAU*s]     [mAU]        %
----|-------|----|-------|----------|----------|--------|
   1  12.696 MM    0.4622  334.41614   12.05796   3.4238
   2  15.236 MM    0.5726 9433.12305  274.59280  96.5762

Totals :                  9767.53918  286.65077


=====================================================================
                          *** End of Report ***
```

Table S5

Table S5 Entry 1

```
=====================================================================
Acq. Operator   : SYSTEM                          Seq. Line :   4
Acq. Instrument : HPLC                             Location : Vial 45
Injection Date  : 4/8/2021 4:52:17 PM                   Inj :   1
                                                 Inj Volume : 10.000 µl
Different Inj Volume from Sample Entry!  Actual Inj Volume : 5.000 µl
Acq. Method     : C:\CHEM32\1\DATA\P15036\P15036 2021-04-08 15-30-38\P15036_CHIRALPACK_IA_90-
                  10.M
Last changed    : 4/8/2021 3:47:31 PM by SYSTEM
Analysis Method : C:\CHEM32\1\DATA\P15036\P15036 2021-04-08 15-30-38\P15036_CHIRALPACK_IA_90-
                  10.M (Sequence Method)
Last changed    : 4/8/2021 3:30:38 PM by SYSTEM
Additional Info : Peak(s) manually integrated
```

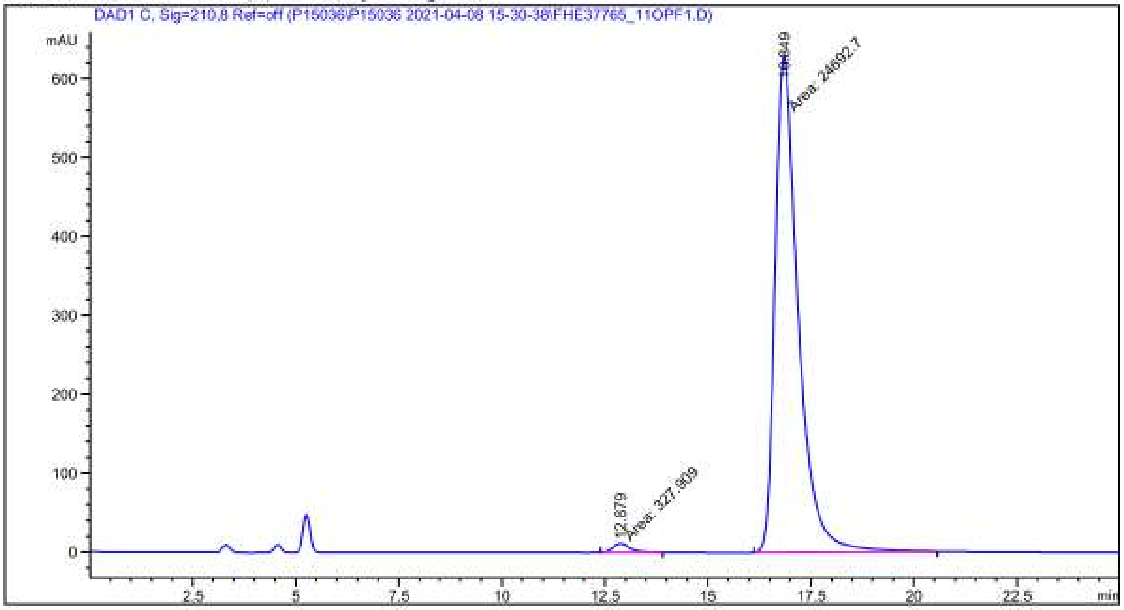


```
=====================================================================
                         Area Percent Report
=====================================================================

Sorted By             :      Signal
Multiplier            :      1.0000
Dilution              :      1.0000
Use Multiplier & Dilution Factor with ISTDs


Signal 1: DAD1 C, Sig=210,8 Ref=off

Peak RetTime Type  Width     Area      Height     Area
  #   [min]        [min]   [mAU*s]     [mAU]        %
----|-------|----|-------|----------|----------|--------|
   1  12.879 MM    0.4787  327.90881   11.41730   1.3106
   2  16.849 MM    0.6540 2.46927e4   629.31458  98.6894

Totals :                  2.50206e4   640.73188
```

Table S5 Entry 2

```
=====================================================================
Acq. Operator   : SYSTEM                        Seq. Line :   5
Acq. Instrument : HPLC                           Location : Vial 46
Injection Date  : 4/8/2021 5:18:57 PM                 Inj :   1
                                               Inj Volume : 10.000 µl
Different Inj Volume from Sample Entry!  Actual Inj Volume : 5.000 µl
Acq. Method     : C:\CHEM32\1\DATA\P15036\P15036 2021-04-08 15-30-38\P15036_CHIRALPACK_IA_90-
                  10.M
Last changed    : 4/8/2021 3:47:31 PM by SYSTEM
Analysis Method : C:\CHEM32\1\DATA\P15036\P15036 2021-04-08 15-30-38\P15036_CHIRALPACK_IA_90-
                  10.M (Sequence Method)
Last changed    : 4/8/2021 3:30:38 PM by SYSTEM
Additional Info : Peak(s) manually integrated
```

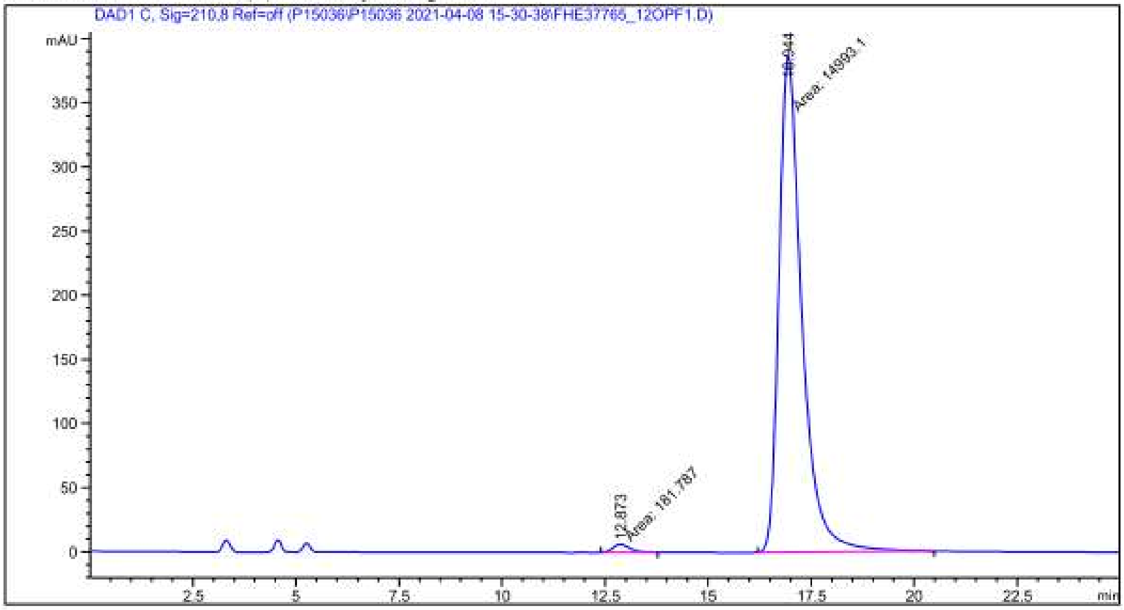


```
=====================================================================
                         Area Percent Report
=====================================================================

Sorted By             :      Signal
Multiplier            :      1.0000
Dilution              :      1.0000
Use Multiplier & Dilution Factor with ISTDs


Signal 1: DAD1 C, Sig=210,8 Ref=off
```

Peak #	RetTime [min]	Type	Width [min]	Area [mAU*s]	Height [mAU]	Area %
1	12.873	MM	0.4755	181.78728	6.37164	1.1979
2	16.944	MM	0.6463	1.49931e4	386.61841	98.8021

Totals : 1.51749e4 392.99004

Table S5 Entry 3

```
=====================================================================
Acq. Operator   : SYSTEM                        Seq. Line :   6
Acq. Instrument : HPLC                           Location : Vial 47
Injection Date  : 4/8/2021 5:45:35 PM                 Inj :   1
                                               Inj Volume : 10.000 µl
Different Inj Volume from Sample Entry!  Actual Inj Volume : 5.000 µl
Acq. Method     : C:\CHEM32\1\DATA\P15036\P15036 2021-04-08 15-30-38\P15036_CHIRALPACK_IA_90-
                  10.M
Last changed    : 4/8/2021 3:47:31 PM by SYSTEM
Analysis Method : C:\CHEM32\1\DATA\P15036\P15036 2021-04-08 15-30-38\P15036_CHIRALPACK_IA_90-
                  10.M (Sequence Method)
Last changed    : 4/8/2021 3:30:38 PM by SYSTEM
Additional Info : Peak(s) manually integrated
```

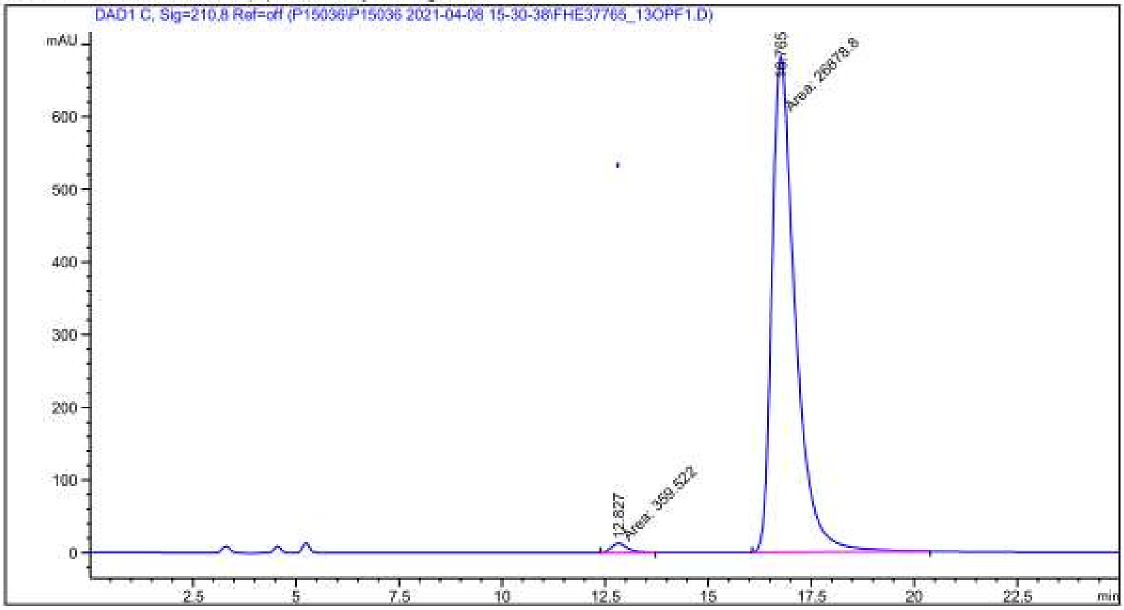


```
=====================================================================
                     Area Percent Report
=====================================================================

Sorted By             :      Signal
Multiplier            :      1.0000
Dilution              :      1.0000
Use Multiplier & Dilution Factor with ISTDs


Signal 1: DAD1 C, Sig=210,8 Ref=off

Peak RetTime Type  Width     Area      Height     Area
  #   [min]        [min]   [mAU*s]     [mAU]       %
----|-------|----|-------|----------|----------|--------|
   1  12.827 MM    0.4639  359.52225   12.91746   1.3199
   2  16.765 MM    0.6554 2.68788e4  683.56158  98.6801

Totals :                  2.72384e4  696.47905
```

Table S5 Entry 4

```
=====================================================================
Acq. Operator   : SYSTEM                          Seq. Line :   1
Acq. Instrument : HPLC                             Location : Vial 87
Injection Date  : 4/12/2021 8:14:07 AM                  Inj :   1
                                                 Inj Volume : 10.000 µl
Method          : C:\CHEM32\1\DATA\P15036\P15036-2021-04-09 2021-04-12 08-12-38\P15036_
                  CHIRALPAK_AS-3_90-10_N-HEXANE-IPA.M (Sequence Method)
Last changed    : 4/12/2021 8:12:38 AM by SYSTEM
Additional Info : Peak(s) manually integrated
```

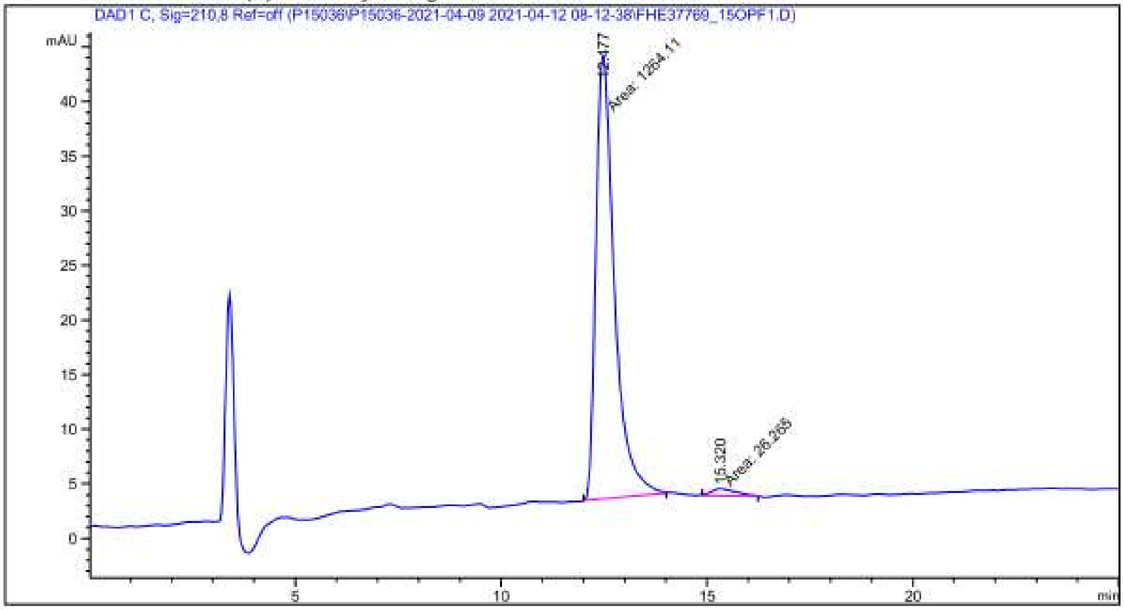


```
=====================================================================
                         Area Percent Report
=====================================================================

Sorted By             :      Signal
Multiplier            :      1.0000
Dilution              :      1.0000
Use Multiplier & Dilution Factor with ISTDs


Signal 1: DAD1 C, Sig=210,8 Ref=off

Peak RetTime Type  Width     Area      Height     Area
  #   [min]        [min]   [mAU*s]     [mAU]        %
----|-------|----|-------|----------|----------|--------|
   1  12.477 MM    0.5191 1264.11487   40.58566  97.9646
   2  15.320 MM    0.6788   26.26501 6.44887e-1   2.0354

Totals :                  1290.37988   41.23054


=====================================================================
                          *** End of Report ***
```

Table S5 Entry 5

```
=====================================================================
Acq. Operator   : SYSTEM                        Seq. Line :   5
Acq. Instrument : HPLC                           Location : Vial 88
Injection Date  : 4/9/2021 5:04:18 PM                 Inj :   1
                                               Inj Volume : 10.000 µl
Different Inj Volume from Sample Entry!  Actual Inj Volume : 5.000 µl
Acq. Method     : C:\CHEM32\1\DATA\P15036\P15036-2021-04-09 2021-04-09 15-15-17\P15036_
                  CHIRALPAK_AS-3_90-10_N-HEXANE-IPA.M
Last changed    : 4/9/2021 3:15:18 PM by SYSTEM
Analysis Method : C:\CHEM32\1\DATA\P15036\P15036-2021-04-09 2021-04-09 15-15-17\P15036_
                  CHIRALPAK_AS-3_90-10_N-HEXANE-IPA.M (Sequence Method)
Last changed    : 4/12/2021 7:43:32 AM by SYSTEM
                  (modified after loading)
Additional Info : Peak(s) manually integrated
```

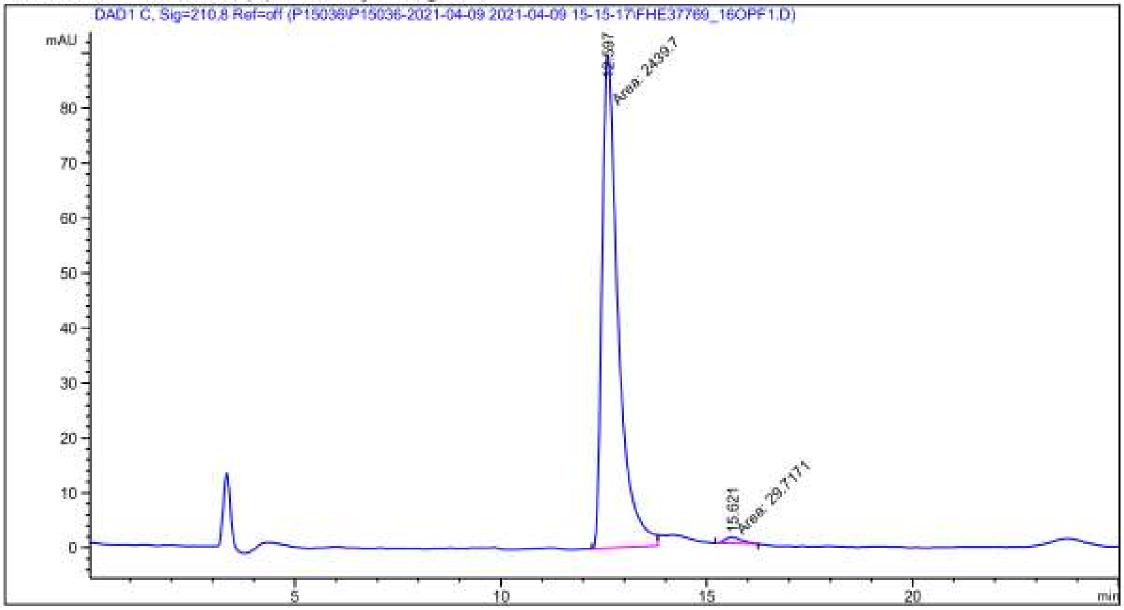


```
=====================================================================
                         Area Percent Report
=====================================================================

Sorted By             :      Signal
Multiplier            :      1.0000
Dilution              :      1.0000
Use Multiplier & Dilution Factor with ISTDs


Signal 1: DAD1 C, Sig=210,8 Ref=off

Peak RetTime Type  Width     Area      Height     Area
  #   [min]        [min]   [mAU*s]     [mAU]        %
----|-------|----|-------|----------|----------|--------|
   1  12.597 MF    0.4527 2439.69971   89.81717  98.7966
   2  15.621 MM    0.4504   29.71712    1.09967   1.2034

Totals :                  2469.41682   90.91684
```

Table S5 Entry 6

```
=====================================================================
Acq. Operator   : SYSTEM                        Seq. Line :   6
Acq. Instrument : HPLC                           Location : Vial 89
Injection Date  : 4/9/2021 5:30:53 PM                 Inj :   1
                                               Inj Volume : 10.000 µl
Different Inj Volume from Sample Entry!  Actual Inj Volume : 5.000 µl
Acq. Method     : C:\CHEM32\1\DATA\P15036\P15036-2021-04-09 2021-04-09 15-15-17\P15036_
                  CHIRALPAK_AS-3_90-10_N-HEXANE-IPA.M
Last changed    : 4/9/2021 3:15:18 PM by SYSTEM
Analysis Method : C:\CHEM32\1\DATA\P15036\P15036-2021-04-09 2021-04-09 15-15-17\P15036_
                  CHIRALPAK_AS-3_90-10_N-HEXANE-IPA.M (Sequence Method)
Last changed    : 4/12/2021 7:43:32 AM by SYSTEM
                  (modified after loading)
Additional Info : Peak(s) manually integrated
```

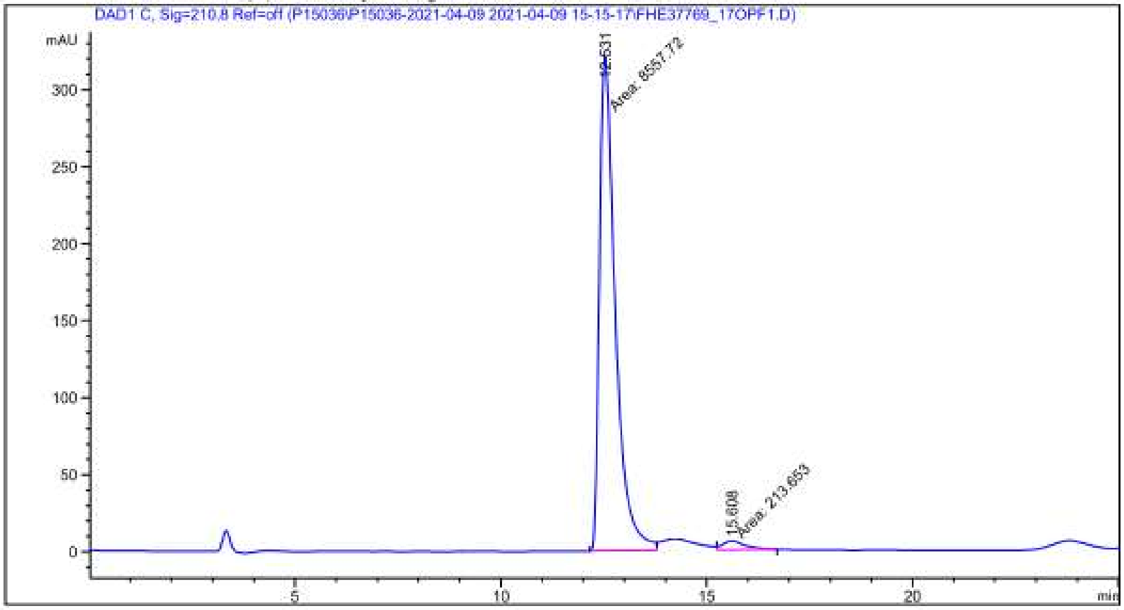


```
=====================================================================
                         Area Percent Report
=====================================================================

Sorted By             :      Signal
Multiplier            :      1.0000
Dilution              :      1.0000
Use Multiplier & Dilution Factor with ISTDs


Signal 1: DAD1 C, Sig=210,8 Ref=off
```

Peak #	RetTime [min]	Type	Width [min]	Area [mAU*s]	Height [mAU]	Area %
1	12.531	MF	0.4437	8557.71680	321.47260	97.5642
2	15.608	FM	0.6262	213.65251	5.68635	2.4358

Totals : 8771.36931 327.15894

Chiral HPLC traces for API

```
Data file  : C:\CHEM32\1\DATA\NICCOLO'\NN-91-F2-PTLC.D
Sample Name: NN-91-F2-PTLC                                                   1
===========================================================================
Injection Date  : Tue, 18. May. 2021            Seq Line   :          0
Sample Name     : NN-91-F2-PTLC                 Location   :     Vial 1
Acq Operator    : MILENA                        Inj. No.   :          0
NN-91-F2-PTLC
PHENOMENEX Hex 7: EtOH 3 : 0,1 DEA 1mL/min
```

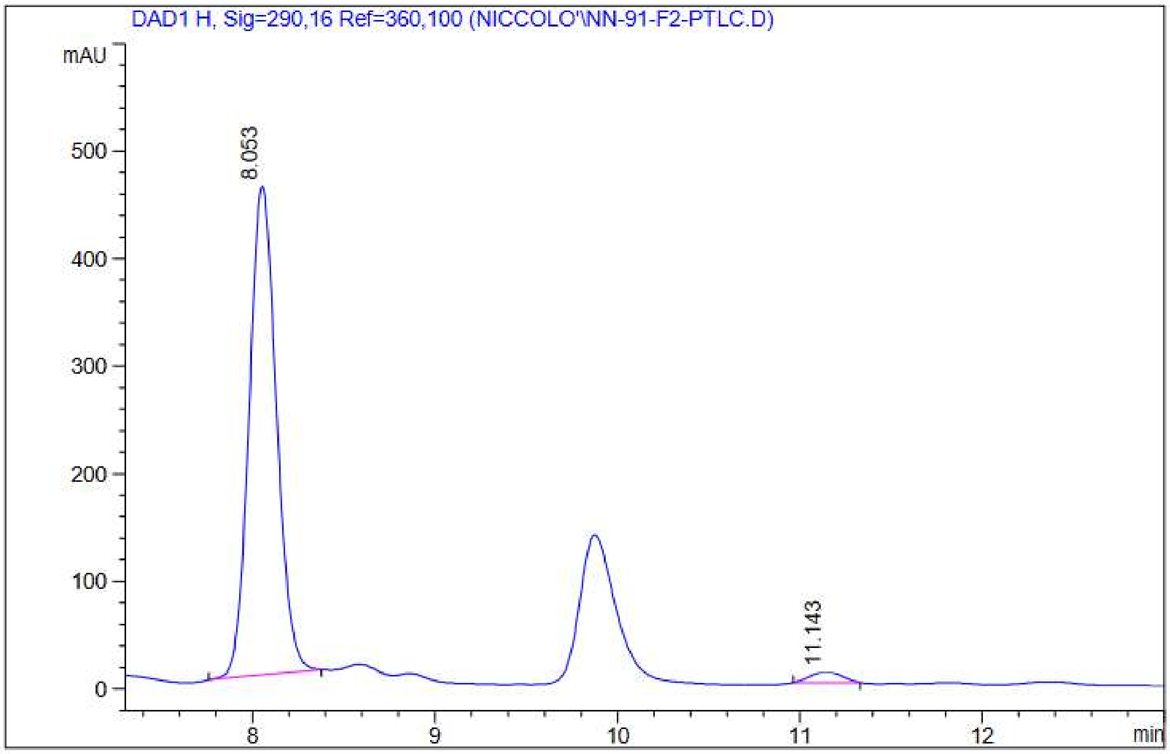

Signal 1: DAD1 H, Sig=290,16 Ref=360,100

Peak #	RT [min]	Type	Width [min]	Area	Area %	Name
1	8.053	MM	0.173	4720.577	97.462	
2	11.143	MM	0.203	122.907	2.538	

Racemic HPLC traces for API

```
Data file   : C:\CHEM32\1\DATA\NICCOLO'\NN-59-A1-3.D
Sample Name: NN-59-A-3                                             1
=====================================================================
Injection Date  : Thu, 22. Apr. 2021          Seq Line   :        0
Sample Name     : NN-59-A-3                   Location   :   Vial 1
Acq Operator    : NICCOLO'                    Inj. No.   :        0
NN-59-6
PHENOMENEX LUX 3u Amylose-1, 70 Hex 30 EtOH 0,1 DEA, 1
mL/min, P=97 bar
```

->

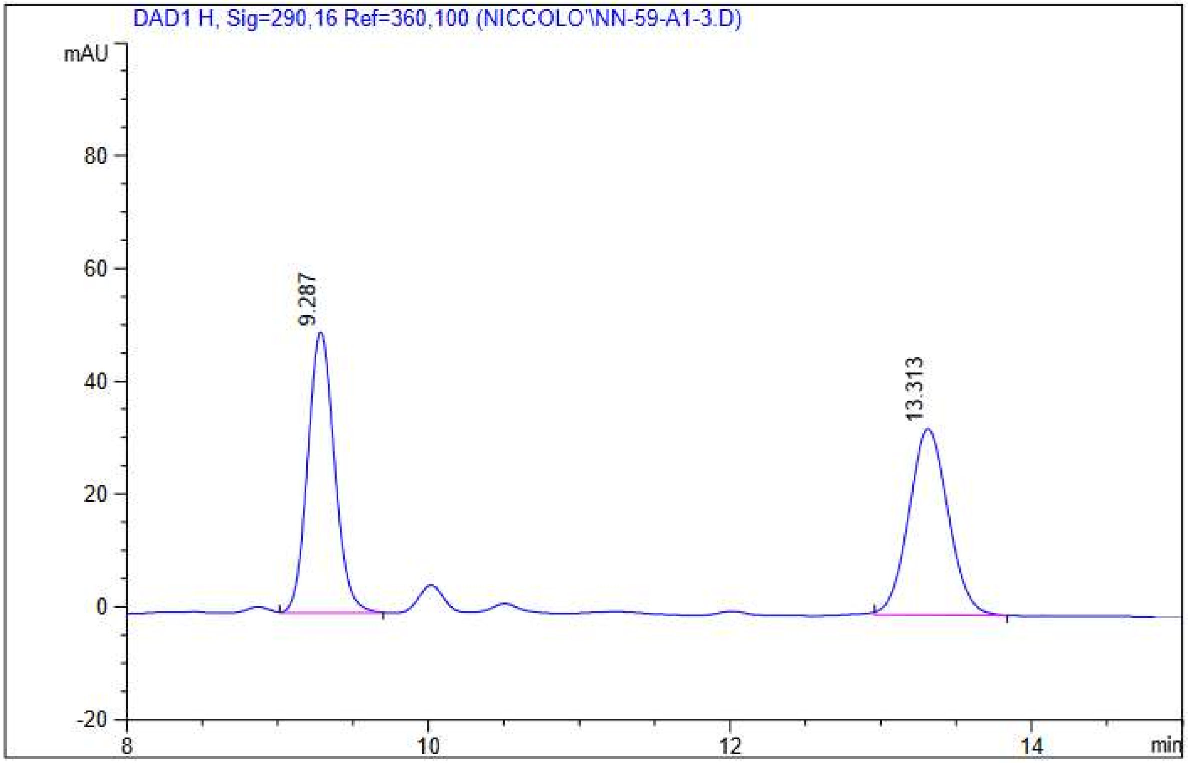

Signal 1: DAD1 H, Sig=290,16 Ref=360,100

Peak #	RT [min]	Type	Width [min]	Area	Area %	Name
1	9.287	VB	0.190	615.802	50.452	
2	13.313	BB	0.283	604.764	49.548	
